# BRG1-mediated suppression of ferroptosis underlies BTK inhibitor resistance

**DOI:** 10.1038/s41467-026-75123-4

**Published:** 2026-07-02

**Authors:** Soo-Yeon Hwang, Xiangao Huang, Helgi Nikolli, Belem Yoval-Sánchez, Sieun Yang, Jeongbin Heo, Peter Martin, Caitlin Gribbin, Preetesh Jain, Michael Wang, Lalit Sehgal, Robert A. Baiocchi, Inah Hwang, Maurizio DiLiberto, Alexander Galkin, Hojoong Kwak, Lapo Alinari, Selina Chen-Kiang, Hongwu Zheng, Jihye Paik

**Affiliations:** 1 Department of Pathology and Laboratory Medicine, Weil Cornell Medicine, New York, NY USA; 2 Meyer Cancer Center, Weill Cornell Medicine and New York Presbyterian Hospital, New York, NY USA; 3 Feil Family Brain and Mind Research Institute, Weill Cornell Medicine, New York, NY USA; 4 Graduate School of Pharmaceutical Sciences and College of Pharmacy, Ewha Womans University, Seoul, Republic of Korea; 5 Graduate Program in Innovative Biomaterials Convergence, Ewha Womans University, Seoul, Republic of Korea; 6 Department of Cell Biology and Anatomy, New York Medical College, Valhalla, NY USA; 7 Division of Hematology and Medical Oncology, Perlmutter Cancer Center, NYU Langone, New York, NY USA; 8 Division of Hematology and Medical Oncology, Weill Cornell Medicine, New York, NY USA; 9 Mays Cancer Center, University of Texas Health San Antonio, San Antonio, TX USA; 10 Department of Lymphoma and Myeloma from The University of Texas MD Anderson Cancer Center, Houston, TX USA; 11 Division of Hematology, Department of Internal Medicine, College of Medicine, The Ohio State University, Columbus, OH USA

**Keywords:** Cancer therapeutic resistance, Haematological cancer, Cell death

## Abstract

Resistance to Bruton’s tyrosine kinase inhibitors (BTKi) remains a major therapeutic challenge in B-cell malignancies. Here, we identify chromatin remodeler BRG1-mediated suppression of ferroptosis as a central mechanism of BTKi resistance in mantle cell lymphoma (MCL), in which aberrant BRG1-dependent transcription program protects cells from BTKi-induced ferroptosis by restricting reactive oxygen species (ROS) and labile iron. Mechanistically, BRG1 promotes resistance through regulation of both BTK-dependent survival signaling and a BTK-independent transcriptional program. The latter is mediated by BRG1-driven induction of MEF2B, which upregulates atypical mitochondrial complex I subunit NDUFA4L2. Increased NDUFA4L2 restricts cellular respiration, preemptively limiting mitochondrial ROS generation and activating AMPK signaling, together reducing susceptibility to lipid peroxidation and ferroptosis. Pharmacologic inhibition of BRG1 disrupts these programs, restoring ferroptotic sensitivity and synergizing with BTKi across resistant MCL models. Together, our study establishes BRG1 as a central regulator of BTKi resistance and provides a rationale for co-targeting BRG1 and BTK as a therapeutic strategy for B-cell malignancies.

## Abstract (web summary)

While the introduction of Bruton’s tyrosine kinase inhibitors (BTKi) have improved outcomes for patients with B-cell malignancies, resistance often develops. Here, the authors report that the chromatin remodeler BRG1 promotes resistance to BTKi by regulating transcriptional programs which reduce reactive oxygen species and labile iron, in turn protecting tumour cells from ferroptosis and promoting resistance.

## Introduction

B-cell malignancies remain largely incurable diseases with substantial clinical challenges^1^. Bruton’s tyrosine kinase inhibitors (BTKi), first approved a decade ago, revolutionized the treatment landscape by targeting a key intermediary in the B-cell receptor (BCR) signaling pathway. BTKi have significantly improved outcomes in chronic lymphocytic leukemia, mantle cell lymphoma (MCL), Waldenström’s macroglobulinemia, marginal zone lymphoma, follicular lymphoma, as well as subtypes of diffuse large B-cell lymphoma^2–6^. In MCL, BTKi are now integral to frontline treatment and are associated with improvements in progression-free and overall survival^7–9^. However, in the relapsed/refractory setting, responses are often transient, with most patients developing resistance within 1–2 years and a median survival of only a few months following ibrutinib (IB) failure^7,10^. As BTKi moves earlier into the treatment course, the incidence of BTKi-refractory MCL is expected to rise. Defining the molecular mechanisms underlying resistance development is therefore essential for designing effective therapeutic strategies.

Resistance to BTKi in MCL can arise from diverse mechanisms, including genetic lesions (e.g., *BTK*, *PLCγ2*, *BIRC3*, *TRAF2/3*, or *CARD11* mutations), adaptive pathway reactivation (e.g., NF-κB, PI3K/AKT/mTORC1, or MAPK), metabolic rewiring toward oxidative phosphorylation or tumor microenvironment-derived cues that compensate for the BTK inhibition^11,12^. To overcome the resistance, various strategies, including targeted therapeutics and immunotherapies, have been explored either alone or in combination with BTKi^13,14^. However, despite clinical benefits, treatment-limiting toxicities and the frequent emergence of resistance remain major challenges, underscoring the need to identify novel therapeutic vulnerabilities beyond currently targeted pathways. Ferroptosis, a regulated form of cell death characterized by iron-dependent lipid peroxidation, has recently emerged as a mechanistically distinct vulnerability in cancer^15–17^. Its reliance on cellular redox balance directly intersects with metabolic reprogramming pathways implicated in BTKi resistance^12,18^, raising the possibility that its regulation may represent an alternative therapeutic opportunity in MCL.

BTKi resistance in MCL is rarely explained by genetic alterations in BTK itself, suggesting alternative mechanisms such as epigenetic reprogramming^19^. Recurrent mutations in chromatin regulators, altered chromatin landscapes, and emerging dependencies on specific transcriptional networks^20,21^, point to epigenetic dysregulation as a central driver of therapeutic resistance. BRG1 (encoded by *SMARCA4*) is a catalytic subunit of the mammalian BAF (SWI/SNF) nucleosome remodeling complex that regulates gene expression programs controlling proliferation, differentiation, DNA repair, stress responses, and therapy resistance^22–26^. Its genetic alterations occur in ~5–7% of human cancers^27,28^, exhibiting context-dependent roles as either a tumor suppressor or oncogene^29–32^. In B-cell lymphomas, BRG1 frequently exhibits missense mutations or haploinsufficiency, while complete deletion is rare^33,34^. In Burkitt lymphoma, its heterozygous missense mutations were found in 27% of cases^35^. In genetic studies using mice, BRG1 haploinsufficiency drives lymphomagenesis from germinal center centrocytes by disrupting transcriptional activity of SPI1, NF-κB, and IRF family, promoting a hyperproliferative state through BCL6 and MYC activation^33^. In MCL, BRG1 forms a complex with SOX11 to activate oncogenic transcriptional programs that directly regulate the expression of key genes involved in disease pathogenesis^36^. BRG1 mutations are enriched in BTKi-refractory MCL, present in 50% of IB-venetoclax resistant cases^37,38^. Despite its recurrent alteration and functional involvement in MCL pathogenesis and treatment failure, it remains unclear how the BRG1-dependent transcription program promotes BTKi resistance.

In this study, we delineate a BRG1-mediated mechanism of BTKi resistance in MCL rooted in ferroptosis suppression. We discovered that cells expressing cancer-associated BRG1 mutants escape BTKi-induced ferroptosis by restricting intracellular labile iron and reactive oxygen species (ROS). This protection is mediated through both BTK-dependent signaling and a BRG1-MEF2B-NDUFA4L2 pathway that blocks mitochondria-dependent ferroptosis. Functionally, BRG1 sustains BCR survival signaling and MEF2B expression, while MEF2B-driven induction of NDUFA4L2 impairs mitochondrial activity to promote ferroptosis resistance. Importantly, we demonstrate that pharmacologic BRG1 inhibition restores ferroptotic sensitivity and synergizes with BTKi across resistant MCL models, supporting BRG1 as a central regulator of BTKi resistance and a promising therapeutic target.

## Results

### Suppression of ferroptosis underlies BTK inhibitor resistance

**Fig. 1:**
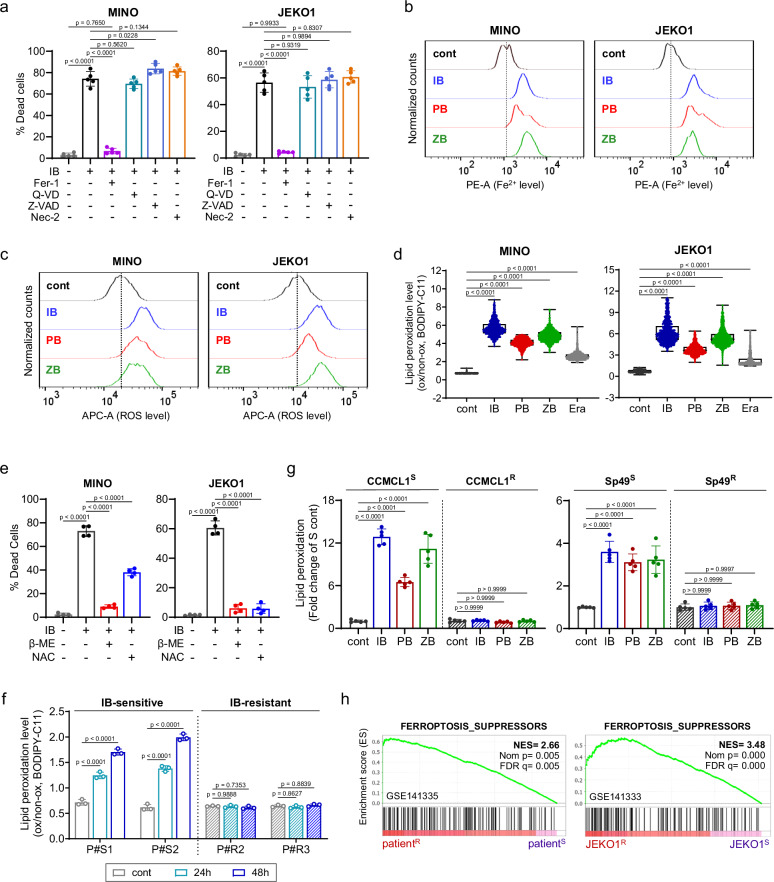
Ferroptosis suppression underlies BTK inhibitor resistance. **a** Effect of cell death inhibitors (Fer-1, 2 μM; Q-VD-Oph, 10 μM; Z-VAD-FMK, 10 μM; Necrostatin-2, 10 μM) on IB-induced (96 h) cell death in MINO and JEKO1 cells. Representative flow cytometry histograms showing changes in labile Fe^2+^ levels (**b**) and ROS (**c**) following BTKi treatment (48 h) from three independent experiments with similar results. **d** BTKi-induced changes in lipid peroxidation (72 h) in BRG1^WT^ MCL cells. Erastin (0.5 μM) was used as positive control. **e** Effect of antioxidants (β-mercaptoethanol and N-acetylcysteine; 100 μM) against IB-induced (96 h) cell death in BRG1^WT^ MCL cells. **f** Time-dependent changes in the lipid peroxidation level upon IB treatment in primary MCL cells from IB-responsive or refractory patients. **g** BTKi-induced changes in lipid peroxidation (24 h) in isogenic BTKi^S^ and BTKi^R^ CCMCL1 and Sp49 cells. **h** GSEA plots for curated Ferroptosis_Suppressors gene set comparing IB-resistant (patient^R^) vs. -sensitive (patient^S^) MCL patients (GSE141335) and JEKO1^R^ vs. JEKO1^S^ cells (GSE141333). All BTKi treatments were performed at 5 μM. Quantitative data are from *n* = 5 biologically independent experiments, except *n* = 4 biologically independent experiments in (**e**). In (**f**), *n* = 4 independent patient-derived primary MCL samples were analyzed, each in triplicate. Bar plots represent mean ± s.d.; Box plots in (**d**) show single-cell flow cytometry events from a representative experiment; center line indicates the median, box bounds indicate the 25th and 75th percentiles, and whiskers indicate minimum and maximum values. The experiment was repeated independently five times with similar results. All statistical significances were determined by one-way ANOVA with Tukey’s test.

To investigate the mechanism underlying BTKi treatment-induced MCL cell death, MINO and JEKO1 cells were co-treated with BTKi (ibrutinib, pirtobrutinib, or zanubrutinib; IB, PB, ZB) together with inhibitors of specific cell death pathways. Among them, the synthetic ferroptosis inhibitor ferrostatin-1 (Fer-1) most significantly suppressed BTKi-induced cell death (BTKi vs. BTKi+Fer-1; 41.2–73.3 vs. 5.8–6.7% in MINO; 44.9–89.4 vs. 4.4–13.2% in JEKO1) (Fig. 1a, Supplementary Fig. 1a). By contrast, co-treatment with the pan-caspase inhibitors Z-VAD-FMK or Q-VD-OPh, or the necroptosis inhibitor necrostatin-2, produced only minor effects (Fig. 1a). These results suggest ferroptosis as a primary pathway underlying BTKi-induced MCL cell death. Consistently, BTKi treatment markedly enhanced intracellular labile Fe²⁺ levels, a condition conducive to ferroptosis (Fig. 1b). Treated cells also exhibited elevated total ROS levels (Fig. 1c) and significantly increased lipid peroxidation (up to ~7-fold in MINO and ~10-fold in JEKO1) (Fig. 1d), consistent with the accumulation of iron-dependent oxidative damage, a hallmark of ferroptotic cell death. This was further supported by the observation that treatment with antioxidants β-mercaptoethanol or N-acetylcysteine markedly suppressed IB-induced cell death (Fig. 1e). Moreover, CRISPR-mediated BTK depletion also significantly elevated cellular levels of labile Fe²⁺, total ROS, and lipid peroxidation (Supplementary Fig. 1b–e), further supporting a protective role of BTK activity against ferroptosis.

To determine whether suppression of ferroptosis contributes to BTKi resistance development in MCL patients, we next examined primary MCL samples from IB-responsive and -refractory patients by assessing lipid peroxidation level following IB treatment. Remarkably, whereas IB treatment-induced robust lipid peroxidation in primary tumor cells from responsive patients, refractory samples exhibited minimal responses (Fig. 1f). A similar pattern was also observed in isogenic BTKi-sensitive (S) and -resistant (R) CCMCL1 and Sp49 cell lines, with strong lipid peroxidation induction in sensitive cells but minimal responses in their resistant counterparts (Fig. 1g). Furthermore, gene set enrichment analysis (GSEA) of primary MCL cells from IB-resistant vs. -sensitive patients^21^ revealed significant enrichment of ferroptosis suppressors gene set, a pattern recapitulated in JEKO1^R^ vs. JEKO1^S^ cells (Fig. 1h, Supplementary Data 1). Together, these findings indicate that ferroptosis suppression is a major mechanism underlying BTKi resistance.

### BRG1 regulates ferroptosis response in MCL cells

**Fig. 2:**
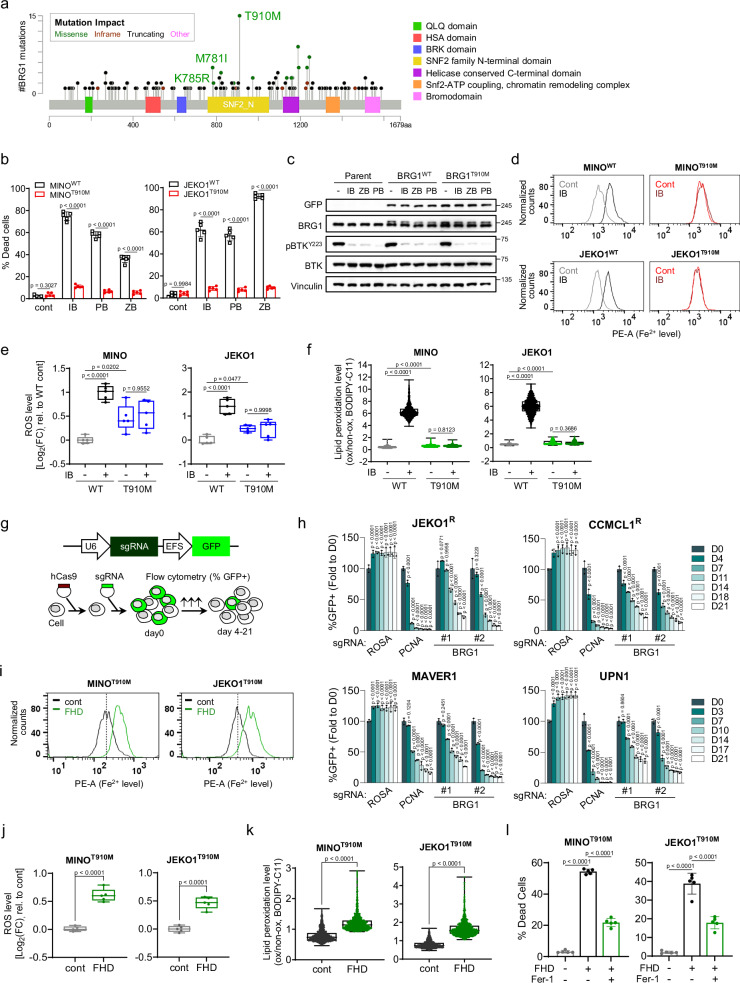
BRG1 regulates ferroptosis response in MCL cells. **a** Schematic diagrams summarizing the distribution of mutations along the BRG1 sequence (http://oncokb.org). Mutation diagram circles are colored with respect to the corresponding mutation types. **b** Cell death response to various BTKi in BRG1^WT^ and BRG1^T910M^ MCL cell lines (96 h). **c** Immunoblot analysis of MINO^Parent^, MINO^WT^, and MINO^T910M^ cells following BTKi treatment (1 h). IB-induced changes in labile Fe^2+^ (**d**; 48 h), ROS (**e**; 48 h), and lipid peroxidation (**f**; 72 h) in BRG1^WT^ vs. BRG1^T910M^ MCL cells. **g** Experimental scheme for the competition-based GFP dropout proliferation assay. **h** Competition-based proliferation assays with BRG1-targeting sgRNAs in Cas9-transduced CCMCL1^R^, MAVER1, JEKO1^R^, and UPN1 cells. sgROSA and sgPCNA were used as a negative and positive control, respectively. **i** Representative histograms showing labile Fe^2+^ level changes in BRG1^T910M^ cell lines following FHD-286 treatment (48 h). FHD-286-induced changes in ROS (**j**; 48 h), and lipid peroxidation (**k**; 72 h) in BRG1^T910M^ MCL cell lines. **l** FHD-286 (96 h)-induced cell death response with or without Fer-1 (2 μM) in BRG1^T910M^ MCL cell lines. All BTKi and FHD-286 treatments were performed at 5 μM and 10 nM, respectively. Representative histograms in (**d**, **i**) are from five independent experiments with similar results. Quantitative data are from *n* = 5 biologically independent experiments, except *n* = 3 biologically independent experiments in (**h**). Bar plots represent mean ± s.d.; box plots in (**e**, **f**, **j**, **k**) indicate medians as center lines, 25th and 75th percentiles as box bounds, and minimum and maximum values as whiskers. Points in (**f**, **k**) represent single-cell flow cytometry events from representative experiments repeated independently five times with similar results. Statistical significance in (**j**, **k**) was determined using unpaired two-sided Student’s t-tests; all other comparisons were analyzed using one-way or two-way ANOVA followed by Tukey’s test.

As one of the frequently mutated genes in MCL, the SWI/SNF chromatin regulator BRG1 has been implicated in clinical resistance to BTKi^37^. To test its potential contribution to BTKi resistance, we generated paired isogenic cell lines by stably integrating either wild-type or T910M mutant BRG1, a recurrent hot spot mutation reported across multiple cancer types, including IB-resistant MCL (Fig. 2a, Supplementary Fig. 2a)^27,39^. Compared with BRG1^WT^-expressing controls, the BRG1^T910M^-expressing MCL cells exhibited notably greater resistance to both covalent (IB, ZB) and reversible (PB) BTKi (Fig. 2b, Supplementary Fig. 2b), despite comparable suppression of BTK^Y233^ phosphorylation (Fig. 2c). Importantly, in contrast to BRG1^WT^ cells, in which IB treatment led to robust elevation of labile Fe²⁺, ROS, and lipid peroxidation, BRG1^T910M^ cells failed to elicit a significant ferroptotic response upon IB treatment despite elevated baseline levels of Fe^2+^ and ROS (Fig. 2d, f), suggesting resistance to ferroptosis. Introduction of another MCL-associated mutant, BRG1^K785R^ (Fig. 2a, Supplementary Fig. 2a), similarly supported the role for BRG1 mutations in conferring resistance to IB-induced lipid peroxidation and cell death (Supplementary Fig. 2c, d). Given the role of BRG1 as a chromatin remodeler, we hypothesized that aberrant BRG1 may promote BTKi resistance by transcriptionally suppressing ferroptosis. Notably, BRG1^T910M^ cells showed significant enrichment of ferroptosis suppressors gene set compared to paired BRG1^WT^ controls (Supplementary Fig. 2e), providing transcriptional evidence that this isogenic system captures the oncogenic consequences of aberrant BRG1 activity and is well-suited for mechanistic dissection of BRG1-driven resistance.

In support of this idea, a CRISPR/Cas9-based vulnerability screen targeting 182 epigenetic regulators with ~1461 sgRNAs^40,41^ uncovered that multiple BTKi-resistant MCL cell lines were highly dependent on BRG1 (Supplementary Fig. 2f, Supplementary Data 2). This dependency was further validated in competition-based proliferation assays using independent BRG1-targeting sgRNAs (Fig. 2g, h, Supplementary Fig. 2g). Moreover, inhibition of BRG1 in BRG1^T910M^-expressing cells by FHD-286, an allosteric BRG1/BRM ATPase inhibitor, induced strong ferroptosis-associated changes, as indicated by the increased labile Fe²⁺ (Fig. 2i), ROS accumulation (Fig. 2j), and elevated lipid peroxidation (Fig. 2k). Importantly, FHD-286-induced cell death was rescuable by co-treatment with the ferroptosis inhibitor Fer-1 (Fig. 2l), confirming that its cytotoxicity is partly mediated through ferroptosis. Notably, depletion of BRM, another catalytic subunit of SWI/SNF chromatin remodeling complex, alone did not compromise MCL cell viability in dropout assays, indicating that the effects of FHD-286 in this context are primarily mediated through BRG1 inhibition (Supplementary Fig. 2h, i). Genetic depletion of BRG1 likewise promoted lipid peroxidation, further supporting the role of BRG1 in ferroptosis suppression (Supplementary Fig. 2j, k). Together, these findings support that aberrant BRG1 mediates BTKi resistance by transcriptionally suppressing ferroptosis.

### BRG1 regulates ferroptosis through both BTK-dependent and -independent pathways

**Fig. 3:**
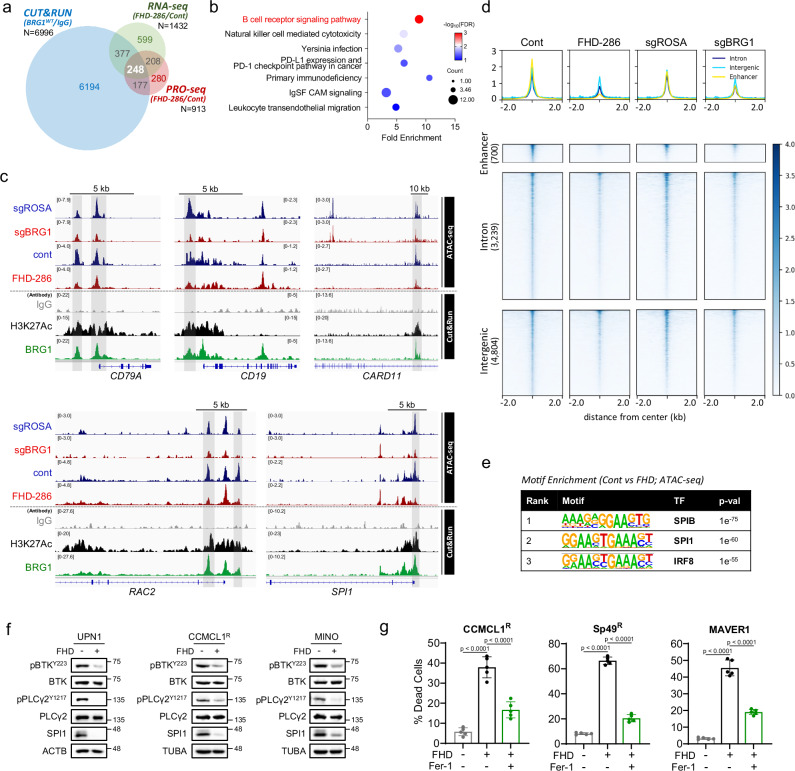
BRG1 regulates ferroptosis through both BTK-dependent and -independent pathways. **a** Venn diagram showing overlap between BRG1^WT^ and IgG Cut&Run peaks (FDR < 0.1), PRO-seq and RNA-seq DEGs (FHD-286 vs. control; FDR < 0.1, |FC| > 1.5) in MINO cells. **b** KEGG pathway enrichment of the overlapping genes from (**a**). **c** IGV tracks of ATAC-seq (sgROSA vs. sgBRG1 CCMCL1^Δp53^ cells; control vs. FHD-286 treated MINO cells) and IgG, H3K27ac, BRG1 Cut&Run (MINO^WT^ cells) at indicated loci. **d** Heatmap of ATAC-seq peak signals at regions with differential accessibility from MINO cells treated with vehicle or 10 nM FHD-286 for 24 h. For comparison, peaks from CCMCL1 cells acutely expressing sgROSA or sgBRG1 are also shown. Data are shown as normalized peak counts per million in a 2 kb window around peak center. **e** HOMER motif enrichment analysis of differentially accessible regions (MACS2, q < 0.0001) between control and FHD-treated MINO cells (cut-off: *p* val < 1e^−50^). **f** Immunoblot analysis of multiple MCL cells (UPN1, CCMCL1^R^, MINO) following FHD-286 treatment (10 nM, 48 h). **g** FHD-286 (10 nM, 96 h)-induced cell death response with or without Fer-1 in BTKi^R^ MCL cell lines (CCMCL1^R^, Sp49^R^, and MAVER1). Data represent mean ± s.d. from *n* = 5 biologically independent experiments. Statistical significance was determined using one-way ANOVA followed by Tukey’s test.

To uncover the BRG1-dependent transcriptional changes associated with ferroptosis resistance in MCL, we performed PRO-seq (Precision Run-On sequencing) and RNA-seq to capture both nascent RNA polymerase activity and steady-state transcriptional changes following FHD-286 treatment. PRO-seq and RNA-seq identified 913 and 1432 differentially transcribed genes, respectively (Fig. 3a; blue and green, Supplementary Data 3 and 4). These data were further integrated with BRG1^WT^ Cut&Run analysis, which defined 6996 genomic regions bound by BRG1 (Fig. 3a; red, Supplementary Data 5). Intersecting these datasets yielded 248 genes representing putative direct BRG1 targets, for which KEGG pathway analysis revealed B-cell receptor (BCR) signaling as a top enriched pathway (Fold enrichment = 8.84, FDR = 0.000363) (Fig. 3b). Consistently, Cut&Run analysis revealed BRG1 binding at regulatory regions of multiple BCR signaling genes, including *CD19*, *CD79A*, *RAC2*, *CARD11*, and *SPI1* (Fig. 3c). This was further supported by ATAC-seq analysis showing that these BRG1-bound sites largely overlap with accessible chromatin regions in sgROSA-transduced control cells but become less accessible following CRISPR-mediated BRG1 depletion (Fig. 3c). These findings suggest a regulatory role for BRG1 in BCR signaling, likely through direct binding and chromatin remodeling.

To further corroborate our findings, we next performed ATAC-seq in control and FHD-286-treated cells. Comparative analysis revealed a widespread loss of chromatin accessibility upon FHD-286-mediated BRG1 inhibition, predominantly at intergenic and intronic regions, with enhancer-associated peaks most prominently reduced (Fig. 3d). HOMER’s motif enrichment analysis further revealed that regions losing accessibility were enriched for Ets-family motifs, particularly those recognized by *SPI1* (PU.1) and *SPIB* (SpiB) (Fig. 3e), transcription factors essential for BCR signaling^42^. Consistent with our observation, pharmacological BRG1 inhibition in MCL cells markedly reduced BTK and PLCγ2 phosphorylation as well as SPI1 protein expression (Fig. 3f). Considering that BTK signaling protects MCL cells against ferroptosis (Supplementary Fig. 1e), these findings suggest that BRG1 regulates ferroptotic responses in MCL through transcriptional control of BCR signaling.

Beyond BCR signaling, our data suggest that BRG1 may contribute to ferroptosis regulation through additional pathway(s). Particularly, BTKi treatment of resistant MCL cells had minimal effects on ferroptosis induction or cell survival despite effective inhibition of BTK phosphorylation (Figs. 1f and 2b, c, Supplementary Fig. 2c). By contrast, BRG1 inhibition with FHD-286 triggered significant cell death in all three tested BTKi-resistant cell lines (CCMCL1^R^, Sp49^R^, and MAVER1), which was partially rescuable by co-treatment with the ferroptosis inhibitor Fer-1 (Fig. 3g). These results indicate that aberrant BRG1-mediated transcriptional programs can suppress ferroptosis independently of BTK signaling.

### MEF2B mediates ferroptosis response downstream of BRG1

**Fig. 4:**
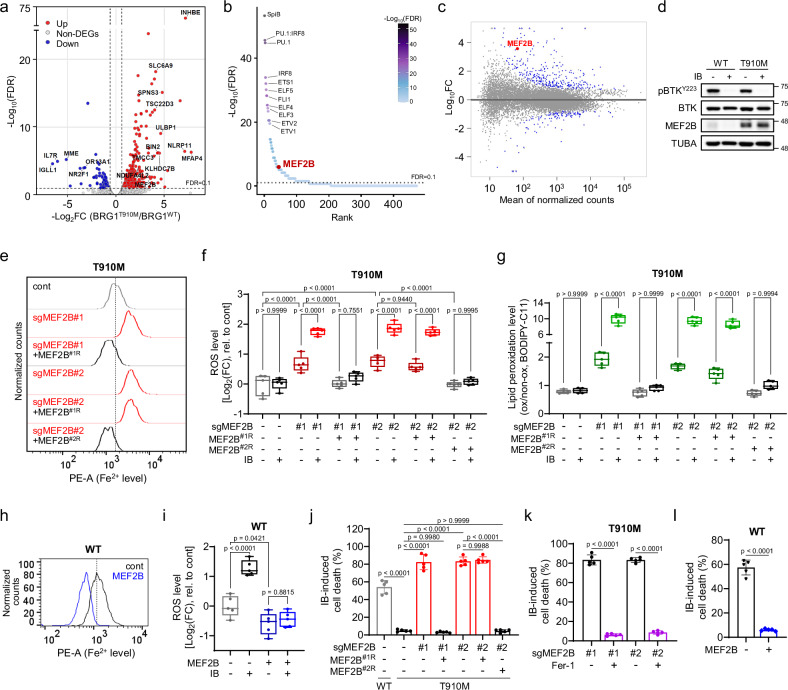
MEF2B mediates ferroptosis response downstream of BRG1. **a** Volcano plot of MINO BRG1^T910M^ vs. BRG1^WT^ RNA-seq DEGs (FDR < 0.1, |FC| > 1.5). **b** TF motif enrichment in differential peaks from Cut&Run data comparing MINO BRG1^T910M^ vs. BRG1^WT^. The 472 known TF binding motifs are ranked by significance (−Log_10_(FDR)). **c** MA plot of RNA-seq DEGs of MINO BRG1^T910M^ vs. BRG1^WT^ (blue dots, FDR < 0.1). The y-axis is Log_10_(FC), and the x-axis is the mean of normalized counts. **d** Immunoblot of JEKO1^WT^ and JEKO1^T910M^ cells following IB treatment (5 μM, 24 h). Basal Fe^2+^ (**e**), IB-induced ROS (**f**; 48 h), and IB-induced lipid peroxidation (**g**; 72 h) in MEF2B-depleted/rescued JEKO1^T910M^ cells. Representative histograms in (**e**) are from three independent experiments with similar results. Basal Fe^2+^ (**h**) and IB-induced ROS (**i**; 48 h) in MEF2B-overexpressing JEKO1^WT^ cells. **j** IB-induced cell death (96 h) in MEF2B-depleted and rescued JEKO1^T910M^ cells. **k** Effect of Fer-1 (2 μM) on IB-induced cell death in MEF2B-depleted JEKO1^T910M^ cells (96 h). **l** IB-induced cell death (96 h) in MEF2B-overexpressing JEKO1^WT^ cells. All IB treatments were performed at 5 μM. Quantitative data are from *n* = 5 biologically independent experiments. Bar plots represent mean ± s.d.; box plots in (**f**, **g**, **i**) indicate medians as center lines, 25th and 75th percentiles as box bounds, and minimum and maximum values as whiskers. Immunoblot in (**d**) is representative of experiments repeated independently three times with similar results. Statistical significance in (**l**) was determined using unpaired two-sided Student’s t-tests; all other comparisons were analyzed using one-way ANOVA followed by Tukey’s test.

To define aberrant BRG1-dependent transcriptional programs underlying BTKi resistance development in MCL, we next performed RNA-seq comparing BRG1^T910M^ and BRG1^WT^ MINO cells, which identified 438 differentially expressed genes (DEGs) (Fig. 4a, Supplementary Data 6). In parallel, Cut&Run profiling of epitope-tagged BRG1^T910M^ and BRG1^WT^ uncovered 1749 differentially occupied chromatin regions (Supplementary Fig. 3a, b, Supplementary Data 7 and 8). Approximately 23% of DEGs were associated with differential BRG1-bound regions (Supplementary Fig. 3c), supporting the contribution of BRG1 occupancy to transcriptional changes. As expected, genes linked to these BRG1-bound regions were enriched for B-cell lineage and fate-associated KEGG pathways (Supplementary Fig. 3d). Motif analysis of differential BRG1-bound regions further demonstrated enrichment of ETS and IRF transcription factor motifs, consistent with their established roles as BRG1-associated effectors^33,43^ (Fig. 4b). Notably, MEF2B motifs were also significantly enriched (Fig. 4b), and MEF2B itself was among the significantly upregulated DEGs in BRG1^T910M^ cells (Fig. 4c), suggesting convergence at both the chromatin and transcriptional levels. Consistent with these findings, MEF2B protein levels were markedly increased in both BRG1^T910M^ and BRG1^K785R^ cells relative to BRG1^WT^ controls (Supplementary Fig. 3e, f).

Interestingly, we observed that IB treatment reduced MEF2B protein expression only in wild-type but not mutant BRG1-expressing MINO and JEKO1 cells (Fig. 4d, Supplementary Fig. 4a), raising the possibility that aberrant BRG1-driven MEF2B upregulation contributes to ferroptosis suppression. Consistently, CRISPR-mediated MEF2B depletion significantly elevated basal labile Fe²⁺ levels in BRG1^T910M^ MINO and JEKO1 MCL cells (Fig. 4e, Supplementary Fig. 4b, c). Moreover, MEF2B depletion in BRG1^T910M^ cells also elevated the BTKi-induced ROS accumulation and lipid peroxidation, effects that were fully rescued by re-expression of a CRISPR-resistant MEF2B construct, ruling out off-target effects (Fig. 4f, g, Supplementary Fig. 4b, d–f). Consistently, ectopic MEF2B^WT^ expression in BRG1^WT^ cells reduced basal labile Fe^2+^ levels (Fig. 4h, Supplementary Fig. 4g, h) as well as baseline and BTKi-induced ROS levels (Fig. 4i, Supplementary Fig. 4g, i, j), indicating that MEF2B mediates BRG1-dependent suppression of ferroptosis.

Given these findings, we next investigated the role of MEF2B in BRG1-mediated BTKi resistance. As expected, MEF2B depletion sensitized BRG1^T910M^-expressing MCL cells to BTKi-induced cell death, an effect that was fully reversed upon CRISPR-resistant MEF2B re-expression (Fig. 4j, Supplementary Fig. 4b, k, l). Importantly, this sensitization was mitigated by co-treatment with the ferroptosis inhibitor Fer-1 (Fig. 4k, Supplementary Fig. 4m, n). Moreover, ectopic MEF2B expression in BRG1^WT^ cells conferred resistance to BTKi-induced cell death, further supporting its key role in BRG1-associated ferroptosis suppression (Fig. 4l, Supplementary Fig. 4o, p).

### NDUFA4L2 serves as the key downstream effector of MEF2B to mediate BTKi-induced ferroptosis resistance

**Fig. 5:**
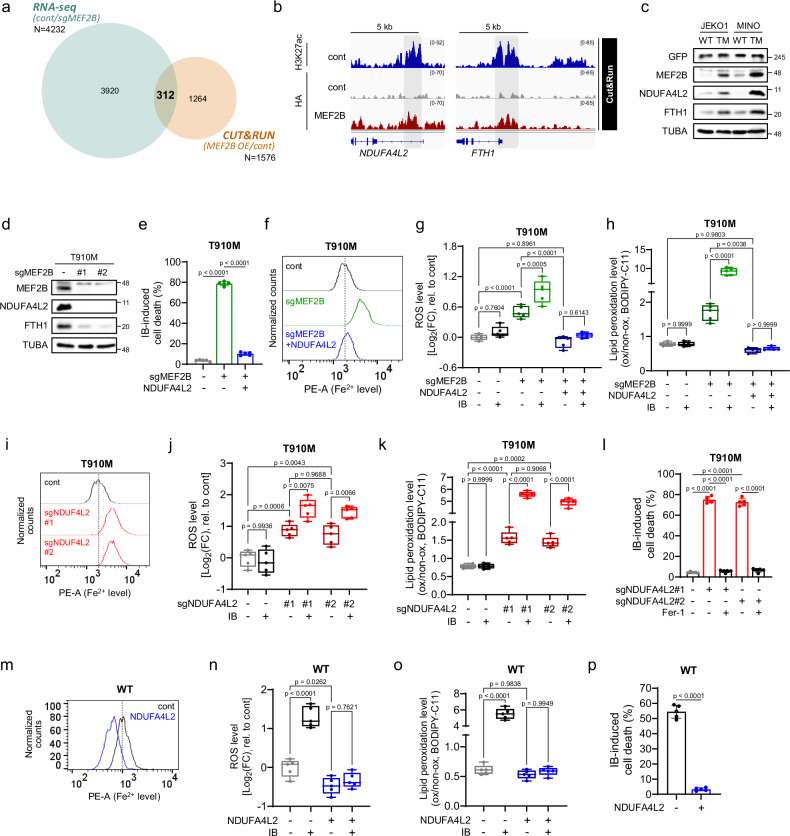
NDUFA4L2 serves as the key downstream effector of MEF2B to mediate BTKi-induced ferroptosis resistance. **a** Venn diagram showing overlap between control vs. sgMEF2B RNA-seq DEGs (FDR < 0.1, |FC| > 1.5), and HA-MEF2B Cut&Run peak (FDR < 0.1)-associated genes in JEKO1 cells. **b** IGV tracks of HA-MEF2B and H3K27ac Cut&Run in JEKO1 cells at the *NDUFA4L2* and *FTH1* loci. The H3K27ac track was retrieved from GSE141334. Immunoblot analysis of BRG1^WT^ vs. BRG1^T910M^ MCL cells (**c**) and MEF2B-depleted JEKO1^T910M^ MCL cells (**d**). **e** IB-induced cell death (96 h) in MEF2B-depleted JEKO1^T910M^ cells with or without NDUFA4L2 rescue. Basal Fe^2+^ (**f**), IB-induced ROS (**g**; 48 h), and IB-induced lipid peroxidation (**h**; 72 h) in MEF2B-depleted JEKO1^T910M^ cells with or without NDUFA4L2 reconstitution. Basal Fe^2+^ (**i**), IB-induced ROS (**j**; 48 h), and IB-induced lipid peroxidation (**k**; 72 h) in NDUFA4L2-depleted JEKO1^T910M^ cells. **l** Effect of Fer-1 (2 μM) on IB-induced cell death in NDUFA4L2-depleted JEKO1^T910M^ cells (96 h). Basal Fe^2+^ (**m**), IB-induced ROS (**n**; 48 h), and IB-induced lipid peroxidation (**o**; 72 h) in NDUFA4L2-overexpressing JEKO1^WT^ cells. **p** IB-induced cell death in NDUFA4L2-overexpressed JEKO1^WT^ cells (96 h). All IB treatments were performed at 5 μM. All quantitative data are from *n* = 5 biologically independent experiments. Bar plots represent mean ± s.d.; box plots in (**g**, **h**, **j**, **n**, **o**) indicate medians as center lines, 25th and 75th percentiles as box bounds, and minimum and maximum values as whiskers. Representative histograms in (**f**, **i**, **m**) and immunoblots in (**c**, **d**) are from experiments repeated independently three times with similar results. Statistical significance in (**p**) was determined using unpaired two-sided Student’s t-tests; all other comparisons were analyzed using one-way ANOVA followed by Tukey’s test.

MEF2B is a transcription factor crucial for normal B-cell development^44^. To identify downstream effectors of BRG1-MEF2B transcriptional program associated with ferroptosis resistance, we performed RNA-seq comparing control and MEF2B-depleted JEKO1 cells, identifying 4232 DEGs (Fig. 5a, green, Supplementary Data 9). Integration of this dataset with 1576 MEF2B Cut&Run peak-associated genes (Fig. 5a, orange, Supplementary Data 10) yielded 312 putative direct MEF2B targets. Functional prioritization highlighted genes linked to redox homeostasis, mitochondrial metabolism, and iron handling, including *GSTA4*^45^, *CBS*^46^, *NFE2L1*^47^, *HMGCS1*^48^, and *NDUFA4L2*^49,50^, with expression changes directionally consistent with the ferroptosis-resistant phenotype. Among these, NDUFA4L2 was the top-regulated gene in BRG1^T910M^ vs. BRG1^WT^ MINO RNA-seq (Fig. 4a, Supplementary Data 6). In addition, FTH1, a key regulator of cellular iron storage implicated in ferroptosis control^51^, was identified as a MEF2B-peak-associated gene (Fig. 5a), implying that iron-handling pathways may also be regulated by the BRG1-MEF2B transcriptional program. Supporting this, Cut&Run analysis further revealed the MEF2B occupancy at acetylated histone H3 lysine 27 (H3K27Ac)-positive regulatory regions of both *FTH1* and *NDUFA4L2* loci (Fig. 5b, Supplementary Fig. 5a). Consistently, BRG^T910M^-expressing cells showed markedly elevated protein levels of MEF2B, NDUFA4L2, and FTH1 (Fig. 5c). Notably, while MEF2B depletion reduced expression of both NDUFA4L2 and FTH1 (Fig. 5d, Supplementary Fig. 5b), NDUFA4L2 depletion did not affect MEF2B levels (Supplementary Fig. 5c), establishing MEF2B as an upstream regulator of both genes.

To determine whether MEF2B transcriptional activity is required for this regulation, we next expressed a DNA-binding-deficient MEF2B mutant (MEF2B^K23R^) that disrupts the conserved MADS domain^52^. As expected, MEF2B^K23R^ failed to activate a MEF2B-responsive reporter, whereas MEF2B^WT^ significantly induced the MEF reporter activity (Supplementary Fig. 5d). Moreover, expression of MEF2B^K23R^ in BRG1^T910M^ cells reduced NDUFA4L2 and FTH1 levels, consistent with dominant-negative inhibition of endogenous MEF2B activity (Supplementary Fig. 5e).

NDUFA4L2 (NADH dehydrogenase [ubiquinone] 1 alpha subcomplex, 4-like 2) is a mitochondrial protein. To assess its role in BRG1/MEF2B-mediated ferroptosis suppression, we ectopically expressed NDUFA4L2 in MEF2B-depleted BRG1^T910M^ cells (Supplementary Fig. 5f). As expected, NDUFA4L2 reconstitution restored resistance to IB, counteracting the sensitizing effects induced by MEF2B loss (Fig. 5e, Supplementary Fig. 5g). It also reversed the functional consequences of MEF2B depletion, restoring baseline and IB-induced increases in labile Fe²⁺, ROS, and lipid peroxidation to levels comparable with those observed in control BRG1^T910M^ cells (Fig. 5f–h, Supplementary Fig. 5h–j).

Consistently, depletion of NDUFA4L2 in BRG1^T910M^ MCL cells markedly elevated basal labile Fe²⁺, as well as both baseline and IB-induced ROS, and lipid peroxidation (Fig. 5i–k, Supplementary Fig. 5k–m). NDUFA4L2 depletion also sensitized the cells to IB-induced ferroptosis, an effect that was largely reversed by co-treatment with the ferroptosis inhibitor Fer-1 (Fig. 5l, Supplementary Fig. 5n). Conversely, ectopic overexpression of NDUFA4L2 in BRG1^WT^ MCL cells suppressed basal labile Fe²⁺, as well as both baseline and BTKi-induced ROS and lipid peroxidation, thereby reducing IB-induced cell death (Fig. 5m–p, Supplementary Fig. 5o–r). These findings together establish NDUFA4L2 as a key downstream effector of the BRG1-MEF2B axis that mediates ferroptosis resistance.

### NDUFA4L2 regulates mitochondrial activity to promote ferroptosis resistance

**Fig. 6:**
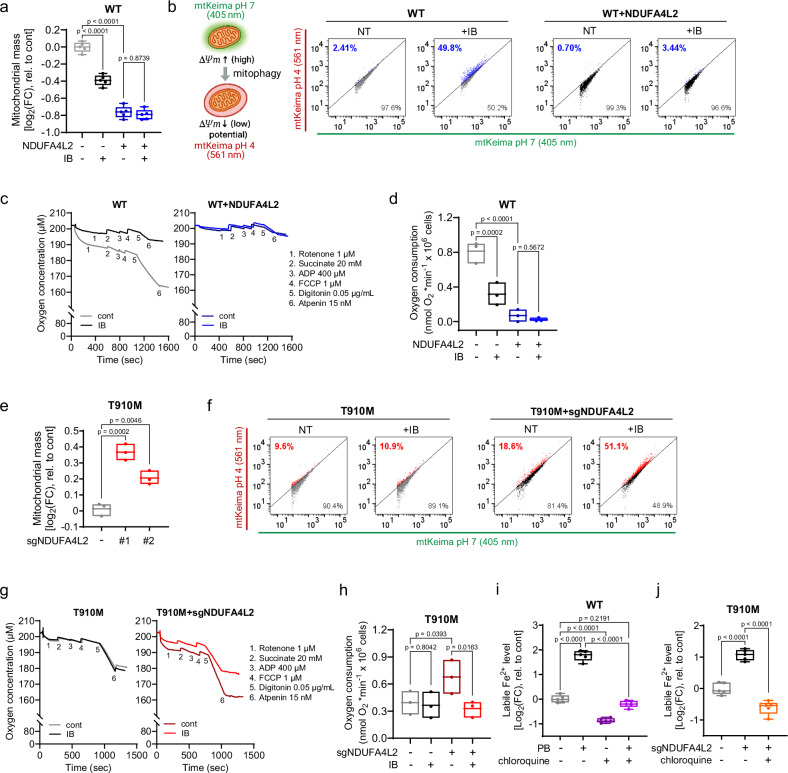
NDUFA4L2 regulates mitochondrial activity to promote ferroptosis resistance. Relative mitochondrial mass (**a**), mitophagy level (**b**; representative scatter plots), oxygen consumption trace (**c**), and total OCR (**d**) in control vs. NDUFA4L2-overexpressing JEKO1^WT^ cells with or without IB treatment (24 h). Relative mitochondrial mass (**e**), mitophagy levels (**f**; representative scatter plots), oxygen consumption traces (**g**), and total OCR (**h**) in control vs. NDUFA4L2-depleted JEKO1^T910M^ cells with or without IB treatment (24 h). Labile Fe^2+^ levels in: **i** JEKO1^WT^ cells treated with PB and/or chloroquine (50 μM), and **j** NDUFA4L2-depleted JEKO1^T910M^ cells treated with chloroquine (50 μM) (24 h). All IB and PB treatments were performed at 5 μM. Quantitative data are from *n* = 5 biologically independent experiments in (**a**, **i**, **j**) and *n* = 3 biologically independent experiments in (**d**, **e**, **h**). Box plots in (**a**, **i**, **j**) indicate medians as center lines, 25th and 75th percentiles as box bounds, and minimum and maximum values as whiskers. Floating bar plots in (**d**, **e**, **h**) indicate minimum and maximum values, with center lines denoting mean values. Representative scatter plots in (**b**, **f**) are from experiments repeated independently three times with similar results. Statistical significance in (**d**, **h**) was determined using one-way ANOVA followed by Fisher’s LSD multiple-comparison test; all other comparisons were analyzed using one-way ANOVA followed by Tukey’s test.

NDUFA4L2 mitigates oxidative stress by inhibiting mitochondrial complex I^50^. In BRG1^WT^ cells, ectopic expression of NDUFA4L2 suppressed mitochondrial biogenesis, turnover, and oxidative phosphorylation, as indicated by reduced mitochondrial mass (Fig. 6a, Supplementary Fig. 6a), decreased mitophagy measured by mt-Keima^53^ (Fig. 6b, Supplementary Fig. 6b), and a ~4.6-fold reduction in mitochondrial oxygen consumption rate (Fig. 6c, d). Conversely, NDUFA4L2 depletion in BRG1^T910M^ cells significantly increased mitochondrial mass, mitophagy, and oxygen consumption (Fig. 6e–h, Supplementary Fig. 6a, b), consistent with enhanced oxidative phosphorylation and increased turnover of damaged mitochondria.

Notably, BTK depletion reduced mitochondrial mass (Supplementary Fig. 6c), agreeing with previous reports that BTK inhibition induces mitochondrial dysfunction^54,55^. Consistently, IB treatment in BRG1^WT^ cells elevated ROS levels (Figs. 4i and 5n) and mitophagy (Fig. 6b, Supplementary Fig. 6b), while at the same time reducing mitochondrial respiration (Fig. 6c, d) and mitochondrial mass (Fig. 6a, Supplementary Fig. 6a). Importantly, these changes were accompanied by suppression of endogenous NDUFA4L2 protein expression (Supplementary Fig. 6d). Ectopic expression of NDUFA4L2 largely blocked these IB-induced changes (Figs. 5n and 6a–d, Supplementary Fig. 6a, b), supporting its protective role against mitochondrial dysfunction. Conversely, depletion of NDUFA4L2 in BRG1^T910M^ MCL cells, which are BTKi-resistant with elevated endogenous NDUFA4L2 expression, restored the BRG1^WT^-like response to IB treatment, enabling enhanced mitophagy (Fig. 6f, Supplementary Fig. 6b) and reduced oxygen consumption (Fig. 6g, h). These findings indicate that NDUFA4L2 protects MCL cells from IB-induced mitochondrial damage by reprogramming metabolism away from oxidative phosphorylation.

Ferroptosis is driven by labile iron that fuels redox cycling and iron-catalyzed lipid peroxidation and is tightly linked to mitochondrial metabolism^56^. Notably, PB-induced elevation of labile Fe²⁺ was blocked by co-treatment with the lysosomal inhibitor chloroquine (Fig. 6i, Supplementary Fig. 6e). Similarly, chloroquine completely suppressed NDUFA4L2 depletion-induced Fe²⁺ levels in BRG1^T910M^ cells (Fig. 6j, Supplementary Fig. 6f). These findings suggest that increased labile iron in these contexts involves lysosome-dependent processes, potentially including ferritinophagy^57^.

AMPK is a central metabolic sensor that has been implicated in the regulation of ferroptosis through modulation of lipid biosynthesis^58^. We asked whether altered mitochondrial respiration alone could account for NDUFA4L2-mediated ferroptosis resistance. Our findings instead support a model in which NDUFA4L2 enforces a preemptive metabolic state that limits BTKi-induced ferroptosis. By inhibiting mitochondrial Complex I, NDUFA4L2 reduces mitochondrial ROS output and establishes an AMPK-high, low-ROS state. Consistently, enforced NDUFA4L2 expression in BRG1^WT^ cells increased AMPK phosphorylation, whereas NDUFA4L2 depletion in BRG1^T910M^ cells suppressed basal AMPK activation (Supplementary Fig. 7a, b). Concordantly, MEF2B modulation altered NDUFA4L2 levels and AMPK phosphorylation (Supplementary Fig. 7c), placing AMPK downstream of the MEF2B-NDUFA4L2 axis. BTKi-resistant MCL cells similarly exhibited elevated basal AMPK activation compared with their matched sensitive counterparts (Supplementary Fig. 7d).

Functionally, pharmacologic AMPK activation with metformin suppressed BTKi-induced lipid peroxidation and ferroptosis (Supplementary Fig. 7e, f), whereas AMPK inhibition with BAY-3827 enhanced these processes in BRG1-mutant and BTKi-resistant MCL cells, respectively, recapitulating the effects of NDUFA4L2 overexpression and depletion (Supplementary Fig. 7g–j). Consistently, BAY-3827 sensitized BTKi-resistant primary MCL cells to IB, significantly increasing lipid peroxidation (Supplementary Fig. 7k–l). Together, these data support a model in which NDUFA4L2, downstream of the BRG1-MEF2B axis, suppresses ferroptosis by limiting labile iron accumulation and increasing the oxidative threshold for lipid peroxidation.

### BRG1-MEF2B-NDUFA4L2/FTH1 axis is associated with therapy resistance in MCL

**Fig. 7:**
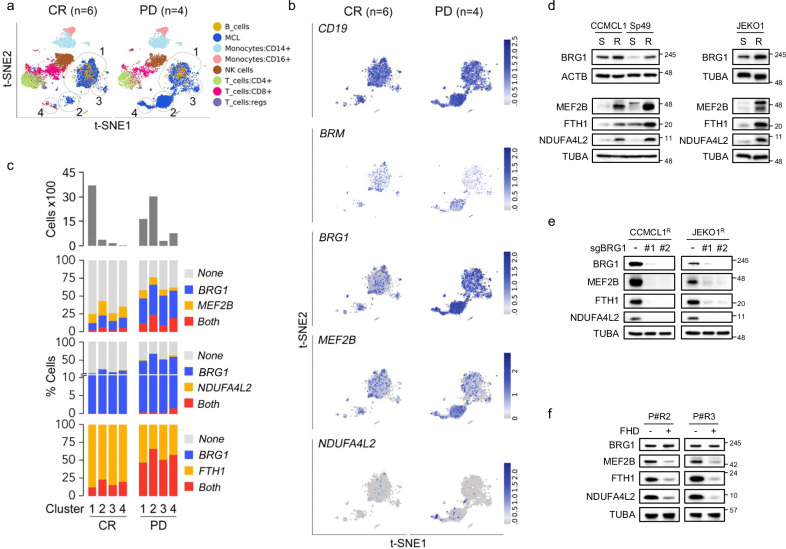
BRG1-MEF2B-NDUFA4L2/FTH1 axis is associated with therapy resistance in MCL. **a** *t*-distributed stochastic neighbor embedding (*t-*SNE) analysis of unsupervised clustering of single-cell RNA sequencing (scRNA-seq) of PBMCs from MCL patients with complete response (CR; *n* = 6 independent patient specimens) or progressive disease (PD; *n* = 4 independent patient specimens) in the PALIBR clinical study^86^. B cells (*CD19*^+^/CCND1^−^), MCL cells (*CD19*^+^/*CCND1*^+^), CD4^+^ T cells (*CD3D*^+^/*CD4*^+^), CD8^+^T cells (*CD3D*^+^/*CD8A*^+^), T-regs (*CD25*^+^/*FOXP3*^+^), natural killer (NK) cells (*KLRC1*^+^/*NCAM1*^+^/*GNLY*^+^), CD14^+^ (*CD14*^+^/*FCGR1A*^+^), and CD16^+^ (*CD16*^+^/*FCGR3A*^+^) monocytes are indicated. B cells and MCL cells comprise 4 major transcriptionally distinct clusters (gray circle). **b** t-SNE analysis of expression of indicated genes in MCL cells from samples as in (**a**). **c** Total number of MCL cells (top) and percentage of expressing cells (bottom) in each cluster of MCL cells. **d** Immunoblot comparing isogenic BTKi^S^ (S) and BTKi^R^ (R) pairs of CCMCL1, Sp49, and JEKO1 cells. **e** Immunoblot of BRG1-depleted CCMCL1^R^ and JEKO1^R^ cells (4-day post-sgRNA expression). **f** Immunoblot of IB-resistant MCL cultures following FHD-286 treatment (24 h). Immunoblots in (**d**–**f**) are representative of experiments repeated independently three times with similar results.

To assess the clinical relevance of the BRG1-driven transcriptional program identified above, we analyzed single-cell RNA-seq data from peripheral blood specimens of MCL patients who subsequently achieved a complete response (CR, 6 specimens) or had a progressive disease (PD, 4 specimens) in response to BTKi therapy (Fig. 7a). Across malignant MCL cells, BRG1 expression was significantly elevated in PD compared with CR samples (Fig. 7b, Supplementary Fig. 7m). Importantly, fraction of cells co-expressing BRG1 with MEF2B, NDUFA4L2, and FTH1 was elevated in PD samples (Fig. 7c), suggesting the activation of a coordinated transcriptional program in resistant disease. We subsequently validated the enrichment of these pathway effectors at the protein level in PD versus CR primary samples (Supplementary Fig. 7n). Consistent with these clinical observations, isogenic BTKi-resistant MCL cell lines also displayed upregulation of MEF2B, NDUFA4L2, and FTH1 compared with their parental BTKi-sensitive counterparts (Fig. 7d). Notably, both genetic depletion of BRG1 (Fig. 7e) and pharmacological inhibition with FHD-286 (Fig. 7f) led to marked downregulation of MEF2B, NDUFA4L2, and FTH1, supporting that these genes are coordinately regulated downstream of BRG1. These findings indicate that activation of the BRG1-MEF2B-NDUFA4L2 axis correlates with progressive disease following BTKi therapy, supporting further investigation of BRG1 as a potential therapeutic target.

### BRG1 inhibition re-sensitizes resistant MCL cells to BTKi

**Fig. 8:**
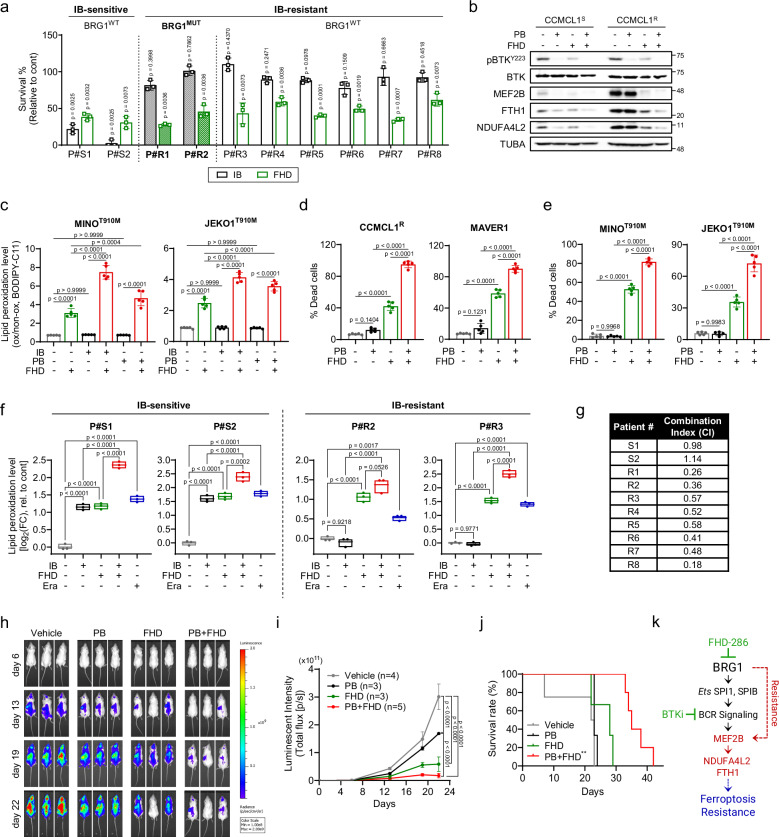
BRG1 inhibition re-sensitizes resistant MCL cells to BTKi. **a** Relative cell survival of primary MCL cultures from IB-responsive (*n* = 2 independent patient-derived cultures) or refractory (*n* = 8 independent patient-derived cultures) cases following treatment with IB or FHD-286 (96 h). Statistical significance was determined using unpaired two-sided Student’s t-tests with Holm-Šídák correction for multiple comparisons, relative to the corresponding untreated control for each patient sample. **b** Immunoblot of CCMCL1^S^ and ^R^ cells treated with PB, FHD-286, or their combinations for 24 h. The immunoblot is representative of experiments repeated independently three times with similar results. **c** Changes in lipid peroxidation (48 h) following IB, PB, FHD-286 treatment or their combination in MINO^T910M^, JEKO1^T910M^ cells. Cell death effect (72 h) of FHD-286 and PB combination in BTKi^R^ (CCMCL1^R^, MAVER1; **d**) and BRG1^T910M^ (MINO^T910M^, JEKO1^T910M^; **e**) MCL cells. **f** Changes in lipid peroxidation following IB, FHD-286 treatment, or their combination in primary MCL cultures for 48 h (*n* = 4 independent patient-derived cultures). **g** CI value for IB and FHD-286 in primary MCL cultures. **h** Representative bioluminescent images from CCMCL1^R^ CDX mice treated with PB (10 mg/kg; *n* = 3 mice), FHD-286 (1.2 mg/kg; *n* = 3 mice), or their combination (*n* = 5 mice). **i** Quantification of bioluminescent images from (**h**). Data represent mean ± s.e.m. **j** Kaplan-Meier survival curves of mice from (**h**, **i**). Statistical significance was determined using log-rank test (**p = 0.0014). **k** Schematic summary of demonstrated pathways in the study. All BTKi and FHD-286 treatment for in vitro and ex vivo experiments were performed at 5 μM and 30 nM, respectively. For quantitative in vitro cell-line panels, data represent mean ± s.d. from *n* = 5 biologically independent experiments in (**c**–**e**). Box plot in (**f**) indicates median as center line, 25th and 75th percentiles as box bounds, and minimum and maximum values as whiskers. For primary MCL cultures in (**a**, **f**), each culture was assayed in technical triplicate. Unless otherwise indicated, statistical significance was determined using one-way or two-way ANOVA followed by Tukey’s multiple-comparison test.

To evaluate the therapeutic potential of pharmacologic inhibition of BRG1, we tested FHD-286^59^. FHD-286 significantly suppressed the growth of all nine MCL cell lines tested, with IC₅₀ values uniformly in the nanomolar range, irrespective of their BTKi resistance status and BRG1 expression levels (Supplementary Fig. 8a–d). Consistently, FHD-286 exhibited robust cytotoxic effects in 10 primary cultures derived from IB-resistant MCL patients (Fig. 8a), highlighting its potential to overcome BTKi resistance.

While BRG1 regulates both BTK-dependent and -independent pathways, treatment of CCMCL1^S^ and CCMCL1^R^ cells with FHD-286 incompletely suppressed BTK activity (Fig. 8b), which may explain its limited single-agent efficacy. This prompted us to evaluate whether combining FHD-286 with BTKi could better suppress BTK signaling and enhance therapeutic efficacy. Remarkably, combined treatment of FHD-286 with PB, a reversible BTKi with improved target coverage and pharmacokinetics^60^, not only fully inhibited the BTK signaling but also enhanced suppression of the MEF2B-NDUFA4L2/FTH1 signaling axis in BTKi-resistant CCMCL1 as well as MINO^T910M^ and JEKO1^T910M^ cells (Fig. 8b, Supplementary Fig. 9a). Consequently, the combination treatment-induced robust ferroptosis, shown by elevated ROS production, lipid peroxidation, and markedly enhanced cell death (Fig. 8c–e, Supplementary Fig. 9b–e). A competition-based proliferation assay further demonstrated that PB treatment accelerates the selective elimination of BRG1-depleted cells (Supplementary Fig. 9f). Notably, this effect was also recapitulated in primary MCL samples, where combined FHD-286 and IB treatment-induced marked lipid peroxidation (Fig. 8f) and significantly increased cell death (Supplementary Fig. 9g), compared with single-agent treatments. Combination index values ranging from 0.18 to 0.58 further revealed strong synergistic interactions between FHD-286 and IB in IB-resistant primary MCL cultures (Fig. 8g), suggesting that BRG1 inhibition re-sensitizes BTKi-resistant MCL cells to BTK inhibition.

We next assessed the therapeutic potential of this combination in vivo using a CCMCL1^R^ cell-derived xenograft (CDX) model (Fig. 8h). Bioluminescence imaging revealed that FHD-286 monotherapy produced moderate tumor growth suppression, and PB alone elicited minimal effect compared with vehicle controls. By contrast, combined FHD-286/PB treatment produced pronounced anti-tumor activity (Fig. 8i). Immunohistochemical analysis of tumor tissues further revealed suppression of BCR signaling, reflected by reduced SPI1 expression, along with decreased NDUFA4L2 levels and increased lipid peroxidation marker 4-HNE, with these changes most pronounced in tumors from the combination treatment group (Supplementary Fig. 10a, b). In contrast, cleaved caspase-3 staining showed a marginal increase (~3% positive area), suggesting against apoptotic response. Consistent with these results, survival analysis revealed little to no benefits with PB monotherapy and modest improvement with FHD-286 (median survival extended from 22.5 to 28 days), while the FHD-286/PB combination markedly prolonged survival to a median of 35 days (Fig. 8j). Collectively, these findings indicate that combined BRG1 and BTK inhibition effectively suppresses the MEF2B-NDUFA4L2/FTH1 axis and enhances ferroptosis sensitivity in BTKi-resistant MCL (Fig. 8k), providing a preclinical rationale for dual BTKi and BRG1 inhibition as a therapeutic strategy in refractory MCL.

## Discussion

Dynamic transcriptional and metabolic rewiring is a key driver of therapy resistance. Here, we identify aberrant BRG1-driven ferroptosis resistance mediated through MEF2B-NDUFA4L2/FTH1 expression. As a key mediator of resistance, MEF2B directly induces the transcription of NDUFA4L2 and FTH1, suppressing mitochondrial metabolism and iron levels against BTKi-induced ferroptosis. Through inhibition of mitochondrial Complex I, NDUFA4L2 establishes a low-ROS, AMPK-activated metabolic state that preemptively raises the threshold for ferroptosis induction. We further uncovered that BRG1 inhibition suppresses MEF2B-NDUFA4L2/FTH1 gene expression network, thereby promoting ferroptosis. Accordingly, combined BRG1 and BTK inhibition synergistically suppresses MCL progression, supporting BRG1 targeting as a strategy to overcome BTKi resistance. These findings reveal a key role for BRG1 in regulating ferroptosis susceptibility, establishing a mechanistic link between epigenetic dysregulation and BTKi resistance.

Importantly, our findings primarily address mechanisms of acquired BTKi resistance rather than baseline sensitivity. Induced BTKi-resistant models were used to interrogate adaptive chromatin and transcriptional programs that emerge under sustained BTKi exposure. In this context, the BRG1^T910M^ allele serves as a functional model of deregulated BRG1 activity rather than a surrogate for primary mutation-driven resistance. BRG1 does not act only through mutation or expression level, but also through context-specific transcriptional reprogramming. The MEF2B-driven transcription, therefore, represents BRG1-driven ferroptosis suppression under BTKi pressure and identifies a subset of resistant MCL. Consistent with this framework, BRG1 expression and pathway activity are increased in BTKi-resistant cells relative to isogenic sensitive counterparts and are elevated in patient samples at progression compared with complete response, supporting a role for adaptive BRG1 upregulation in reinforcing ferroptosis suppression during BTKi resistance.

Aggressive MCLs show elevated iron metabolism^61,62^, rendering them more vulnerable to ferroptotic stress. Recent studies have demonstrated that BTK inhibition can engage ferroptosis in B-cell lymphomas, particularly in combination settings, including BTKi with BCL-2 inhibition or iron-targeting agents^63,64^, and through direct modulation of antioxidant defense mechanisms^65^. While BTKi are known to induce apoptosis primarily, these studies collectively establish ferroptosis as a complementary, clinically relevant mode of BTKi-associated cytotoxicity. Induction of ferroptosis was consistently observed across multiple BTKi with distinct modes of target engagement (Fig. 1b–d) and was reproduced by BTK depletion (Supplementary Fig. 1b–e), indicating an on-target effect. Notably, ferroptosis-associated changes were abrogated in MCL cells under an aberrant BRG1-driven transcription program (Fig. 2d–f, Supplementary Fig. 2d), but were restored by FHD-286 treatment (Fig. 2i–k). These findings show that BRG1 mediates BTKi resistance by suppressing ferroptosis, which is a key vulnerability of MCL.

MEF2B is a transcription factor implicated in GC-derived lymphomas, where it regulates BCL6 and is frequently mutated to enhance oncogenic activity^44,66^. In MCL, MEF2B mutations are less common (~3–9%)^67,68^, and their oncogenic role remains largely uncharacterized. Our study identifies MEF2B as a previously unrecognized effector of ferroptosis resistance, upregulated by aberrant BRG1 activity (Fig. 4, Supplementary Figs. 3 and 4). Unlike the MYC-driven oxidative phosphorylation program, where MEF2B cooperates with MYC and DNMT3A to promote IB resistance by enhancing oxidative phosphorylation^69^, BRG1 mutations induce MEF2B expression in a distinct regulatory context that bypasses MYC and reprograms resistance through ferroptosis suppression. These findings highlight MEF2B as a converging node in BTKi resistance, enabling engagement of mechanistically distinct survival pathways.

NDUFA4L2 is transcriptionally upregulated by HIF1α under hypoxia or oxidative stress, where it facilitates metabolic adaptation by limiting ROS production. This mitochondrial protein reduces oxidative phosphorylation and oxygen consumption by inhibiting mitochondrial Complex I activity^50,70^. We show that MEF2B directly regulates NDUFA4L2, linking transcriptional control to mitochondrial respiration (Fig. 5). By restraining oxygen consumption, NDUFA4L2 protects cells from BTKi-caused mitochondrial damage (Fig. 6a–h, Supplementary Fig. 6a, b). This metabolic reprogramming is coupled to increased basal AMPK activation, which further suppresses lipid peroxidation and ferroptotic cell death. This metabolic shift restricts the pool of BTKi-induced dysfunctional mitochondria, thereby reducing iron release mediated by ROS-driven ferritinophagy^57^. In parallel, iron availability for lipid peroxidation is further restricted by upregulated FTH1, which is induced by MEF2B (Fig. 5b, d, Supplementary Fig. 5a, b). Together, this BRG1-MEF2B-NDUFA4L2/FTH1 axis represents a previously unappreciated transcriptional mechanism by which lymphoma cells actively suppress BTKi-associated ferroptosis, extending beyond the mitochondrial and antioxidant pathways described in prior studies^63–65^.

Mutant BRG1 promotes BTKi resistance through activation of the MEF2B-NDUFA4L2/ FTH1 axis, yet we also identify BRG1 as an essential gene, as its complete loss compromises MCL cell viability. Mechanistically, BRG1 sustains pro-survival BCR signaling by maintaining widespread chromatin accessibility at Ets transcription factor binding sites, particularly those of SPI1 and SPIB (Fig. 3e). This dual role of BRG1 partially aligns with the findings from Deng et al.^33^, which described BRG1 as a haploinsufficient tumor suppressor in GC-derived lymphomas, fine-tuning centrocyte fate through regulating SPI1, IRF family members, and NFκB. In their model, complete BRG1 loss impaired GC formation, whereas heterozygosity facilitated lymphomagenesis. Thus, BRG1 emerges as both a lineage-survival factor and a mediator of therapy resistance, underscoring its potential as a context-dependent, targetable vulnerability in MCL.

Among strategies to overcome BTKi resistance, BCL-2 targeted regimens have shown favorable efficacy^38,71^, potentially due to simultaneous engagement of apoptotic and ferroptotic cell death programs. Consistent with reports that BTKi combinations induce ferroptosis through multiple mechanisms^63–65^, our data indicate that resistance arises when ferroptosis is selectively suppressed by epigenetic reprogramming. In this context, sustained AMPK activation downstream of NDUFA4L2 imposes an additional metabolic barrier to ferroptosis induction, highlighting the need for precision strategies tailored to the dominant resistance mechanism.

Consistent with the prior report that ARID1A-mutant lymphomas exhibit enhanced sensitivity to BAF complex inhibition^43^, our study demonstrates that BRG1-mutant MCL cells are more susceptible to FHD-286, supporting a synthetic lethal interaction in BTKi-refractory disease. Mechanistically, FHD-286 inhibits both SPI1/SPIB-driven BCR signaling and MEF2B expression, collectively promoting ferroptosis (Fig. 8k). These findings provide a strong mechanistic rationale for dual targeting of BRG1 and BTK, which resulted in significant synergistic cytotoxicity across multiple preclinical MCL models (Fig. 8, Supplementary Fig. 9d–f). Altogether, targeting BRG1-dependent resistance to ferroptosis represents a promising therapeutic strategy for BTKi-refractory MCL.

## Methods

All animal studies were approved by the Institutional Animal Care and Use Committee (IACUC) of Weill Cornell Medicine under protocol number 2018-0039 and complied with all relevant ethical regulations. Peripheral blood from MCL patients was obtained following written informed consent under a protocol approved by the Institutional Review Board of The Ohio State University in accordance with the Declaration of Helsinki, and all samples were fully de-identified prior to analysis. Information about the antibodies, chemicals, and biologicals, cell lines, oligonucleotides, recombinant DNA, software, algorithms, and instruments is included under Supplementary Table 1.

### Measuring cellular ROS, labile iron, or lipid peroxidation levels

CellROX™ Deep Red Reagent (Thermo Fisher Scientific, C10422) was used at 0.5 μM for 1 h incubation in growth media to detect oxidative stress and ROS levels. Oxidized probe fluorescence was measured by flow cytometry at excitation/emission (ex/em) maxima of ~644/665 nm. Labile iron levels were assessed using the FerroOrange fluorescence probe (Cell Signaling Technology, 36104S). Cells were harvested, washed once with HBSS, and incubated with 1 μM probe for 30 min at 37 °C, followed by measurement of fluorescence intensity at ex/em maxima of ~540/580 nm. Intracellular lipid peroxidation was evaluated using BODIPY-C11 (Cayman Chemical, #27086). Cells were incubated at 5 μM probe in growth medium for 1 h, and fluorescence was detected by flow cytometry at ex/em maxima of 581/591 nm (non-oxidized) and 488/510 nm (oxidized). The ratio of oxidized to non-oxidized signal was calculated to quantify lipid peroxidation levels.

### Primary MCL ex vivo treatment

Peripheral blood from IB-resistant, relapse MCL patients was obtained following written informed consent under a protocol approved by the Institutional Review Board of The Ohio State University in accordance with the Declaration of Helsinki. MCL cells were isolated using CD19 magnetic beads, and purity was determined by flow cytometry analysis using CD45, CD5, and CD20 staining. Primary MCL cells with TP53 mutations were cultured in RPMI 1640 medium supplemented with 20% FBS, 50 U/mL penicillin-streptomycin, Glutamax, IL-6 (40 U/mL), soluble IL-6R (40 U/mL), IL-10 (50 ng/mL), IGF-1 (30 ng/mL), sCD40L (0.5 μg/mL), and BAFF (50 ng/mL). A round-bottom 96-well plate with 150 μL of 2 × 10^6^/mL cells was treated with drugs for 72–96 h, and the number of live cells was determined.

### Cell lines and culture details

MCL cell lines CCMCL1, JEKO1, UPN1, MAVER1, MINO, Z138, and REC1 were cultured in RPMI1640 supplemented with 10% heat-inactivated fetal bovine serum (FBS), 2 mM L-glutamine, and 50 units/mL penicillin-streptomycin in a 37 °C humidified 5% CO2 incubator. All cell lines used for gene knockdown were lentivirally transduced to express human codon-optimized SpCas9 nuclease for the establishment of somatic deletion cells by CRISPR/Cas9 genome editing. Infected cells were selected with blasticidin or puromycin for 7–10 days. BTKi-resistant CCMCL1^R^ and Sp49^R^ cell lines were generated as previously described^21^, by maintaining them in gradually increasing concentrations of BTKi (i.e., 10 μM IB, ZB, or PB) over 8 weeks. The BRG1^WT^ and BRG1^T910M^-expressing paired isogenic cell lines were generated using the PiggyBac transposon system delivered by electroporation for stable genomic integration, as previously described^72^. Both pPB CAG SMARCA4-Venus (Addgene plasmid # 153950) and pPB CAG SMARCA4 T910M-Venus (Addgene plasmid # 153951) were gifts from Luca Tiberi (http://n2t.net/addgene:153950 and http://n2t.net/addgene:153951; RRID: Addgene_153950 and RRID: Addgene_153951). All cell lines were repeatedly tested for mycoplasma contamination by PCR.

### sgRNA and expression constructs

All the sgRNAs were cloned into BsmB1-digested LRG2.1 or lenticrisprV2 vectors. pXPR_016-sgATF4-1 was a gift from William Hahn (Addgene plasmid # 202455; http://n2t.net/addgene:202455; RRID:Addgene_202455). For the ectopic expression of HA-MEF2B, a full-length MEF2B with N-terminal HA tag was amplified using CloneAmp™ HiFi PCR Premix and cloned into pLU vector by Gibson assembly.

### Generation of viral particles

Viruses were produced by co-transfection of indicated plasmids and packaging vectors into HEK293T packaging cells as previously described^73^. In brief, 8.0 × 10^6^ HEK293T cells in 100 mm tissue culture dishes were transfected with 8.5 μg of each plasmid DNA along with 4 μg of pMD2.G and 6 μg of psPAX2 packaging vectors using polyethylenimine. The media was replaced 6–8 h post-transfection. The virus-containing supernatant was collected at 48 and 72 h post-transfection, pooled, and filtered through 0.4 micron PES membranes.

### sgRNA pooled library negative selection screening

A specialized library targeting 455 human chromatin-modifying factors was manually assembled for this study. To ensure robust gene disruption, we designed 4–14 sgRNAs per gene, prioritizing the targeting of functional protein domains as defined by the NCBI Conserved Domains Database. Candidates were filtered to maximize on-target efficiency and minimize off-target effects according to established computational models. The resulting oligonucleotide pool, which included both positive and negative control sequences, was synthesized by CustomArray Inc, PCR amplified, and cloned into the BsmB1 site of the LRPuro lentiviral vector. The composition and uniformity of the final plasmid pool were validated via deep sequencing.

The sgRNA pooled library negative selection screening^41^ was performed to identify epigenetic dependencies in MCL. Cas9-stable cell lines were transduced with the lentiviral library at a multiplicity of infection (MOI) of approximately 0.4 to favor single-copy integration. Throughout the screen, we maintained a minimum population of 10^8^ cells to ensure sufficient library coverage. We established the baseline library distribution by harvesting a subset of 10^8^ cells at 3 days post-infection. The remaining cells were maintained in culture for 20 population doublings to allow for the depletion of cells harboring sgRNAs that target essential fitness genes. At the screen’s conclusion, genomic DNA was isolated through overnight digestion at 56 °C in a proteinase K-enriched lysis buffer, followed by triple phenol-chloroform extraction and ethanol precipitation. To recover the integrated sgRNA sequences, we performed a nested PCR strategy. The initial amplification utilized Takara Taq to isolate a ~250 bp fragment, which was subsequently purified via gel electrophoresis. Finally, 20 ng of this primary product served as a template for a secondary PCR using Q5 High-Fidelity 2× Master Mix (New England Biolabs) to ligate Illumina-compatible barcodes for next-generation sequencing and subsequent depletion analysis.

### Competition-based proliferation assays (GFP dropout assay)

Cas9-expressing cell lines were infected with sgRNAs linked with GFP using the U6-sgRNA-GFP (LRG2.1) plasmid. For dropout assay, 0.25 × 10^6^ cells were transduced with lentivirus encoding sgRNA. After 4 days, flow cytometry analysis was performed, and percentage of GFP-positive cell population was determined by flow cytometer (BD FACSymphony™) time-dependently.

### Immunoblot analysis

Cells were lysed for 30 min at ice in cell lysis buffer [20 mM Tris-HCl (pH 7.5), 150 mM NaCl, 1 mM EDTA, 1 mM EGTA, 1% Triton X-100, and 2.5 mM sodium pyrophosphate] containing protease inhibitor cocktails. Protein concentration was determined by using Pierce^TM^ BCA protein assay kit. 10–20 μg of proteins were separated by SDS-PAGE electrophoresis and transferred to Nitrocellulose/PVDF membrane, which was blocked by incubating for 1 h at room temperature (RT) in 5% non-fat dried milk in TBST buffer. Membranes were incubated with primary antibodies diluted in 5% BSA in TBS. For GFP, BRG1, phospho-BTK (Y223), BTK, vinculin, MEF2B, TUBA, NDUFA4L2, FTH1, HA, phospho-PLCγ2 (Y1217), PLCγ2, SPI1, and ACTB overnight at 4 °C and then with HRP-conjugated anti-mouse or anti-rabbit IgG for 1 h at RT. Blots were developed with the SuperSignal™ West Pico/Femto Chemiluminescent substrate. Further information about the usage of antibodies is included in the Supplementary Table 1.

### Pirtobrutinib and FHD-286 treatment of MCL xenografts

BTKi-resistant CCMCL1^R^ cells were lentivirally transduced to express firefly luciferase. Two million cells were tail vein injected into NSG (NOD.Cg-PrkdcscidIl2rgtm1Wjl/SzJ) mice. Mice were group-housed (up to five animals per cage) in individually ventilated cages with ad libitum access to food and acidified water (pH 2.5–2.8) in a temperature-controlled (22.2 ± 0.5 °C) and humidity-controlled (30–70%) facility maintained on a 12-h light/12-h dark cycle. Following engraftment, mice were randomized into four groups: vehicle (1% DMSO in 20% 2-hydroxypropyl-β-cyclodextrin), pirtobrutinib (10 mg/kg, once daily), FHD-286 (1.2 mg/kg, once daily), or combination therapy. Treatments were done by i.p. injection for a 3–5 week period. Tumor growth was monitored via weekly bioluminescent imaging with an IVIS Spectrum system (Caliper Life Sciences). Mice were humanely euthanized upon reaching institutional health endpoints, and these maximal burdens were not exceeded.

### Immunohistochemistry of tumor sections

Tumor-bearing spleen tissues harvested from mice treated with vehicle control, Pirtobrutinib (PB), FHD-286, or the combination were fixed in 10% neutral buffered formalin and embedded in paraffin. Sections (5 μm thickness) were deparaffinized, rehydrated, and subjected to heat-induced antigen retrieval. To assess metabolic reprogramming, apoptosis, and lineage factors, sections were incubated with primary antibodies against NDUFA4L2 (1:200), cleaved caspase-3 (1:400), and SPI1 (1:500).

To evaluate ferroptosis induction, lipid peroxidation was quantified via immunohistochemical staining for 4-hydroxy-2-nonenal (4-HNE; mouse, 1:500). Primary antibodies were detected using Vector Elite ABC kit and visualized with 3,3′-Diaminobenzidine (DAB), resulting in a brown signal. 4-HNE staining was utilized as a specific marker for lipid peroxidation-derived aldehydes. Sections were counterstained with hematoxylin, dehydrated, and imaged. IHC optical density (OD) scores were quantified using the ImageJ IHC Profiler plugin and calculated according to the published scoring method^74^. OD values were normalized to the mean of the control group and log₂-transformed for visualization (log₂ relative OD). Cleaved caspase-3 staining was quantified as the percentage of positive tumor cells.

### Cytotoxic killing analysis and viability assay

For cytotoxic killing analysis, 5.0 × 10^4^ MCL cells in 2 mL were treated with compounds. After 4 days, the fraction of dead cells was determined by FACS analysis for % of ToPro3-staining positive cells. For IC_50_ of FHD-286, cell viability was assessed using the XTT assay. Briefly, 1 × 10⁴ cells/well were seeded in 96-well plates and treated with the indicated doses for triplicate. After 96 h, XTT labeling reagent mixed with electron coupling reagent was added directly to each well and incubated 3 h at 37 °C. Formation of formazan dye was quantified by absorbance at 450 nm with a reference wavelength of 650 nm using a microplate reader.

### Mitophagy and mitochondria mass measurement

Mitophagy was measured using mt-Keima ratiometric probe. pHAGE-mt-mKeima was a gift from Richard Youle (Addgene plasmid # 131626; http://n2t.net/addgene:131626; RRID:Addgene_131626). Cells were lentivirally infected to express mt-mKeima. Mitophagy levels were determined by acquiring intensity of emission spectra using BV605 (violet laser 405 nm) together with PE-CF594 (yellow laser 565 nm) and emission spectra with 610/20 BP. MitoTracker® Deep Red FM (Thermo Fisher Scientific) probe was used to measure mitochondrial mass. Cells were harvested, washed with PBS, and incubated with 50 nM MitoTracker® Deep Red FM in pre-warmed serum-free medium at 37 °C for 30 min. Flow cytometry was performed to determine the mean fluorescence intensity (MFI), which was used as a proxy for mitochondrial mass.

### Mitochondrial respiration measurement

Oxygen consumption was measured using a high-resolution respirometer (O2K oxygraph, Oroboros Instruments, Innsbruck, Austria) at 37 °C. Briefly, cells were harvested at 500 g for 5 min and washed twice with Hank’s solution to remove glucose-containing culture media. Finally, cells were resuspended in 50 ml of Hank’s solution (5 × 10^6^ cells/mL) and added directly to the Oroboros chamber containing 2 mL of MIR05 buffer, supplemented with 4 mM KH_2_PO_4_. After registering the basal oxygen consumption, sequential additions of the following compounds were made: 1 μM rotenone, 20 mM succinate, 400 μM ADP, 1 μM FCCP, 0.05 μg/mL digitonin, and 15 nM atpenin A5. Respiration rates data were recorded using the DataLab software (version 6.1.0.7) at 1 Hz time resolution and analyzed in Microcal Origin. All results are expressed as means ± SD for at least three biological replicates.

### Calculation of combination index (CI)

Combination index (CI) values were calculated using CompuSyn software (ComboSyn Inc.) based on the Chou–Talalay method, using cell viability percentages from ex vivo MCL primary cell cultures treated with FHD-286, IB, or their combination. CI < 1, = 1, and > 1 indicated synergism, additivity, and antagonism, respectively.

### Cleavage under targets and release using nuclease (Cut&Run) analysis

50,000 MCL cells were harvested and fixed with 0.1% formaldehyde for 5 min and further processed by Epicypher ChIC system using validated antibodies. All the recovered DNA was used for dual-indexed library preparation and sequenced at Weill Cornell Genomics Core. Reads were trimmed and quality filtered using TrimGalore v0.4.5 with cutadapt v1.15 and FastQC v0.11.5, then aligned to the human genome (hg38) using bowtie2 v2.3.4.1; duplicates were removed with Picard MarkDuplicates v2.16.0. Enriched regions were identified with MACS2 (v2.2.9.1, *p* = 0.001) using matched IgG controls, and normalized read density profiles were generated with BEDTools v2.29.2, and tracks were visualized using Integrative Genomics Viewer (IGV) v2.17.2. A global peak atlas was constructed by merging peaks within 500 bp and quantifying reads using featureCounts v1.6.1; counts were normalized by sequencing depth, and differential enrichment was calculated with DESeq2 v1.46.0. Peaks were annotated by assigning intragenic peaks to their respective genes and intergenic peaks to the nearest transcription start site. Transcription factor motif enrichment analysis was performed on differential BRG1 binding sites using HOMER (v4.11) findMotifsGenome.pl. Analysis was executed with a genomic background and the following parameters: -size 200, -len 8,10, and -mask. Enriched motifs were ranked by statistical significance based on the Benjamini-Hochberg adjusted q-value (−log_10_).

### ATAC-seq data processing and analysis

ATAC-seq was performed at the Cornell University BRC Epigenomics Facility (RRID:SCR_021287). Briefly, 55,000 cells flash-frozen in 10% DMSO were lysed, permeabilized, and tagmented using the Omni ATAC-seq protocol^75^ followed by 12 cycles of barcoding PCR. Gel-purified libraries were sequenced on the Element Biosciences AVITI to obtain approximately 10 M paired-end reads. Paired-end ATAC-seq reads were aligned to the hg38 reference genome using Bowtie2 (v2.5.2) in local alignment mode. Reads were position-sorted and indexed using SAMtools (v1.19.1). PCR duplicates were removed using Picard MarkDuplicates (v3.1.1) with duplicate removal enabled, and alignment quality was assessed using SAMtools flagstat and Picard insert size metrics. Genome-wide accessibility tracks were generated using deepTools (bamCoverage). Only uniquely mapped reads with mapping quality ≥10 were retained, and mitochondrial reads were excluded. Read coverage was normalized by scaling to 10 million unique fragments per sample, and bigWig files were generated using a bin size of 25 bp without additional normalization. Aggregate chromatin accessibility profiles across gene bodies were computed using deepTools (v3.5.6) computeMatrix (scale-regions mode) with regions extended ±3 kb from transcription start and end sites, and visualized using plotHeatmap. Accessible chromatin regions were identified using MACS2 (v2.2.9.1) with parameters --nomodel and a *p*-value threshold of 0.001, using a common input control BAM file for background correction. For differential accessibility analysis, read counts over peak regions were quantified using featureCounts (Subread v2.0.6). Peaks with low counts (total count <10 across all samples) were excluded. Differential analysis was performed using DESeq2 (v1.44.0) with a design formula of ~condition, and size-factor normalization was applied. Statistical significance was assessed using Wald tests, and peaks with an adjusted *p* value ≤ 0.1 were considered significant.

### RNA-seq and pathway enrichment analysis

Total RNAs were isolated from cells using NucleoSpin RNA kit and subjected to RNA sequencing at the Genomics Resources Core facility of Weill Cornell Medicine. RNA-seq libraries were prepared using the Illumina TruSeq stranded mRNA library preparation kit. Paired-end FASTQ files underwent quality trimming with bbduk (ktrim = r k = 23 mink = 11 hdist = 1 qtrim = rl trimq = 10 maq = 10) to remove adapters (TruSeq, polyA) and low-quality bases, followed by FastQC QC (v0.12.1). Trimmed reads were aligned to GRCh38/hg38 reference genomes using STAR 2.7.11b (2-pass Basic mode). FeatureCounts (Subread v2.0.6) were generated for reverse-stranded exon-level gene counts (-p -t exon -g gene_id -s 2), with BAMs indexed via samtools 1.19.2. Differential expression analysis was performed using DESeq2 (v1.44.0) on featureCounts-derived gene-level counts. Sample annotations were matched by sample identifiers, and a design formula of ~batch + class was used to account for batch effects arising from independent experimental replicates. Lowly expressed genes were filtered (≥10 counts in ≥2 samples), and normalized counts were used for dispersion estimation and model fitting under default DESeq2 settings with independent filtering. Variance-stabilizing transformation (blind = FALSE) was applied for downstream analyses.

Pathway enrichment was assessed by Gene Set Enrichment Analysis (GSEA v4.3.2; Broad Institute)^76^ using MSigDB v2023.1 gene sets (H, C2, C5) on ranked gene lists. GSEA was also applied to publicly available datasets (GSE141331 and GSE141335), with samples grouped by IB responsiveness. For GSE141335, only patients showing concordance between clinical response and ex vivo IB testing were included (9 resistant, 13 sensitive). Ferroptosis suppressor gene sets were curated from FerrDb V2^77^, restricted to genes with TPM ≥ 5 across MCL cell lines (Supplementary Table 1). Analyses were run with 1000 permutations, and gene sets with FDR q < 0.1 were considered significantly enriched.

KEGG pathway enrichment analysis was performed using the DAVID Bioinformatics Resources^78^ online platform. A total of 425 overlapping genes from BRG1^WT^ Cut&Run peaks (vs. IgG; MACS2 *p* < 0.001) and PRO-seq DEGs (FHD-286 vs. control; FDR < 0.1, |FC| > 1.5) in MINO cells were analyzed. Enriched pathways were identified using default DAVID parameters, with those showing FDR < 0.1 considered significant and presented in the plot.

### PRO-seq analysis

Chromatin or cells were incubated in the nuclear run-on reaction condition (5 mM Tris-HCl pH 8.0, 2.5 mM MgCl2, 0.5 mM DTT, 150 mM KCl, 0.5% Sarkosyl, 0.4 units/μL of RNase inhibitor) with biotin-NTPs and rNTPs supplied (18.75 μM rATP, 18.75 μM rGTP, 1.875 μM biotin-11-CTP, 1.875 μM biotin-11-UTP for uPRO; 18.75 μM rATP, 18.75 μM rGTP, 18.75 μM rUTP, 0.75 μM CTP, 7.5 μM biotin-11-CTP for pChRO) for 5 min at 37 °C. Run-On RNA was extracted using TRIzol, and fragmented under 0.2 N NaOH for 15 min on ice. Fragmented RNA was neutralized and buffer exchanged by passing through P-6 columns (Biorad). 3′ RNA adapter (/5Phos/NNNNNNNNAGAUCGGAAGAGCACACGUCUG/ 3InvdT/) is ligated at 5 μM concentration for 1 h at room temperature using T4 RNA ligase (NEB), followed by 2 consecutive streptavidin bead bindings and extractions. Extracted RNA is converted to cDNA using template switch reverse transcription with 1 μM RT primer (GTGACTGGAGTTCAGACGTGTGCTCTTCCGATC), 3.75 μM Template Switch Oligo (TCTTTCCCTACACGACGCTCTTCCGATCTrGrGrG), 1x Template Switch Enzyme and Buffer (NEB) at 42 °C for 30 min. After a SPRI bead clean-up, the cDNA is PCR amplified up to 20 cycles using primers compatible with Illumina TRU-seq sequencing.

### Single-cell RNA sequencing (scRNA-seq) analysis

MCL specimens were obtained from patients enrolled in the phase I clinical trial of ibrutinib in combination with palbociclib (PALIBR)^79^. scRNA-seq was performed on peripheral blood mononuclear cells and/or tissue-derived samples, including bone marrow and lymph node specimens, collected longitudinally during therapy, followed by bioinformatic processing^80^. Raw scRNA-seq data generated in this study have been deposited in the NCBI Sequence Read Archive under accession number PRJNA610037.

Raw sequencing data were processed to generate gene expression count matrices using standard pipelines. Quality control filtering was applied to exclude low-quality cells and technical artifacts; specifically, cells with low numbers of detected genes, high mitochondrial gene content indicative of cellular stress, or likely doublets/multiplets were removed. After filtering, gene expression values were normalized and log-transformed, and 2000 highly variable genes were identified for downstream analysis. Principal component analysis (PCA) was performed using the highly variable genes to capture the major sources of transcriptional variation, and the top 100 principal components were retained. To correct for batch effects arising from differences across patients, sample types, and experimental conditions, we applied the Harmony algorithm^81^ using these principal components, enabling integration of all datasets into a shared low-dimensional space while preserving biological variability.

Unsupervised clustering was performed using the shared nearest neighbor (SNN) modularity optimization algorithm implemented in Seurat (v4.0)^82^, which groups cells based on transcriptional similarity without prior assumptions. For visualization of cellular heterogeneity, t-distributed stochastic neighbor embedding (t-SNE) was applied^83^. Cell type annotation was conducted using a multi-step approach. First, major immune cell populations were identified based on canonical hematopoietic marker genes to distinguish B cells, T cells, and myeloid populations. Subsequently, automated reference-based annotation was performed using the SingleR framework^84^, leveraging multiple reference datasets including Blueprint^85^ and ENCODE, as well as an in-house bulk RNA-seq reference comprising CD19⁺ cells from 57 MCL patient samples and CD19⁺ B cells from 4 healthy donors.

Malignant MCL cells were distinguished from normal B cells based on co-expression of B-cell marker CD19 together with elevated expression of CCND1, a hallmark genetic feature of MCL. These features were integrated with transcriptional similarity to MCL-specific reference profiles and divergence from normal B-cell signatures derived from healthy donor samples. This classification was further supported by comparison with bulk RNA-seq–derived disease-specific gene expression programs and, where available, concordance with orthogonal datasets including whole-transcriptome and whole-exome sequencing of matched samples, consistent with known genomic alterations characteristic of MCL^80^. Unsupervised clustering of malignant MCL cells reproducibly identified four transcriptionally distinct clusters across samples. Cluster 1 exhibited a resting-like transcriptional profile resembling quiescent B cells. Cluster 2 reflected an activated state enriched for BCR signaling, cytokine response, and inflammatory pathways. Cluster 3 represented a non-cycling, persistent population with features of stress adaptation that increased with disease progression. Cluster 4 comprised a highly proliferative population characterized by strong cell cycle and biosynthetic gene expression programs and was enriched in progressive disease.

### Statistics and reproducibility

Experimental sample sizes were determined based on preliminary data. For numerical variables, all results are expressed as mean ± s.d., unless otherwise indicated. Normality was assessed before applying two-sample Student’s t-tests or one-way ANOVA for group comparisons. Unless specified, differences between two groups were analyzed using two-sided Student’s t-tests, whereas comparisons among multiple groups were analyzed using one-way ANOVA followed by Tukey’s post-hoc test to adjust *p* values for multiple comparisons. The Kaplan-Meier method was used to estimate survival probability, and the log-rank test was used to compare the overall survival difference between groups. All statistical tests were two-sided, and differences were considered significant when *p* < 0.05. GraphPad Prism (ver. 10) software was used for all statistical analyses. Experiments were repeated independently at least three times with similar results. Representative immunoblots and flow cytometry plots are shown from independent experiments that yielded comparable results; the number of independent repeats is indicated in the corresponding figure legends. Unless otherwise indicated, *n* denotes biologically independent samples or experiments. For in vitro cell-line assays, *n* represents biologically independent experiments performed using independently cultured cells. For patient-derived samples, *n* represents independent patient specimens. For animal studies, *n* represents individual mice. When technical replicates were included, they were averaged to generate a single value for each biological replicate before statistical analysis.

### Reporting summary

Further information on research design is available in the Nature Portfolio Reporting Summary linked to this article.

## Supplementary information

Supplementary Information

Description of Additional Supplementary Files

Supplementary Data 1

Supplementary Data 2

Supplementary Data 3

Supplementary Data 4

Supplementary Data 5

Supplementary Data 6

Supplementary Data 7

Supplementary Data 8

Supplementary Data 9

Supplementary Data 10

Reporting Summary

Transparent Peer Review file

## Source data

Source data

## Data Availability

The data that support the findings of this study are available within the article and its Supplementary Tables. RNA-seq, PRO-seq, ATAC-seq, and Cut&Run data were generated for this study and can be accessed on the Gene Expression Omnibus (GEO) database with accession numbers GSE305144 and GSE303985. scRNA-seq data can be found on PRJNA610037. All numerical raw data and scanned images of unprocessed blots are shown in the Source data. Source data are provided with this paper.
