# Supplementary material for: BRG1-mediated suppression of ferroptosis underlies BTK inhibitor resistance
Source: Nat Commun. 2026 Jul 2;17:8220. doi: 10.1038/s41467-026-75123-4 (PMC13462549; doi:10.1038/s41467-026-75123-4)
Supplement: Supplementary file 1 [file 41467_2026_75123_MOESM13_ESM.pdf]

nature portfolio

Corresponding author(s): Jihye Paik & Hongwu Zheng

Last updated by author(s): Jun 3, 2026

Reporting Summary

Nature Portfolio wishes to improve the reproducibility of the work that we publish. This form provides structure for consistency and transparency in reporting. For further information on Nature Portfolio policies, see our Editorial Policies and the Editorial Policy Checklist.

Statistics

For all statistical analyses, confirm that the following items are present in the figure legend, table legend, main text, or Methods section.

n/a	Confirmed	
☐	☒	The exact sample size (*n*) for each experimental group/condition, given as a discrete number and unit of measurement
☐	☒	A statement on whether measurements were taken from distinct samples or whether the same sample was measured repeatedly
☐	☒	The statistical test(s) used AND whether they are one- or two-sided<br>*Only common tests should be described solely by name; describe more complex techniques in the Methods section.*
☒	☐	A description of all covariates tested
☐	☒	A description of any assumptions or corrections, such as tests of normality and adjustment for multiple comparisons
☐	☒	A full description of the statistical parameters including central tendency (e.g. means) or other basic estimates (e.g. regression coefficient) AND variation (e.g. standard deviation) or associated estimates of uncertainty (e.g. confidence intervals)
☐	☒	For null hypothesis testing, the test statistic (e.g. *F*, *t*, *r*) with confidence intervals, effect sizes, degrees of freedom and *P* value noted<br>*Give P values as exact values whenever suitable.*
☒	☐	For Bayesian analysis, information on the choice of priors and Markov chain Monte Carlo settings
☒	☐	For hierarchical and complex designs, identification of the appropriate level for tests and full reporting of outcomes
☒	☐	Estimates of effect sizes (e.g. Cohen's *d*, Pearson's *r*), indicating how they were calculated

Our web collection on statistics for biologists contains articles on many of the points above.

Software and code

Policy information about availability of computer code

Data collection

1) All flow cytometry data were collected via BD FACSymphony A1 (BD Bioscience)
2) All immunoblot images were collected on Azure 600 The Ultimate Western Imaging System (Azure Biosystems)
3) All mitochondrial oxygen consumption was measured using O2K oxygraph (Oroboros Instruments)
4) ATAC-seq libraries were sequenced on Element AVITI System (Element Biosciences)
5) mRNA library was sequenced on HiSeq 4000 sequencer (Illumina)
6) Cut&Run library was sequenced on NovaSeq X Plus (Illumina)
7) PRO-seq libraries were sequenced on TRU-seq sequencer (Illumina)
8) In vivo bioluminescent imaging of CDX mouse was conducted using IVIS Spectrum system (Caliper Life Sciences)

Data analysis	1) All flow cytometry data were analyzed with FlowJo v.10.8.1<br>2) All the collected immunoblot images were processed (e.g. rotated/cropped) using ImageJ v1.52a (NIH).<br>3) Respiration rates were recorded using DataLab software and analyzed in Microcal origin (OriginLab).<br>4) ATAC-seq data were analyzed with Bowtie2 v2.5.2; SAMtools (v1.19.1); Picard MarkDuplicates (v3.1.1); Deeptools v3.5.6; MACS2 v2.2.9.1; HOMER v4.11, and displayed with Integrative Genomics Viewer (IGV) v2.17.2.<br>5) Cut&Run data were analyzed with Bowtie v2.3.4.1; Picard MarkDuplicates v2.16.0; MACS2 v2.2.9.1; BEDTools v2.29.2; featureCounts v1.6.1; DESeq v1.46.0, HOMER v4.11, and displayed with Integrative Genomics Viewer (IGV) v2.17.2.<br>6) mRNA-seq data were analyzed with FastQC (v0.12.1); Subread v2.0.6; and DEseq2 (v1.44.0).<br>7) Quantification of In vivo bioluminescent images were performed using Living Image v4.5.4 (PerkinElmer).<br><br>Unless otherwise specified in the Methods, data management, analysis and graphing were performed with Prism v.10 (Graphpad) or Excel 2016 (Microsoft). For bioinformatic analysis, GSEA v4.4.0 (Broad Institute) and DAVID Bioinformatics Resources online platform were utilized.

For manuscripts utilizing custom algorithms or software that are central to the research but not yet described in published literature, software must be made available to editors and reviewers. We strongly encourage code deposition in a community repository (e.g. GitHub). See the Nature Portfolio guidelines for submitting code & software for further information.

Data

Policy information about availability of data

All manuscripts must include a data availability statement. This statement should provide the following information, where applicable:

- Accession codes, unique identifiers, or web links for publicly available datasets
- A description of any restrictions on data availability
- For clinical datasets or third party data, please ensure that the statement adheres to our policy

The data that support the findings of this study are available within the article and its Supplementary Tables. RNA-seq, PRO-seq, ATAC-seq, Cut&Run, and scRNA-seq data can be found on Gene Expression Omnibus (GEO) database with accession number GSE305144, GSE303985, and PRJNA610037. All numerical raw data and scanned images of unprocessed blots are shown in Source Data.

Research involving human participants, their data, or biological material

Policy information about studies with human participants or human data. See also policy information about sex, gender (identity/presentation), and sexual orientation and race, ethnicity and racism.

Reporting on sex and gender	Non-defined
Reporting on race, ethnicity, or other socially relevant groupings	Non-defined
Population characteristics	Ibrutinib-resistant, relapsed MCL patients with TP53 wild-type and mutation.
Recruitment	N/A
Ethics oversight	This study was approved by the Institutional Review Board of The Ohio State University in accordance with the Declaration of Helsinki.

Note that full information on the approval of the study protocol must also be provided in the manuscript.

Field-specific reporting

Please select the one below that is the best fit for your research. If you are not sure, read the appropriate sections before making your selection.

☒ Life sciences ☐ Behavioural & social sciences ☐ Ecological, evolutionary & environmental sciences

For a reference copy of the document with all sections, see nature.com/documents/nr-reporting-summary-flat.pdf

Life sciences study design

All studies must disclose on these points even when the disclosure is negative.

Sample size	-For in vitro experiments, sufficient sample sizes (enough for any reasonable reader to find confident) were chosen based on the author's experience of what is necessary to provide a convincing and statistically significant result. The authors have previously published peer reviewed papers demonstrating clear findings with similar effect sizes.<br>-For in vivo studies, 3-5 mice per group were chosen based on previous experiments, and following the Care and Use of Laboratory Animals guidelines of Weill Cornell Medical College.<br>-For ex vivo experiments, sample size was determined by availability of patients. No statistical methods were used to predetermine sample sizes, but included as many as possible.<br><br>All sample size is indicated in the figure legends accordingly.

Data exclusions	No exclusion criteria were pre-established in our analysis. No data were excluded throughout the study, except for in vitro experiment samples with technical issues (e.g. loss or damaged sample condition).
Replication	Unless indicated, all in vitro and ex vivo results were obtained from n≥3 independent biological samples collected over n≥3 independent experiments to ensure reliability of our data. For in vivo experiments, biological replicates are always represented. The precise number of sample size/replicates are mentioned in the figure legends.
Randomization	All mice were randomly separated into each indicated groups according to their initial tumor burden assessed upon bioluminescent signals of each mice measured at d0 (prior to drug administration).
Blinding	Blinding was not relevant for in vitro/ex vivo experiments and no blinding was implemented during in vivo experiments due to the requirement of cage labeling.

Reporting for specific materials, systems and methods

We require information from authors about some types of materials, experimental systems and methods used in many studies. Here, indicate whether each material, system or method listed is relevant to your study. If you are not sure if a list item applies to your research, read the appropriate section before selecting a response.

Materials & experimental systems

n/a	Involved in the study
☐	☒ Antibodies
☐	☒ Eukaryotic cell lines
☒	☐ Palaeontology and archaeology
☐	☒ Animals and other organisms
☒	☐ Clinical data
☒	☐ Dual use research of concern
☒	☐ Plants

Methods

n/a	Involved in the study
☐	☒ ChIP-seq
☐	☒ Flow cytometry
☒	☐ MRI-based neuroimaging

Antibodies

Antibodies used	1. Antibodies used for Cut&Run (antibody clone/catalog number/source)<br>-anti-HA-tag C29F4 / #3724 / Cell Signaling Technologies<br>-anti-H3K27Ac / #ab4729 / Abcam<br>-anti-BRG1 / #13-2002 / EpiCypher<br>-normal IgG / #12709 / EpiCypher<br>-goat anti-rabbit IgG / #ABE1294 / Thermofisher Scientific<br>-anti-GFP / #ab290 / Abcam<br><br>2. Antibodies used for immunoblot analysis (antibody clone/catalog number/source)<br>-anti-GFP / #ab290 / Abcam<br>-anti-NDUFA4L2 / #16490-1-AP / Thermofisher Scientific<br>-anti-FTH1 / #4393 / Cell Signaling Technology<br>-anti-BTK / #8547 / Cell Signaling Technology<br>-anti-pBTK (Y223) / #87457 / Cell Signaling Technology<br>-anti-MEF2B / #sc-517433 / Santa Cruz Biotechnology<br>-anti-TUBA / #2144 / Cell Signaling Technology<br>-anti-PLCγ2 / #3872 / Cell Signaling Technology<br>-anti-pPLCγ2 (Y1217) / #3871 / Cell Signaling Technology<br>-anti-SPI1 / #2258 / Cell Signaling Technology<br>-anti-ACTB / #sc-47778 / Santa Cruz Biotechnology<br>-anti-Vinculin / #13901 / Cell Signaling Technology<br>-Goat anti-mouse IgG HRP / #31430 / ThermoFisher Scientific<br>-Goat anti-rabbit IgG HRP / #31463 / ThermoFisher Scientific
Validation	All antibodies used are commercially available, validated by the manufacturer and used as listed in Supplementary Table 6.

Eukaryotic cell lines

Policy information about cell lines and Sex and Gender in Research

Cell line source(s)	JEKO1, MAVER1, MINO, Z138, REC1, and HEK293T cells were purchased from ATCC. CCMCL1, UPN1, and Sp49 cell lines were kindly provided by Dr. Selina Chen-Kiang (Weill Cornell Medicine), Dr. Samir Parekh (Mount Sanai) and Dr. Lalit Sehgal (Ohio State University), respectively.
Authentication	No cell-line authentication method was used. But the cell lines were authenticated by ATCC and continuously monitored through microscopic observation.

Mycoplasma contamination	All cell lines were repeatedly tested for mycoplasma contamination by PCR.
Commonly misidentified lines (See ICLAC register)	N/A

Animals and other research organisms

Policy information about studies involving animals; ARRIVE guidelines recommended for reporting animal research, and Sex and Gender in Research

Laboratory animals	NSG (NOD.Cg-Prkdcscidll2rgtm1Wjl/SzJ) mice were implanted with BTKI-resistant CCMCL1 (CCMCL1R) cell lines to derive CDX model. Details regarding the experiment are mentioned in the method section.
Wild animals	N/A. The study did not use wild animals.
Reporting on sex	Gender was not considered in the study design.
Field-collected samples	N/A. The study did not involve samples from the fields.
Ethics oversight	Use of laboratory mice were approved by the Weill Cornell Medicine Institutional Animal Care and Usage Committee (IACUC protocol 2018-0039).

Note that full information on the approval of the study protocol must also be provided in the manuscript.

Plants

Seed stocks	N/A
Novel plant genotypes	N/A
Authentication	N/A

ChIP-seq

Data deposition

☒ Confirm that both raw and final processed data have been deposited in a public database such as GEO.

☒ Confirm that you have deposited or provided access to graph files (e.g. BED files) for the called peaks.

Data access links *May remain private before publication.*	To review GEO accession GSE305144:<br>https://www.ncbi.nlm.nih.gov/geo/query/acc.cgi?acc=GSE305144
Files in database submission	JEKO parent HA_1<br>JEKO parent HA_2<br>JEKO MEF2B HA_1<br>JEKO MEF2B HA_2<br>MINO BRG WT_H3K27Ac<br>MINO BRG T910M_H3K27Ac<br>MINO BRG WT_BRG1<br>MINO BRG1 WT_IgG<br>MINO BRG1 WT_GFP_1<br>MINO BRG1 WT_GFP_2<br>MINO BRG1 WT_GFP_3<br>MINO BRG1 WT_GFP_4<br>MINO BRG1 T910M_GFP_1<br>MINO BRG1 T910M_GFP_2<br>MINO BRG1 T910M_GFP_3<br>MINO BRG1 T910M_GFP_4
Genome browser session (e.g. UCSC)	N/A

Methodology

Replicates	2-4
Sequencing depth	Cut&Run sequencing was performed using dual-indexed libraries. Each sample generated ~10 million reads. Sequencing depth was 15-30M per sample
Antibodies	-anti-HA-tag C29F4 / #3724 / Cell Signaling Technologies<br>-anti-H3K27Ac / #ab4729 / Abcam<br>-anti-BRG1 / #13-2002 / EpiCypher<br>-normal IgG / #12709 / EpiCypher<br>-goat anti-rabbit IgG / #ABE1294 / Thermofisher Scientific<br>-anti-GFP / #Ab290 / Abcam
Peak calling parameters	The MACS v2.2.9.1 was used for peak calling with following parameters: macs2 callpeak -t "$bamsortrmdup" -c "$inputbam" -f BAM -g "$macsgen" --nomodel --pvalue 0.001 -n "$macsPeaks"
Data quality	MACS p-value<0.0001 were considered significant.
Software	Sequencing reads were aligned to hg38 reference genome using Bowtie v2.3.4.1. Peak calling was performed by MACS2 v2.2.9.1 with default parameters, using matched IgG controls. Normalized read density profiles were generated with BEDTools v2.29.2. Read density of specific genomic regions were displayed using Integrative Genomics Viewer (IGV) v2.17.2. A global peak atlas was constructed by merging peaks within 500 bp and quantifying reads using featureCounts v1.6.1; counts were normalized by sequencing depth and differential enrichment was calculated with DESeq2 v1.46.0. Transcription factor motif enrichment analysis was performed on differential BRG1 binding sites using HOMER (v4.11) findMotifsGenome.pl.

Flow Cytometry

Plots

Confirm that:

☒ The axis labels state the marker and fluorochrome used (e.g. CD4-FITC).

☒ The axis scales are clearly visible. Include numbers along axes only for bottom left plot of group (a 'group' is an analysis of identical markers).

☒ All plots are contour plots with outliers or pseudocolor plots.

☒ A numerical value for number of cells or percentage (with statistics) is provided.

Methodology

Sample preparation	-For cell death analysis, 5.0x10^4 MCL cells in 2 mL were treated with compounds. After 4 days, the fraction of dead cells was determined by FACS analysis for % of To-Pro-3-staining positive cells.<br>-For competition-based proliferation assays, 1x10^6 cells were transduced with lentivirus encoding sgRNA linked with GFP. After 4 days, GFP positive cell population was determined every 3-4 days, time-dependently.<br>-To detect oxidative stress and ROS levels, cells in culture media were incubated with 0.5 µM of CellROX™ Deep Red Reagent (ThermoFisher Scientific, C10422) for 1 h.<br>-To measure labile iron levels, cells were harvested, washed once with HBSS and incubated with 1 µM FerroOrange fluorescence probe (Cell Signaling Technology, 36104S) for 30 min at 37 °C.<br>-To measure intracellular lipid peroxidation, cells in culture media were incubated at 5 µM BODIPY-C11 (Cayman Chemical, #27086) or 10 µM BODIPY 665/676 (ThermoFisher Scientific, B3932) probe in growth medium for 1 h at 37 °C.<br>-To measure mitochondrial mass, cells were harvested, washed with PBS, and incubated with 50 nM MitoTracker® Deep Red FM (ThermoFisher Scientific, #46753) for 30 min at 37 °C.<br>-To measure the level of mitophagy, cells were lentivirally infected to express mt-mKeima (Addgene plasmid #131626).
Instrument	BD FACsymphony A1
Software	BD FACSDiva software was used to acquire data. For further analysis, FlowJo v.10.8.1 was utilized.
Cell population abundance	N/A No post-sort fractions were collected.
Gating strategy	-For cell death assays using To-Pro-3, analysis was performed across the entire cell population, with unlabeled negative controls and healthy control cells used to set the gating threshold for determining the fraction of dead cells in each sample.<br>-For ROS (CellROX™ Deep Red), labile iron (FerroOrange), mitochondrial mass (MitoTracker) measurement, cells were gated on FSC/SSC to exclude cell debris/doublets and to define the live singlet population. Within this gate, fluorescent intensity in relevant channels was quantified, and boundaries between positive and negative staining populations were established using unstained negative controls.<br>-For lipid peroxidation, cells were gated on FSC/SSC to exclude cell debris/doublets and to define the live singlet population. Within this gate, fluorescent intensity was measured in two channels corresponding to the non-oxidized and oxidized BODIPY populations. The oxidized shift was defined using known ferroptosis inducers as positive control and confirmed by suppression with ferrostatin-1 as a negative control.<br>-For mitophagy level measurement, cells were gated on FSC/SSC to exclude cell debris/doublets and to define the live singlet

population. Within this gate, mt-Keima fluorescence was detected at 610/20 nm following excitation at 405 nm (mitochondrial) and 565 nm (lysosomal). Suppression was confirmed by chloroquine as a negative control.

☒ Tick this box to confirm that a figure exemplifying the gating strategy is provided in the Supplementary Information.
