# Supplementary material for: BRG1-mediated suppression of ferroptosis underlies BTK inhibitor resistance
Source: Nat Commun. 2026 Jul 2;17:8220. doi: 10.1038/s41467-026-75123-4 (PMC13462549; doi:10.1038/s41467-026-75123-4)
Supplement: Supplementary file 2 [file 41467_2026_75123_MOESM14_ESM.pdf]

nature portfolio

Peer Review File

BRG1-Mediated Suppression of Ferroptosis Underlies BTK Inhibitor Resistance

Corresponding Author: Dr Jihye Paik

This file contains all reviewer reports in order by version, followed by all author rebuttals in order by version.

Version 0:

Reviewer comments:

Reviewer #1

(Remarks to the Author)
The manuscript by Hwang et al. addresses an important and timely topic — the role of BRG1 in mediating resistance to BTK inhibitors in mantle cell lymphoma (MCL). The work attempts to link chromatin remodeling, ferroptosis regulation, and BTK inhibitor resistance, but falls short in providing convincing, rigorous evidence for many of the key claims.

Major concerns.

1. The proposed BRG1–MEF2B–NDUFA4L2 axis is intriguing but lacks direct evidence for causality. For instance, while MEF2B and NDUFA4L2 are implicated, the precise molecular mechanisms by which they suppress ferroptosis (beyond mitochondrial respiration changes) are not well defined. The claim that NDUFA4L2 "mediates" ferroptosis resistance is supported mostly by correlative rescue experiments, but not by direct mechanistic insight into how altered mitochondrial activity prevents lipid peroxidation or iron-dependent death.

2.The proposed linear pathway from mutant BRG1 to MEF2B upregulation to NDUFA4L2/FTH1 induction is asserted but not conclusively proven.
BRG1 to MEF2B: The data show a correlation between mutant BRG1 expression and MEF2B levels, but no direct evidence places MEF2B as a transcriptional target of BRG1. A Cut&Run experiment demonstrating direct BRG1 binding to the MEF2B promoter or enhancer is conspicuously absent. The upregulation could be an indirect downstream effect.
MEF2B to Target Genes: While MEF2B Cut&Run data is presented for NDUFA4L2 and FTH1, the functional validation relies heavily on depletion/rescue and overexpression experiments. The study does not utilize, for instance, a dominant-negative MEF2B or promoter mutagenesis to definitively show that MEF2B's transcriptional activity is required for the regulation of these specific genes in this context.

3. The authors proposed that NDUFA4L2 negatively regulates ferroptosis by reducing oxygen consumption and inhibiting mitochondrial activity. However, in the abstract, they claimed NDUFA4L2 blocks mitochondrial damage-dependent ferroptosis. It sounds controversial.

4. Overexpression of NDUFA4L2 inhibits oxygen consumption, which may activate AMPK signal, a negative regulator of ferroptosis. Does BTKi resistant cells have higher AMPK signal, therefore inhibiting ferroptosis?

5.The authors often overstate the novelty or specificity of their findings. For example, the link between BTK inhibition, mitochondrial dysfunction, and ferroptosis has been explored in other contexts (e.g., recent studies on BTKi and ferroptosis PMID: 36693553), yet the manuscript does not adequately contextualize their findings relative to the existing literature.

Minor questions:
1.The authors should provide western blot verification for all genetic modified cells in this manuscript. The data are not limited as following: Figure S1B, CRISPB-Cas9 mediated KO of BTK should be verified by western blot. Figure S2A. The expression of BRG1wt and BRG1K785R in MINO or JEKO cells should be verified by western blot. Figure CRISPB-Cas9 mediated KO of MEF2B and rescued cells should be verified by western blot.
2.Figure 1A and B: It was reported that necrostatin-1 has ferroptosis inhibition activity. necrostatin-1S is a more specific necroptosis inhibitor.

Reviewer #2

(Remarks to the Author)
Resistance to BTK inhibitors (BTKi) in MCL remains a critical therapeutic challenge. The study by Hwang and colleagues suggests that ferroptosis suppression is a major mechanism driving BTKi resistance. They identify aberrant BRG1 as a key regulator of this process, orchestrating transcriptional programs that inhibit ferroptosis pathways independently of BTK signaling. Furthermore, they show that BRG1 mutations may increase cellular reliance on BRG1 activity, enabling alternative resistance mechanisms. Mechanistically, MEF2B mediates BRG1-driven ferroptosis suppression through direct activation of NDUFA4L2, which functions as a pivotal downstream effector of the BRG1T910M-MEF2B axis. NDUFA4L2 protects MCL cells from BTKi-induced mitochondrial damage by redirecting metabolism away from oxidative phosphorylation, thereby reducing reactive oxygen species and iron release. Although these findings provide a strong preclinical rationale for therapeutic strategies combining BTKi with BRG1 inhibition (FHD-286) to overcome resistance in MCL, important limitations should be addressed, particularly the specificity of the FHD-286 and the limited validation in primary MCL samples.

Major Comments:
1. Figure 1. The data presented are from cell lines without clinical correlation. The mechanistic link between ferroptosis suppression and BTKi resistance should be validated in primary MCL samples. Figure 1G. GSEA shows enrichment of ferroptosis suppressor genes in resistant cells (JEKO and patients), However, functional validation (e.g., Fe^{2+}, ROS or Lipid peroxidation levels or death upon b-Me, NAC) is not described in the excerpt.
2. Figure 2G-L. Authors should demonstrate effective and specific knockout of BGR1 in Figure 2G-H. Are de levels of BRG1 similar in IB resistant than in sensitive cell lines (compared BRG1 levels in CCMCL1R and JEKO1R vs CCMCL1S and JEKO1S cells, respectively). Cell death in sgBRG1 is expected, as BRG1 has essential function for living cells. The innovative finding will be to show that cell death upon BRG1 knockout is due to ferroptosis. Comparison of experiments shown in Fig 2I should be done in sgBrg1 cells and compared them with control and mutant cells.
3. Resistance to IB and sensibility to FHD-286 in BRG1MUT MCL primary cases should be analyzed in comparison to BRG1WT and IB sensitive and resistant cases.
4. FHD-286 is described as a dual BRG1/BRM inhibitor. The manuscript does not fully address this dual activity, nor does it demonstrate that the observed physiological effects are specifically mediated through BRG1. This point is important because ATAC-seq analysis of BCR-related genes shows a less reduction upon FHD-286 treatment compared to sgBRG1. Additional evidence confirming that the effects of FHD-286 are primarily due to BRG1 inhibition would help rule out potential off-target contributions.
5. Figure 3A. To demonstrate that FHD-286 is directly inhibiting BRG1 and its transcriptional program, authors should compared, by Venny, FHD-286 and BGR1 specific DEGs, to discard of target effects. Moreover BGR1 Cut&Run experiments in FHD-286 treated and sgBRG1 cells should be compared to control cells.
6. Fig 4A. Is MEF2B directly regulated by BRG1? Although increased MEF2B expression was observed in BRG1T910M vs. BRG1WT cells (Fig.4B-D), Fig.4D seems to indicate that MEF2B degradation is compromise in BRG1T910M cells. To demonstrate BRG1T910M is directly regulating MEF2B transcription in MCL cells, Cut&Run experiments in respectively cell lines, comparing binding of BRG1T910M vs. BRG1WT proteins to MEF2B regulatory regions should be shown. What are the mRNA and protein MEF2B levels in IB resistant vs. sensitive MCL cell lines and cases (CCMCL1R, JEKO1R and patientsR vs. CCMCL1S, JEKO1S patientss cells). Incorporating these data could strengthen authors's claim, proposing that BRG1 contributes to BTKi resistance through direct transcriptional regulation of BCR-related and -not related genes (MEF2B) by confirming BRG1's direct involvement.
7. The manuscript suggests that BRG1T910M activates a BCR-independent pathway via MEF2B, leading to upregulation of NDUFA4L2 and FTH1 and consequently BRG1 inhibition by FHD-286 treatments would induce cell death thought increasing ROS and Fe^{2+} levels inducing ferroptosis cell death. It would be informative to determine whether the MEF2B, NDUFA4L2 and FTH1 are also downregulated in FHD-286 treated vs control and how its levels are in sgBRG1 vs control MCL cell lines or in IB resistant vs. sensitive MCL cell lines and cases (CCMCL1R, JEKO1R and patientsR vs. CCMCL1S, JEKO1S patientss cells). This comparison could provide additional mechanistic insight.
8. MEF2B, NDUFA4L2 and FTH1 expression is increased in BRG1T910M compared to BRG1WT. MEF2B is directly regulating NDUFA4L2 and FTH1 expression. Is BRG1 also involved as cofactor in the regulation of these two genes? ATAC-seq or Cut&Run at NDUFA4L2 and FTH1 regulatory regions in BRG1T910M compared to BRG1WT could provide additional mechanistic insight.
9. The degree of BTKi resistance in BRG1T910M compared to BRG1WT cells is not explicitly compared. Demonstrating whether resistance is significantly higher in BRG1T910M compared to BRG1WT cells, potentially due to increased MEF2B, NDUFA4L2, and FTH1 expression, would add clarity to the functional impact of this mutation.
10. Recent studies indicate that BRG1 can act as a cofactor for SOX11 in the transcriptional activation of BCR-related and -not related genes in MCL cells (PMID: 40739087). Since the genes regulated by BRG1 overlap with those influenced by SOX11, the authors should discuss whether BRG1T910M might affect SOX11-mediated transcriptional regulation and if SOX11 it is also involved in MEF2B/NDUFA4L2/FTH1 axis inducing ferroptosis suppression.
11. Figure 7A. The manuscript should clarify whether the observed differences in IC50 values among MCL WT cell lines are associated with varying BRG1 expression levels. This would help contextualize the variability in drug sensitivity.
12. The data presented are from cell lines and mouse models without clinical correlation. Some findings should be validated

in primary MCL cases, (e.g. the mechanistic link between ferroptosis suppression and BTKi resistance, MEF2B, NDUFA4L2, and FTH1 expression levels comparing BTKi-sensitive vs. -resistant samples and/or BRG1T910M versus BRG1WT genotypes). Such validation would significantly enhance the translational relevance of the study.
13. Treatments with PB and FHD are done in IB resistant and BRG1T910M cell lines but not in control and BRG1WT cell lines or patients cells. Due that these drugs also affects BCR-pathway, effects (ROS, Lipid peroxidation and Fe2+ levels and cell death) upon treatments should be compared between BRG1T910M and BRG1WT cell lines or patients cells. Due to the high heterogeneity between MCL cells lines, cell death in Figure 7F should be done also in JEKO1R vs JEKO1S and patients R vs patientsS cells.
14. Figure 7I. Proteins involved in BCR and ferroptosis suppression should be analyzed by IHC.

Minor Comment:
1. Figure 7H. Provide genotype information (BRG1 mutation site in P#2), BRG1 levels, MEF2B/NDUFA4L2/FTH1 expression, and IB resistance status for clarity.

Reviewer #3

(Remarks to the Author)
In this manuscript, the authors are interested in understanding mechanisms underlying resistance to Bruton's tyrosine kinase inhibitors (BTKi) in Mantle cell lymphoma (MCL). They have identified that chromatin remodeler BRG1 suppresses ferroptosis by regulating BTK-dependent survival signaling and BTK independent transcriptional program. BRG1 promotes BTKi resistance by regulating BTK dependent survival signaling. BRG1 mediates BTK independent transcriptional program by upregulating MEF2B. They show that MEF2B directly upregulates the mitochondrial complex 1 subunit NDUFA4L2 resulting in abrogation of mitochondrial damage dependent ferroptosis. They identify suppression of ferroptosis as one of the main reasons for BTK inhibitor resistance which is mediated by BRG1. Finally, they show that BRG1 inhibitor FHD-286 sensitizes resistant MCL cells to BTK inhibitors. The manuscript can be further strengthened by addressing the following comments –

1. Based on the data presented, one would expect BRG1 mutants are gain-of-function in nature. It would be critical to perform ChIP-seq and/or Cut&Run for wildtype and mutant BRG1 and examine how they may differentially regulate gene expression by cross-referencing with PRO-seq/RNA-seq data.
2. In Figure 1G, the authors show that JEKO1 resistant cells have enrichment of ferroptosis suppressor gene sets by GSEA and are unresponsive to BTKi treatment. Do BRG1 T910M cells share the GSEA signature of the resistant cells as the mutant cells also do not respond to BTKi treatment?
3. The authors cite literature that BRG1 is one of the most frequently genes in MCL and is implicated in BTKi resistance and say that is the premise for testing its potential contribution to BTKi. It would be great, if they could find MCL patient data and check for BRG1 910 mutation and determine if the patient's carrying mutation respond to BTK1i from publicly available database.
4. In figure 5C, the authors did Cut & Run to show that MEF2B directly regulates NDUFA4L2 and FTH1. It would be nice if the authors added MEF2B ChIP-qPCR validation. Likewise, the identification of MEF2B is central to the proposed pathway. However, the evidence that BRG1 may regulate MEF2B transcription is only correlative (expression and chromatin accessibility of MEF2B between BRG1 mutants and wildtype). Please perform ChIP-qPCR to establish a direct mechanistic link.
5. In figure 7C, the authors show that BRG1 inhibitor in combination with BTKi sensitizes resistant cells to BTKi. The authors can strengthen this by doing IHC/IF of ferroptosis markers in tumor samples. In addition to ferroptosis markers, they can also include caspase- 3 as a negative control to show that indeed the combination induces ferroptosis and not apoptosis in vivo.
6. The study links BRG1 mutations to ferroptosis regulation and BTKi sensitivity, suggesting a role in primary resistance. However, most data are derived from induced resistant (BTKi-R) cell lines, which model secondary/acquired resistance. Please clarify whether the goal is to explain baseline BTKi sensitivity differences or to understand how BRG1 contributes to acquired resistance. And it would be valuable to show how BRG1 expression or activity changes during the development of resistance or between BTKi-sensitive and -resistant lines.
7. The manuscript reports "134 overlapping DEGs from PRO-seq ($|FC| > 1$) of JEKO1 and RNA-seq ($|FC| > 1.5$) of MINO cells." Combining DEG lists derived from different cell lines and platforms (PRO-seq vs RNA-seq) without controlling for cell-line- or assay-specific effects is not statistically justified. This method may introduce significant confounding variables and potential bias. Please provide a rationale for this intersection approach. Besides, what are the pathway analysis results if the two datasets are analyzed separately? Will the two datasets indicate consistent pathway findings?
8. According to the GEO dataset (GSE305144), each RNA-seq group includes only one sample. Were any biological or technical replicates performed? If standard methods such as DESeq2/edgeR/limma were used, please describe how variance was estimated. Without replicates and proper statistical tests, the validity of all downstream conclusions that rely on these transcriptomic datasets would be fundamentally compromised. The authors must transparently describe the method used to call DEGs and provide statistical significance measures (e.g., p-values, FDR). If no replicates were performed, this must be explicitly stated and all key findings from these datasets must be independently validated with other methods (e.g., qPCR, WB).
9. Different thresholds (0.6, 1.0, and 1.5) are used in different analyses. Please justify these choices and demonstrate whether results are robust under consistent criteria or based on FDR-adjusted significance.

Minor comments

1. To strengthen translational relevance, the authors could explore whether BRG1 mutation or BRG1/MEF2B/NDUFA4C2 axis expression correlates with BTKi response in patient datasets, if available.
2. Since ferroptosis is regulated by multiple pathways, please discuss how dominant this axis is compared with known regulators (e.g., GPX4, SLC7A11).
3. Previous studies (Cell Reports, 2024) have shown that BRG1-deficient cancers are hypersensitive to ferroptosis induction. Please clarify whether BRG1 inhibition alone would be therapeutically sufficient, or whether ferroptosis inducers should be combined for greater efficacy.
4. FHD-286 is a BRG1/BRM inhibitor. Please discuss how to rule out the effect of BRM in this study.
5. The overall methodology description for sequencing and bioinformatic analysis is insufficient.
6. In Fig 3C, the peak for CD79B is not convincing. IgG is blank in almost all of their figures, which doesn't appear to make sense.

Version 1:

Reviewer comments:

Reviewer #1

(Remarks to the Author)
In the revised manuscript, the authors performed more experiments and provided more data. They have addressed all concerns from this reviewer. There is no more question.

Reviewer #2

(Remarks to the Author)
Minor comments:
1. SOX11 has been reported to limit lethal ROS production in MCL by inducing oxidative stress modulation (PMID: 38570586). This effect is mediated through the SOX11:BRG1 complex, which regulates PRDX2 expression (PMID: 40739087), thereby inhibiting ferroptosis-mediated cell death. Considering these findings, additional analyses are required to demonstrate that resistance to BTK inhibitors (ibrutinib, zanubrutinib, pirtobrutinib) mediated by ferroptosis suppression is independent of the SOX11:BRG1 complex.
Moreover, as direct regulation of MEF2B by BRG1 has not been demonstrated in MCL, the absence of changes in Fe^{2+} levels, ROS, lipid peroxidation, ATF4, MEF2B, and related markers upon BRG1 depletion or BRG1T910M ectopic expression in a SOX11-negative MCL cell line would further support a SOX11-independent, BRG1-mediated mechanism of BTKi resistance. Such data would strengthen the proposed ATF4–MEF2B–NDUFA4L2/FTH1 axis as a direct inhibitor of ferroptosis-driven cell death.

2. It should be evaluated whether pharmacological inhibition of BRG1 using alternative BRG1 inhibitors produces effects comparable to those observed with FHD-286 in MCL cell models. This would help establish whether the observed phenotypes are specifically attributable to BRG1 inhibition rather than compound-specific effects.
3. Given that BRG1 is ubiquitously expressed and essential for cell viability, that the BRG1T910M mutation is rare in MCL, and that FHD-286–mediated inhibition is independent of BRG1 expression levels, the authors should discuss how representative and clinically relevant the proposed BRG1–MEF2B–NDUFA4L2/FTH1 axis is in contributing to ferroptosis-mediated BTKi resistance in MCL.
4. The authors should discuss why they have chosen this specific mutation and if other missense mutations affecting the SNF2 or other domains that may affect its catalititc activity may have the same functional effect as the BRG1T910M in MCL.
5. The Supplementary Tables contain multiple errors and inconsistencies and must be revised to provide accurate and experiment-specific information.
o Supplementary Table S2 is inadequately described, and essential methodological and experimental details are missing.
o The same table (Supplementary Table S3) has been incorrectly provided for the PRO-seq, RNA-seq, and CUT&RUN experiments. A similar issue affects the MEF2B DEG and MEF2B CUT&RUN datasets.
o Distinct and appropriately labeled tables should be supplied for each dataset as follows:
Supplementary Table S3: BRG1 WT/MUT PRO-seq
Supplementary Table S4: BRG1 WT/MUT RNA-seq
Supplementary Table S5: BRG1 WT/MUT CUT&RUN-seq
Supplementary Table S6: MEF2B DEG RNA-seq
Supplementary Table S7: MEF2B CUT&RUN-seq
o Please ensure that each table is complete, accurately labeled, and corresponds unambiguously to the associated experiment.

Reviewer #3

(Remarks to the Author)
The authors have addressed most of the concerns and the extensive revision has significantly elevated the mechanistic depth and translational relevance of this study. I only have a few minor clarifications for the authors to consider.
1. The methodology section currently lacks sufficient detail regarding the single-cell analysis. The authors are recommended to explicitly describe the methods and criteria used to distinguish malignant MCL cells from normal B cells. Furthermore,

please include essential methodological details, such as quality control parameters, data integration, and batch-effect correction methods, in the Supplementary Methods.
2. The scRNA-seq analysis identified 4 clusters within the MCL cell population. However, there is currently no functional description of these sub-populations. It would be valuable if the authors could perform differential gene and pathway analysis on these subclusters, and investigate whether the relative proportions differ significantly between CR and PD patients. If a compositional shift exists between the two clinical states, exploring the evolutionary trajectory and differential transcriptional regulation among these clusters would be highly meaningful and could provide deeper insights into how the acquired resistance-associated transcriptional program evolves under BTKi pressure.
3. When comparing the expression levels of the target genes (BRG1, MEF2B, NDUFA4L2, and FTH1) between the PD and CR groups, the statistical analyses should be performed at the sample level. Please provide statistical comparisons between the CR and PD cohorts using sample-level metrics, this can be done either by comparing the average expression level per patient sample or by comparing the proportion of cells expressing the genes of interest across the individual samples.
4. In figure 8a, please state statistical significance.

Version 2:

Reviewer comments:

Reviewer #2

(Remarks to the Author)
Pre-review comments have been successfully addressed.

Reviewer #3

(Remarks to the Author)
The authors have addressed the remaining minor concerns.

Open Access This Peer Review File is licensed under a Creative Commons Attribution 4.0 International License, which permits use, sharing, adaptation, distribution and reproduction in any medium or format, as long as you give appropriate credit to the original author(s) and the source, provide a link to the Creative Commons license, and indicate if changes were made.
In cases where reviewers are anonymous, credit should be given to 'Anonymous Referee' and the source.
The images or other third party material in this Peer Review File are included in the article’s Creative Commons license, unless indicated otherwise in a credit line to the material. If material is not included in the article’s Creative Commons license and your intended use is not permitted by statutory regulation or exceeds the permitted use, you will need to obtain permission directly from the copyright holder.
To view a copy of this license, visit https://creativecommons.org/licenses/by/4.0/

Point by point response to REVIEWER COMMENTS

Reviewer #1 - Ferroptosis

Major concerns.

1. The proposed BRG1–MEF2B–NDUFA4L2 axis is intriguing but lacks direct evidence for causality. For instance, while MEF2B and NDUFA4L2 are implicated, the precise molecular mechanisms by which they suppress ferroptosis (beyond mitochondrial respiration changes) are not well defined. The claim that NDUFA4L2 "mediates" ferroptosis resistance is supported mostly by correlative rescue experiments, but not by direct mechanistic insight into how altered mitochondrial activity prevents lipid peroxidation or iron-dependent death.

: We agree that altered mitochondrial respiration alone is insufficient to explain NDUFA4L2-mediated ferroptosis resistance. Our data support a model in which NDUFA4L2 enforces a preemptive metabolic state that reduces susceptibility to BTKi-induced ferroptosis. By inhibiting mitochondrial Complex I, NDUFA4L2 diverts metabolism away from oxidative phosphorylation, thereby limiting mitochondrial ROS production and establishing an AMPK-high, low-ROS state. AMPK is a central metabolic sensor that has been implicated in the regulation of ferroptosis through modulation of lipid biosynthesis[1].

Consistent with this model, enforced expression of NDUFA4L2 in $BRG1^{WT}$ cells increased AMPK phosphorylation, whereas genetic depletion of NDUFA4L2 in $BRG1^{T910M}$ cells suppressed basal AMPK activation (Reviewer Fig. 1a, b). Modulation of MEF2B expression concordantly altered NDUFA4L2 levels and AMPK phosphorylation (Reviewer Fig. 1c), placing AMPK downstream of the MEF2B-NDUFA4L2 axis. BTKi-resistant MCL cells similarly exhibited elevated basal AMPK activation compared with matched sensitive counterparts (Reviewer Fig. 1d), indicating selection for this metabolic state during resistance acquisition.

Functionally, pharmacologic AMPK activation with metformin suppressed BTKi-induced lipid peroxidation and ferroptotic cell death (Reviewer Fig. 1e, f), whereas selective AMPK inhibition with BAY-3827 enhanced ferroptosis in BRG1-mutant and BTKi-resistant MCL cells, phenocopying NDUFA4L2-dependent resistance (Reviewer Fig. 1g-j). Consistently, treatment with BAY-3827 sensitized BTKi-resistant primary MCL cells to IB, significantly increasing lipid peroxidation (Reviewer Fig. 1k, l). These findings define a mechanism whereby NDUFA4L2 suppresses ferroptosis by reducing mitochondrial ROS output and raising the oxidative threshold required for lipid peroxidation, thereby protecting cells from BTKi-induced ferroptotic stress. These data have been incorporated into Fig. S7 to provide additional mechanistic support.

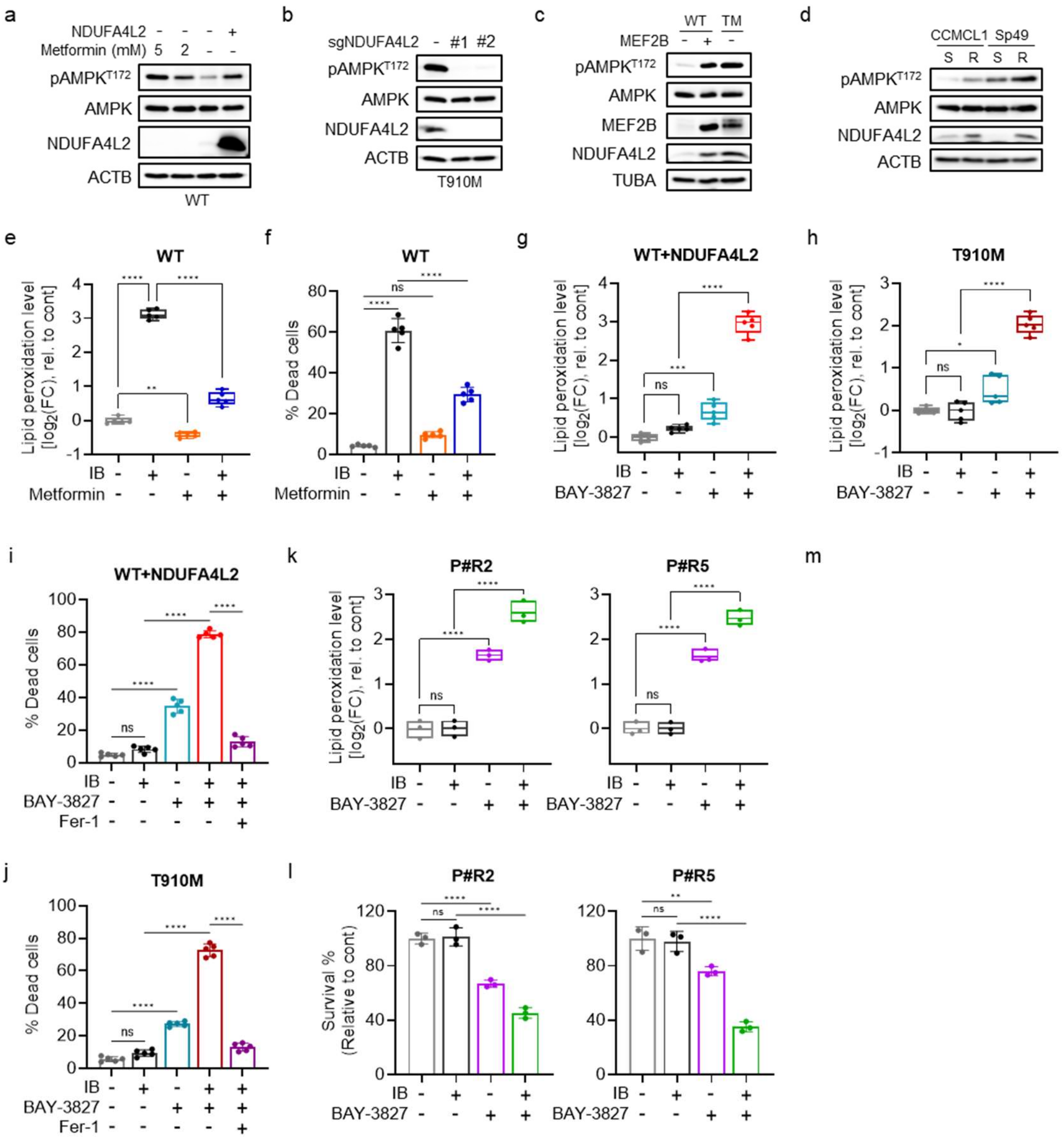

Reviewer Fig. 1. NDUFA4L2 activates AMPK to confer BTKi-induced ferroptosis resistance (a) Immunoblot analysis comparing control and NDUFA4L2-overexpressing JEKO1^{WT} cells. Metformin (2 or 5 mM, 24 h) was used as a positive control for AMPK activation. (b) Immunoblot analysis of NDUFA4L2-depleted JEKO1^{T910M} cells. (c) Immunoblot analysis comparing control and MEF2B-overexpressing JEKO1^{WT} and JEKO1^{T910M} cells. (d) Immunoblot analysis of isogenic BTKi^{S} and BTKi^{R} CCMCL1 and Sp49 cells. (e, f) Changes in lipid peroxidation (e; 72 h, n=5) and cell death (f; 96 h, n=5) following treatment with IB (5 μM), metformin (5 mM), or their combinations in JEKO1^{WT} cells. (g-j) Changes in lipid peroxidation (72 h, n=5) and cell death (96 h, n=5) following IB (5 μM), BAY-3827 (5 μM) or their combinations in NDUFA4L2-overexpressing JEKO1^{WT} (g, i) and JEKO1^{T910M} (h, j) cells. Fer-1 co-treatment (2 μM) was used to assess ferroptotic cell death. (k, l) Changes in lipid peroxidation (48 h, n=3) and cytotoxicity (96 h, n=3) following treatment with IB (5 μM), BAY-3827 (5 μM), or their combinations in BTKi^{R} primary MCL cultures.

2. The proposed linear pathway from mutant BRG1 to MEF2B upregulation to NDUFA4L2/FTH1 induction is asserted but not conclusively proven.

- BRG1 to MEF2B: The data show a correlation between mutant BRG1 expression and MEF2B levels, but no direct evidence places MEF2B as a transcriptional target of BRG1. A Cut&Run experiment demonstrating direct BRG1 binding to the MEF2B promoter or enhancer is conspicuously absent. The upregulation could be an indirect downstream effect.

: Our Cut&Run analysis showed no detectable BRG1 occupancy at MEF2B regulatory regions, indicating that BRG1 does not directly regulate MEF2B (Reviewer Fig. 2a). Instead, our preliminary data suggest that mutant BRG1 promotes a chromatin landscape permissive for the bZIP transcription factor ATF4 (Reviewer Fig. 2b, c). Consistently, ATF4 binding motifs are present within mutant BRG1-induced accessible chromatin regions near the MEF2B transcription start site (Reviewer Fig. 2d), supporting a model in which ATF4 drives MEF2B transcriptional upregulation. Functionally, ATF4 was both necessary and sufficient for MEF2B induction (Reviewer Fig. 2e, f), and induction of ATF4 and MEF2B by aberrant BRG1 activity was suppressed by both genetic and pharmacologic BRG1 inhibition (Reviewer Fig. 2g–i). Together, these data support a model in which BRG1 regulates MEF2B indirectly through an ATF4-associated transcriptional program rather than through direct chromatin binding. The precise mechanism by which aberrant BRG1 activity activates ATF4 remains to be defined and is the subject of our ongoing investigation. This framework underlies subsequent analyses demonstrating MEF2B-dependent activation of NDUFA4L2/FTH1 and ferroptosis suppression.

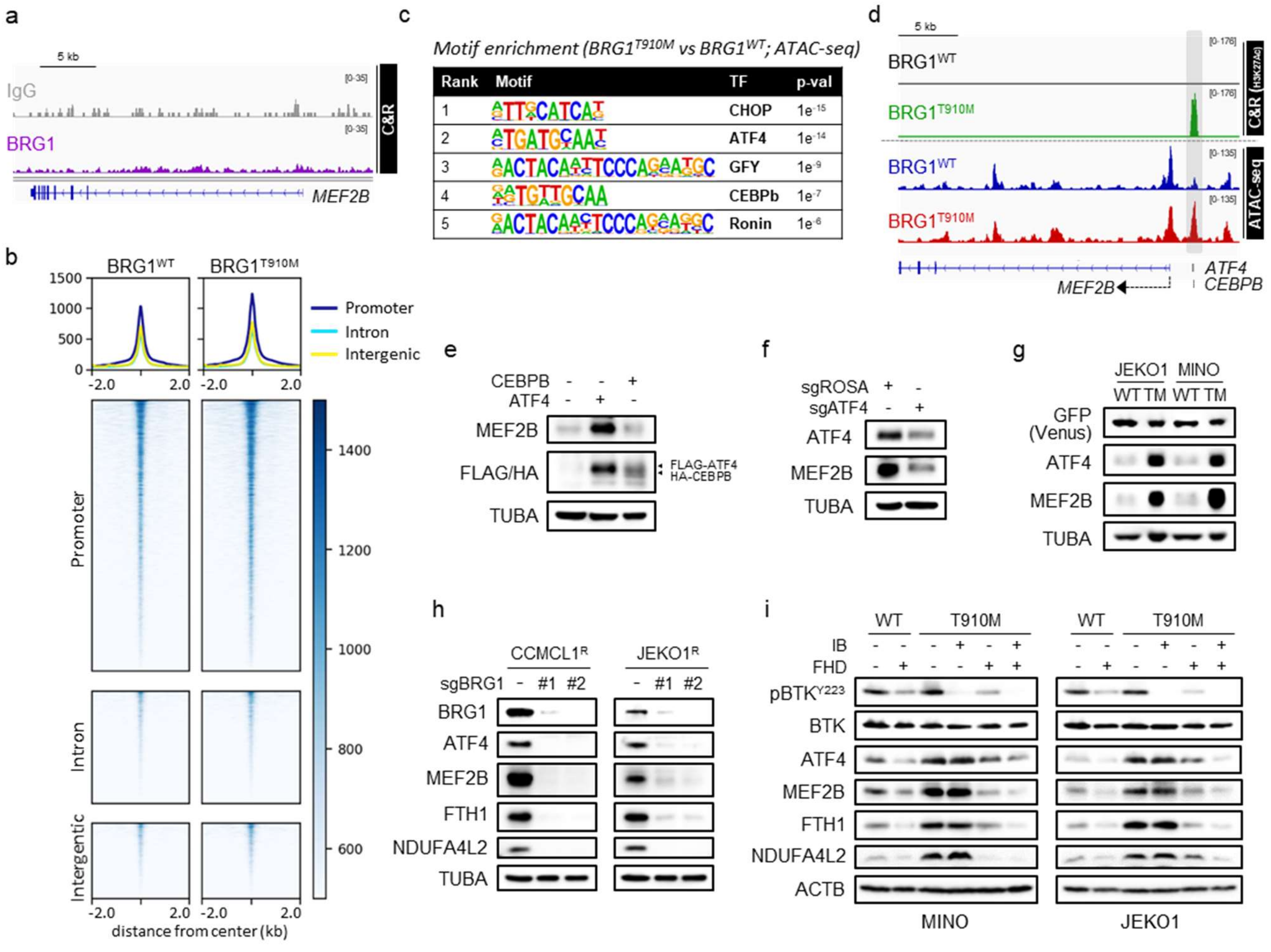

Reviewer Fig. 2. BRG1^{mut} induces ATF4 to drive transcriptional upregulation of MEF2B. (a) IGV track of anti-IgG and anti-GFP epitope-tagged BRG1 Cut&Run at MEF2B locus. (b) Characterization of ATAC-seq peaks across promoter, intron, and intergenic regions of MINO^{WT} and MINO^{T910M} cells. (c) HOMER motif enrichment analysis of differentially accessible regions (macs2, $p<0.0001$) between MINO^{T910M} and ^{WT} cells. (d) IGV tracks of ATAC-seq and H3K27ac Cut&Run in MINO^{WT} and MINO^{T910M} cells at the MEF2B locus. (e-f) Immunoblot analysis following ectopic expression of ATF4 or CEBPB in JEKO1^{WT} cells (e), ATF4 depletion in JEKO1^{T910M} cells (f). (g) Immunoblot analysis comparing BRG1^{WT} and BRG1^{T910M} MCL cells. (h) Immunoblot of CCMCL1^{R} and JEKO1^{R} cells following BRG1 depletion. (i) Immunoblot of BRG1^{WT} and BRG1^{T910M} cells treated with either IB, FHD-286, or their combinations for 24 h.

- MEF2B to Target Genes: While MEF2B Cut&Run data is presented for NDUFA4L2 and FTH1, the functional validation relies heavily on depletion/rescue and overexpression experiments. The study does not utilize, for instance, a dominant-negative MEF2B or promoter mutagenesis to definitively show that MEF2B's transcriptional activity is required for the regulation of these specific genes in this context.

: To directly test the requirement for MEF2B transcriptional activity, we generated a DNA-binding-deficient MEF2B mutant ($MEF2B^{K23R}$) that disrupts the conserved MADS domain and abolishes sequence-specific DNA binding and transcriptional function[2]. As expected, $MEF2B^{K23R}$ failed to activate a MEF2B-responsive reporter, whereas wild-type MEF2B ($MEF2B^{WT}$) significantly induced the reporter activity (Reviewer Fig. 3a).

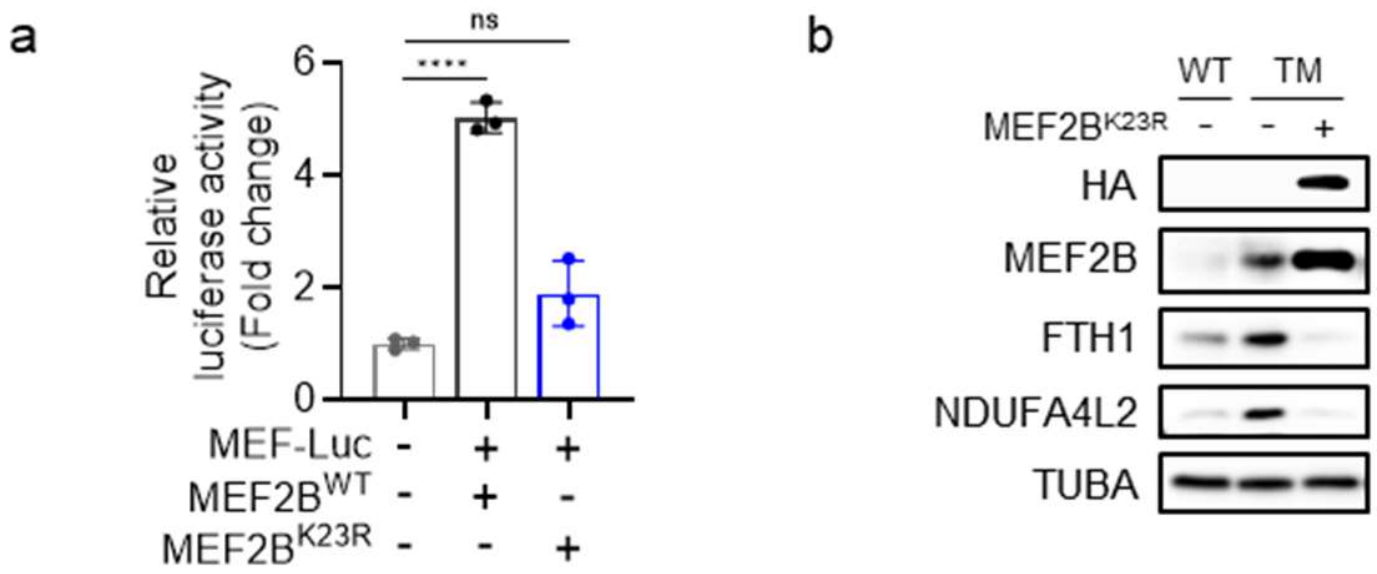

Reviewer Fig. 3. MEF2B drives transcriptional upregulation of NDUFA4L2 and FTH1. (a) Relative luciferase activity of a MEF reporter in 293T cells transiently expressing $MEF2B^{WT}$ or $MEF2B^{K23R}$ (24 h transfection, n=3) (b) Immunoblot analysis comparing $JEKO1^{WT}$, $JEKO1^{T910M}$, and $JEKO1^{T910M}$ cells expressing $MEF2B^{K23R}$.

Importantly, expression of $MEF2B^{K23R}$ in $BRG1^{T910M}$ cells reduced NDUFA4L2 and FTH1 expression, consistent with dominant-negative interference with endogenous MEF2B activity (Reviewer Fig. 3b). This result demonstrates that MEF2B DNA binding and transcriptional activity are required for BRG1-dependent regulation of NDUFA4L2 and FTH1. Together with MEF2B Cut&Run occupancy at these loci, these data support MEF2B as a direct transcriptional mediator of their regulation in this context. These data have been incorporated into Fig. S5 to provide additional support for the transcriptional regulation of NDUFA4L2 by MEF2B.

3. The authors proposed that NDUFA4L2 negatively regulates ferroptosis by reducing oxygen consumption and inhibiting mitochondrial activity. However, in the abstract, they claimed NDUFA4L2 blocks mitochondrial damage-dependent ferroptosis. It sounds controversial.

: We thank the reviewer for pointing out important clarification on the mechanistic description. Our data support a two-pronged mechanism by which NDUFA4L2 suppresses ferroptosis. By inhibiting mitochondrial Complex I and reducing cellular respiration, NDUFA4L2 (i) preemptively limits BTKi-induced mitochondrial ROS generation, and (ii) induces AMPK activation, together establishing a metabolic state that raises the threshold for lipid peroxidation and ferroptotic cell death. In this context, ferroptosis suppression reflects prevention of mitochondrial stress rather than interference with damage once it occurs.

To ensure consistency with our data, we have revised the abstract to remove the phrase “mitochondrial damage dependent ferroptosis” and to explicitly state that NDUFA4L2 suppresses ferroptosis by reducing mitochondrial activity and engaging AMPK signaling upstream of ferroptotic execution.

4. Overexpression of NDUFA4L2 inhibits oxygen consumption, which may activate AMPK signal, a negative regulator of ferroptosis. Does BTKi resistant cells have higher AMPK signal, therefore inhibiting ferroptosis?

: We appreciate this insightful suggestion, which helped clarify the functional role of NDUFA4L2 in ferroptosis resistance. As we shown in our response to comment #1, BTKi resistant BRG1-mutant MCL

cells exhibit elevated AMPK activation relative to BTKi-sensitive counterparts (Reviewer Fig. 1c, d), consistent with NDUFA4L2-driven suppression of mitochondrial respiration. Functionally, AMPK activation contributes to ferroptosis resistance: pharmacologic AMPK activation (metformin) suppressed BTKi-induced lipid peroxidation and cell death (Reviewer Fig. 1e, f), whereas selective AMPK inhibition (BAY-3827) enhanced ferroptosis in BTKi resistant BRG1-mutant cells (Reviewer Fig. 1g-j). These findings support a model in which elevated AMPK signaling downstream of NDUFA4L2 reduces ferroptotic vulnerability in BTKi-resistant MCL.

5. The authors often overstate the novelty or specificity of their findings. For example, the link between BTK inhibition, mitochondrial dysfunction, and ferroptosis has been explored in other contexts (e.g., recent studies on BTKi and ferroptosis PMID: 36693553), yet the manuscript does not adequately contextualize their findings relative to the existing literature.

: We agree that prior studies have linked BTK inhibition to mitochondrial dysfunction and ferroptosis in other contexts. We have revised the discussion section to explicitly acknowledge prior works demonstrating BTKi-induced ferroptotic stress, including PMID: 36693553, and to clarify how our findings relate to this literatures. Our study does not claim novelty in ferroptosis induction by BTKi; rather, our contribution is identifying a chromatin-driven, resistance-specific mechanism that suppresses this vulnerability in BTKi-refractory MCL. We demonstrate that aberrant BRG1-dependent transcriptional reprogramming enforces a low-ROS, AMPK-high metabolic state that raises the threshold for BTKi-induced ferroptosis, establishing epigenetic regulation as a key determinant of ferroptotic escape and a targetable resistance mechanism.

Minor questions:

1. The authors should provide western blot verification for all genetic modified cells in this manuscript. The data are not limited as following: Figure S1B, CRISPB-Cas9 mediated KO of BTK should be verified by western blot. Figure S2A. The expression of BRG1wt and BRG1K785R in MINO or JEKO cells should be verified by western blot. Figure CRISPB-Cas9 mediated KO of MEF2B and rescued cells should be verified by western blot.

: Western blot validation for all genetically modified cell lines is included in the manuscript. CRISPR-Cas9-mediated BTK knockout is verified by immunoblotting in Fig. S1b; expression of $BRG1^{WT}$ and $BRG1^{K785R}$ in MINO and JEKO1 cells is confirmed in Fig. S2a; and CRISPR-Cas9-mediated MEF2B and NDUFA4L2 knockout and rescue are validated by immunoblotting in Fig. S4b, S4g, S5c and S5f.

2.Figure 1A and B: It was reported that necrostatin-1 has ferroptosis inhibition activity. necrostatin-1S is a more specific necroptosis inhibitor.

: We agree that necrostatin-1 has reported off-target ferroptosis-inhibitory activity. Importantly, in our experiments, necrostatin-2 did not rescue BTKi-induced cell death (Reviewer Fig. 4), indicating that necroptosis is not the dominant death pathway in this setting (Fig. 1a). We have replaced the data with new plots.

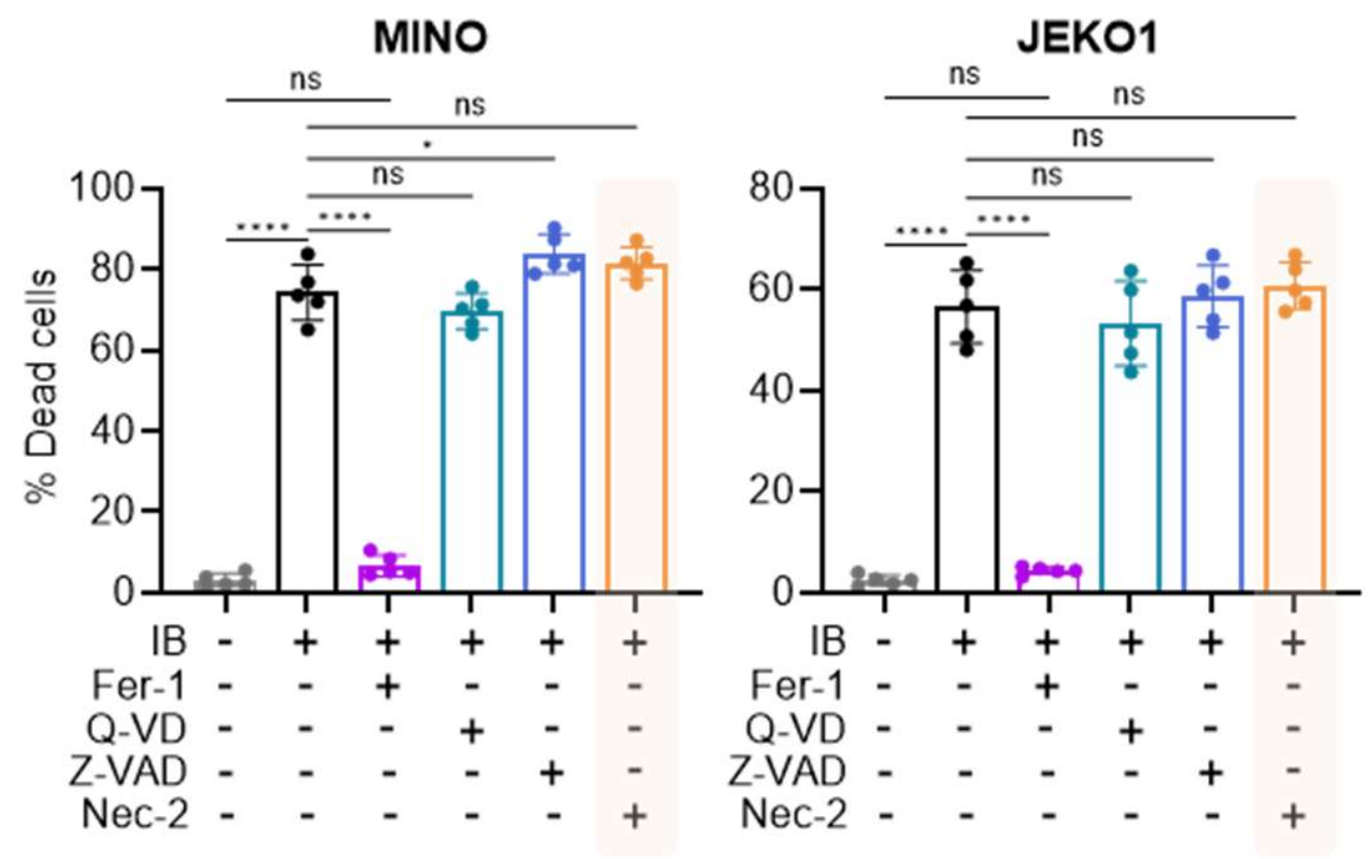

Reviewer Fig. 4. Ferroptosis suppression underlies BTK inhibitor resistance. Effect of cell death inhibitors (Fer-1, 2 μM; Q-VD-Oph, 10 μM; Z-VAD-FMK, 10 μM; Necrostatin-2, 10 μM) on IB-induced (96 h) cell death in MINO and JEKO1 cells.

Reviewer #2 - MCL (Remarks to the Author):

Resistance to BTK inhibitors (BTKi) in MCL remains a critical therapeutic challenge. The study by Hwang and colleagues suggests that ferroptosis suppression is a major mechanism driving BTKi resistance. They identify aberrant BRG1 as a key regulator of this process, orchestrating transcriptional programs that inhibit ferroptosis pathways independently of BTK signaling. Furthermore, they show that BRG1 mutations may increase cellular reliance on BRG1 activity, enabling alternative resistance mechanisms. Mechanistically, MEF2B mediates BRG1-driven ferroptosis suppression through direct activation of NDUFA4L2, which functions as a pivotal downstream effector of the BRG1T910M-MEF2B axis. NDUFA4L2 protects MCL cells from BTKi-induced mitochondrial damage by redirecting metabolism away from oxidative phosphorylation, thereby reducing reactive oxygen species and iron release. Although these findings provide a strong preclinical rationale for therapeutic strategies combining BTKi with BRG1 inhibition (FHD-286) to overcome resistance in MCL, important limitations should be addressed, particularly the specificity of the FHD-286 and the limited validation in primary MCL samples.

: We thank this reviewer for requesting to clarify the specificity of FHD-286 and validation in primary MCL cells.

Major Comments:

1. Figure 1. The data presented are from cell lines without clinical correlation. The mechanistic link between ferroptosis suppression and BTKi resistance should be validated in primary MCL samples. Figure 1G. GSEA shows enrichment of ferroptosis suppressor genes in resistant cells (JEKO and patients), However, functional validation (e.g., Fe^{2+}, ROS or Lipid peroxidation levels or death upon b-Me, NAC) is not described in the excerpt.

: As suggested by the reviewer, we performed functional ferroptosis analyses in primary MCL samples stratified by IB sensitivity. Using lipid peroxidation as a direct ferroptotic readout, we found that IB failed to induce lipid peroxidation in resistant primary MCL cells, whereas sensitive samples showed a robust increase (Reviewer Fig. 5a). In contrast, the BRM/BRG1 inhibitor FHD-286 markedly increased lipid peroxidation in primary MCL cells, and combined treatment with FHD-286 and IB produced a synergistic effect. These results provide direct functional validation that ferroptosis suppression characterizes BTKi-resistant MCL and link chromatin remodeling-mediated ferroptosis regulation to therapeutic response in patient-derived samples, consistent with the enrichment signatures shown in Figure 1h. These primary MCL validation is now presented as Figure 8f.

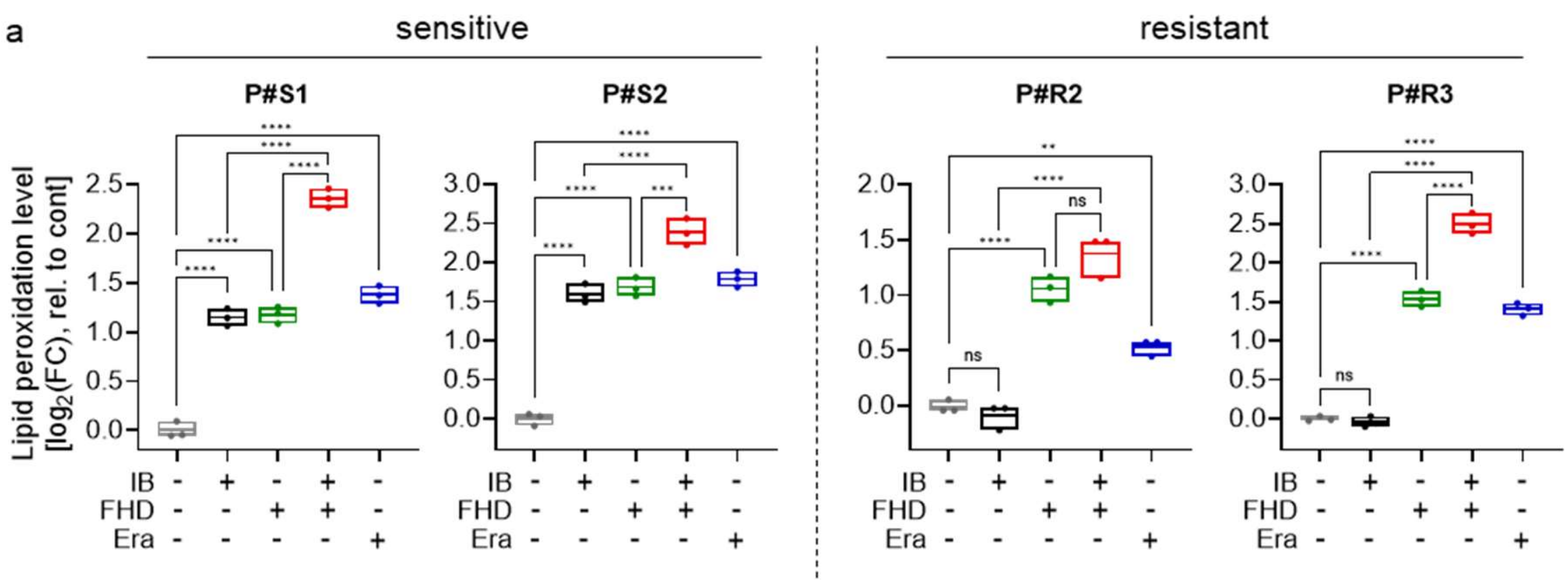

Reviewer Fig. 5. Combined BTK and BRG1 inhibition enhances lipid peroxidation in primary MCL cells. (a) Changes in lipid peroxidation upon IB, FHD-286 treatment or their combination in IB-sensitive and –resistant primary MCL cells (48 h, n=3).

2. Figure 2G-L. Authors should demonstrate effective and specific knockout of BGR1 in Figure 2G-H. Are de levels of BRG1 similar in IB resistant than in sensitive cell lines (compared BRG1 levels in CCMCL1R and JEKO1R vs CCMCL1S and JEKO1S cells, respectively). Cell death in sgBRG1 is expected, as BRG1

has essential function for living cells. The innovative finding will be to show that cell death upon BRG1 knockout is due to ferroptosis. Comparison of experiments shown in Fig 2I should be done in sgBrg1 cells and compared them with control and mutant cells.

: First, we have demonstrated effective depletion of BRG1 in cells used throughout Figure 2G-H (Reviewer Fig. 6a), which strengthens our finding that BTKi-resistant MCL cells are highly dependent on BRG1. Data is now included as Figure S2g.

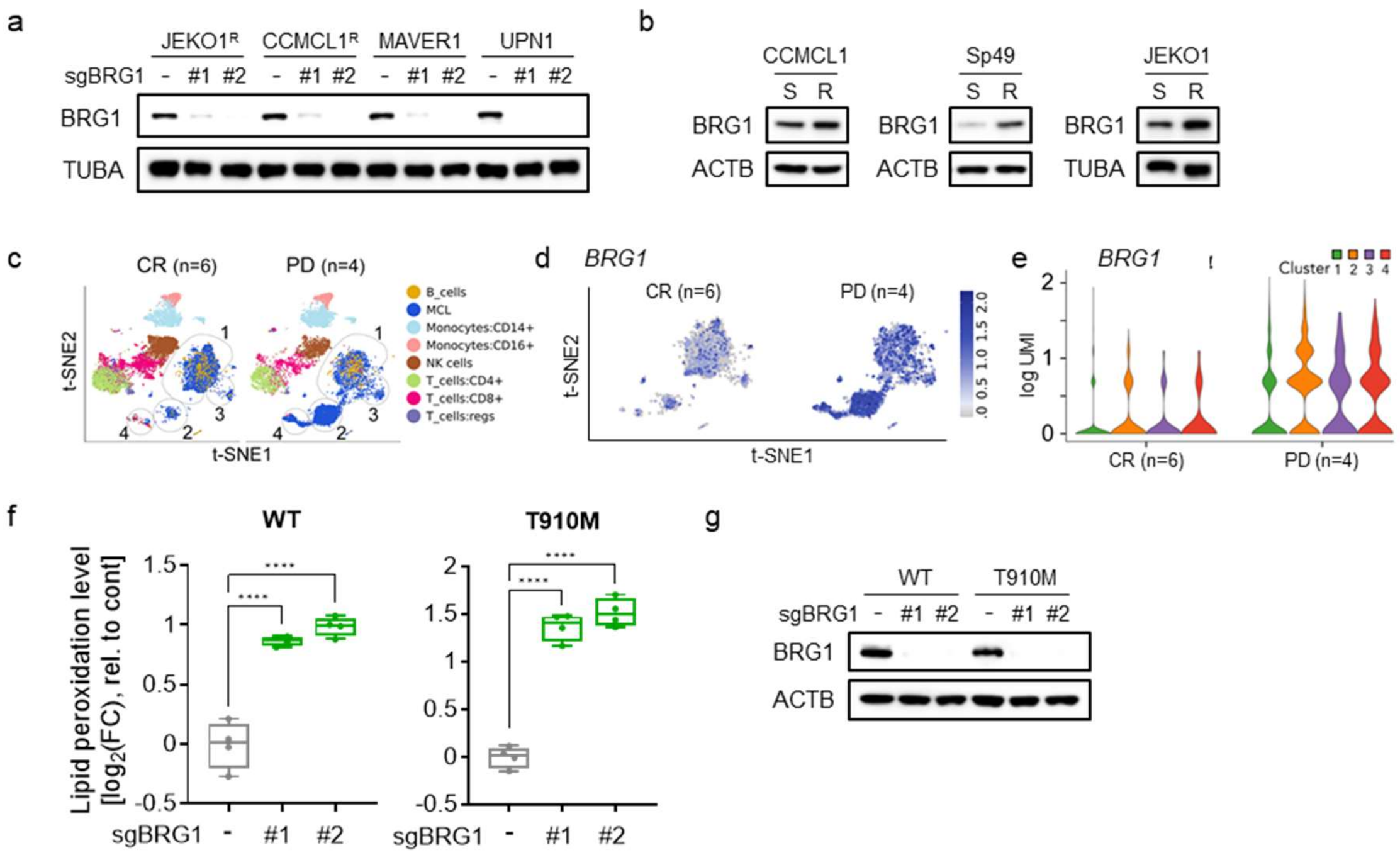

Reviewer Fig. 6. Elevated BRG1 suppresses lipid peroxidation in BTKi-resistant MCL models. (a) Immunoblot of BRG1-depleted CCMCL1^{R}, JEKO1^{R}, MAVER1 and UPN1 cells (4-day post-sgRNA expression). (b) Immunoblot comparing BRG1 expression in isogenic BTKi^{S} (S) and BTKi^{R} (R) pairs of CCMCL1, Sp49, and JEKO1 cells. (c) *t*-distributed stochastic neighbor embedding (*t*-SNE) analysis of unsupervised clustering of single cell RNA sequencing (scRNA-seq) data from PBMCs of MCL patients with complete response (CR, 6 specimens) or progressive disease (PD, 4 specimens) in the PALIBR clinical study[3]. B cells (*CD19*$^{+}$/CCND1^{-}), MCL cells (*CD19*$^{+}$/*CCND1*$^{+}$), CD4^{+} T cells (*CD3D*$^{+}$/*CD4*$^{+}$), CD8^{+}T cells (*CD3D*$^{+}$/*CD8A*$^{+}$), T-regs (*CD25*$^{+}$/*FOXP3*$^{+}$), natural killer (NK) cells (*KLRC1*$^{+}$/*NCAM1*$^{+}$/*GNLY*$^{+}$), CD14^{+} (*CD14*$^{+}$/*FCGR1A*$^{+}$) and CD16^{+} (*CD16*$^{+}$/*FCGR3A*$^{+}$) monocytes are indicated. B cells and MCL cells comprise four major transcriptionally distinct clusters (grey circle). (d-e) t-SNE analysis (d) and violin plot (e) showing BRG1 expression in MCL cells from samples shown in (c). (f) Changes in lipid peroxidation (n=4) following BRG1 depletion in MINO^{WT} and MINO^{T910M} cells (4-day post-sgRNA expression). (g) Immunoblot confirming BRG1 depletion in MINO^{WT} and MINO^{T910M} cells (4-day post-sgRNA expression).

Moreover, as suggested, we analyzed BRG1 protein expression levels in isogenic cell pairs. We observed a reproducible increase in BRG1 protein abundance in resistant cells (Reviewer Fig. 6b). This finding is consistent with elevated BRG1 transcript levels observed in ibrutinib-resistant (PD) patient samples compared with ibrutinib-sensitive (CR) samples in single-cell RNA-seq datasets (Reviewer Fig. 6c-e). Functionally, genetic suppression of BRG1 (sgBRG1) resulted in increased lipid peroxidation (Reviewer Fig. 6f, 6g) and subsequent cell death (Fig. 2h), supporting a direct role for BRG1 in ferroptosis suppression in resistant cells. These data were now included as Fig. 7a-c, S2j, and S2k.

3. Resistance to IB and sensibility to FHD-286 in BRG1MUT MCL primary cases should be analyzed in comparison to BRG1WT and IB sensitive and resistant cases.

: We originally presented data comparing FHD-286 sensitivity between BRG1^{WT} and BRG1^{MUT} IB-resistant primary MCL samples (Fig. 8a and Fig. S9g). In the revised manuscript, we additionally included the response of IB-sensitive primary MCL samples to FHD-286 (Reviewer Fig. 7), which is now

incorporated into Fig. 8a. Notably, none of the IB-sensitive samples analyzed (CR, 6 specimens) harbored BRG1 mutations, precluding direct $BRG1^{WT}$ versus $BRG1^{MUT}$ comparisons in this cohort.

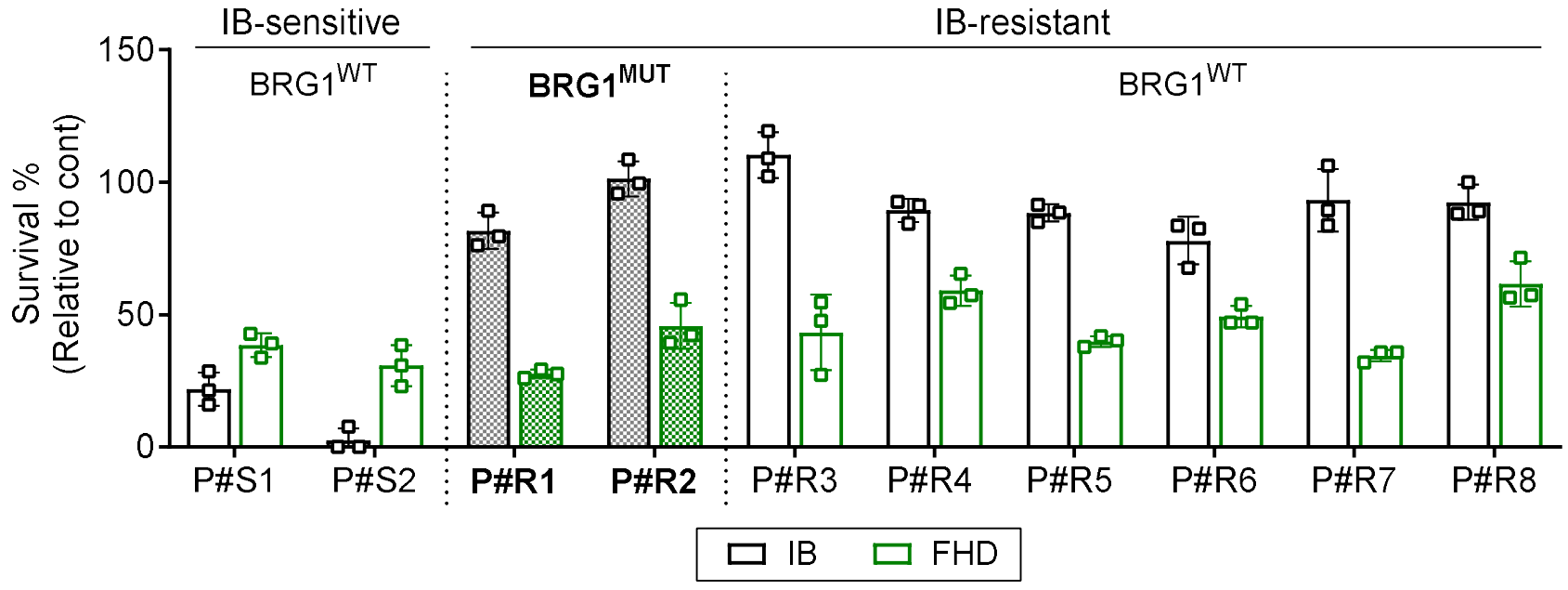

Reviewer Fig. 7. FHD-286 sensitivity in primary MCL samples stratified by BRG1 mutation and IB response. Relative cell survival of primary MCL cultures from IB-sensitive or -resistant patients following treatment with IB or FHD-286 (96 h, n=3).

4. FHD-286 is described as a dual BRG1/BRM inhibitor. The manuscript does not fully address this dual activity, nor does it demonstrate that the observed physiological effects are specifically mediated through BRG1. This point is important because ATAC-seq analysis of BCR-related genes shows a less reduction upon FHD-286 treatment compared to sgBRG1. Additional evidence confirming that the effects of FHD-286 are primarily due to BRG1 inhibition would help rule out potential off-target contributions.

: We note that the chromatin accessibility changes induced by FHD-286 are not identical to those observed upon sgBRG1-mediated depletion. These differences are mechanistically expected, as FHD-286 functions as a catalytic inhibitor of BRG1/BRM ATPase activity, whereas sgBRG1 results in complete structural loss of BRG1-containing SWI/SNF complexes. Consequently, BRG1 remains chromatin-associated in the presence of FHD-286 but is absent upon genetic depletion, leading to quantitatively and qualitatively distinct chromatin accessibility outcomes (Please also see Reviewer Fig. 9).

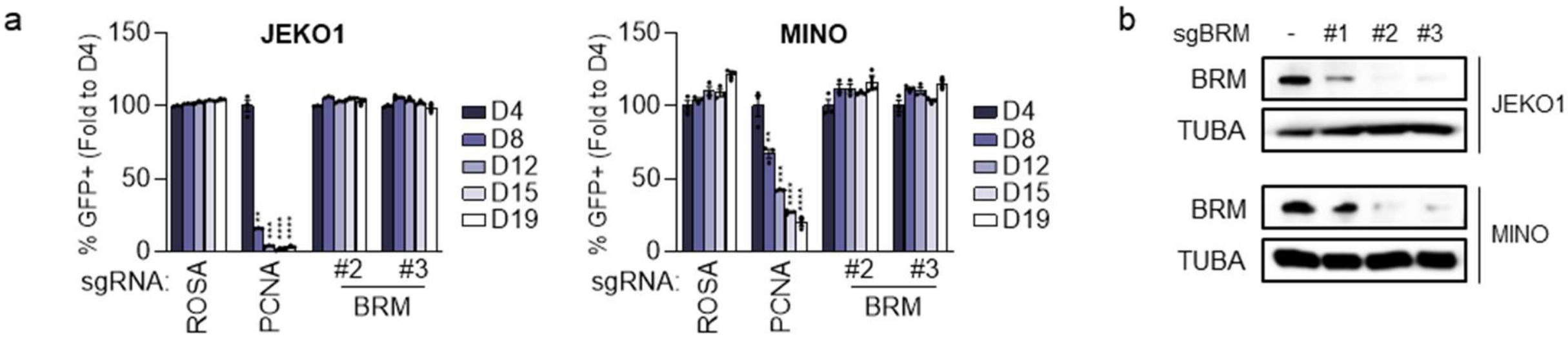

Reviewer Fig. 8. BRM depletion does not impair MCL cell viability. (a) Competition-based proliferation assays with BRM-targeting sgRNAs in Cas9-transduced JEKO1 and MINO cells (n=3). sgROSA and sgPCNA was used as a negative and positive control, respectively. (b) Immunoblot verification of BRM depletion in JEKO1 and MINO cells (4-day post-sgRNA expression).

In response to the reviewer's request for additional evidence addressing the dual BRG1/BRM activity of FHD-286, we performed sgBRM-based genetic dropout experiments (Reviewer Fig. 8a, 8b), which have now been incorporated into the revised manuscript as Fig. S2h, S2i. Notably, BRM depletion alone did not compromise MCL cell viability, in contrast to the pronounced effects observed upon BRG1 suppression, indicating that BRM is not independently required for MCL survival. Together, these data support the conclusion that the dominant physiological effects of FHD-286 in MCL are mediated primarily through BRG1 inhibition.

5. Figure 3A. To demonstrate that FHD-286 is directly inhibiting BRG1 and its transcriptional program, authors should compared, by Venny, FHD-286 and BGR1 specific DEGs, to discard of target effects. Moreover BGR1 Cut&Run experiments in FHD-286 treated and sgBRG1 cells should be compared to control cells.

: We appreciate this point and initially performed RNA-seq comparing sgBRG1 and FHD-286–treated cells. However, acute BRG1 depletion predominantly induced DNA damage and stress response pathways, limiting its utility for defining the BRG1-dependent transcriptional program. To more directly assess on-target effects of pharmacologic inhibition while minimizing confounding responses, we revised our analysis strategy. Specifically, we intersected PRO-seq DEGs from FHD-286–treated versus control cells with $BRG1^{WT}$ Cut&Run peaks defined relative to IgG control, thereby restricting analysis to genes with direct BRG1 occupancy (Reviewer Fig. 9a). This integrated approach identifies putative direct BRG1 targets modulated by FHD-286. Pathway enrichment analysis of this refined gene set recapitulated our original findings, with B-cell receptor (BCR) signaling as the top enriched pathway. These results are now incorporated into Fig. 3a and 3b.

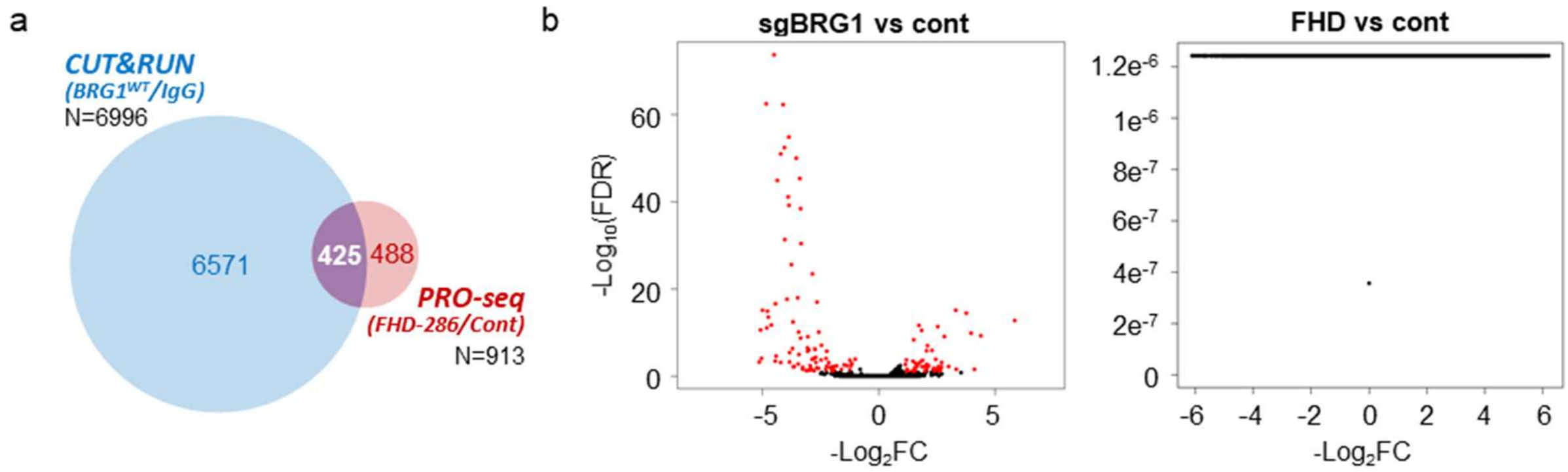

Reviewer Fig. 9. FHD-286 inhibits BRG1-dependent transcription without altering chromatin occupancy. (a) Venn diagram showing overlap between $BRG1^{WT}$ and IgG Cut&Run peaks (FDR<0.1) and PRO-seq DEGs (FHD-286 vs. control; FDR<0.1, |FC|>1.5) in MINO cells. (b) Volcano plots showing BRG1 Cut&Run signal in FHD-286-treated and sgBRG1-expressed cells relative to control cells.

To further address the reviewer's suggestion, we also compared BRG1 Cut&Run profiles in FHD-286- and sgBRG1-treated cells relative to control cells. As shown in Reviewer Fig. 9b, FHD-286 treatment did not measurably affect BRG1 chromatin occupancy, whereas BRG1 depletion led to widespread alterations in BRG1 binding across target loci. These results indicate that FHD-286 suppresses BRG1-dependent transcriptional activity without displacing BRG1 from chromatin, in contrast to sgBRG1-mediated depletion.

6. Fig 4A. Is MEF2B directly regulated by BRG1? Although increased MEF2B expression was observed in BRG1T910M vs. BRG1WT cells (Fig.4B-D), Fig.4D seems to indicate that MEF2B degradation is compromise in BRG1T910M cells. To demonstrate BRG1T910M is directly regulating MEF2B transcription in MCL cells, Cut&Run experiments in respectively cell lines, comparing binding of BRG1T910M vs. BRG1WT proteins to MEF2B regulatory regions should be shown. What are the mRNA and protein MEF2B levels in IB resistant vs. sensitive MCL cell lines and cases (CCMCL1R, JEKO1R and patientsR vs. CCMCL1S, JEKO1S patientss cells). Incorporating these data could strengthen authors's claim, proposing that BRG1 contributes to BTKi resistance through direct transcriptional regulation of BCR-related and -not related genes (MEF2B) by confirming BRG1's direct involvement.

: Please see our response to reviewer#1 point#2: our data shows $BRG1^{T910M}$ indirectly upregulates MEF2B. Our ongoing investigation shows it is primarily induced by ATF4. Our Cut&Run data preclude direct transcriptional regulation of MEF2B by BRG1 as there is no detectable binding where BRG^{T910M}-induced accessible chromatin regions are.

As suggested by this reviewer, our analysis of isogenic pairs of S vs. R CCMCL1 and JEKO1) indeed shows increased MEF2B expression levels (Reviewer Fig. 10). Although not a direct regulatory axis, we find faithful response of MEF2B expression upon aberrant BRG1 activity. This data now has been incorporated into the manuscript as Fig. 7e.

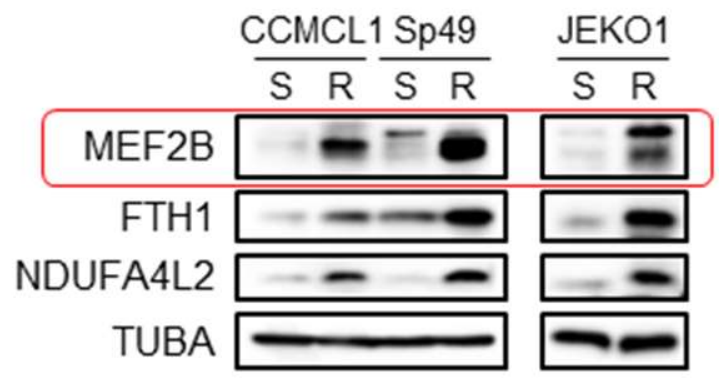

Reviewer Fig. 10. MEF2B-NDUFA4L2/FTH1 axis is upregulated in BTKi resistant cells. Immunoblot comparing isogenic BTKi^S (S) and BTKi^R (R) pairs of CCMCL1, Sp49, and JEKO1 cells

7. The manuscript suggests that BRG1T910M activates a BCR-independent pathway via MEF2B, leading to upregulation of NDUFA4L2 and FTH1 and consequently BRG1 inhibition by FHD-286 treatments would induce cell death thought increasing ROS and Fe2+ levels inducing ferroptosis cell death. It would be informative to determine whether the MEF2B, NDUFA4L2 and FTH1 are also downregulated in FHD-286 treated vs control and how its levels are in sgBRG1 vs control MCL cell lines or in IB resistant vs. sensitive MCL cell lines and cases (CCMCL1R, JEKO1R and patientsR vs. CCMCL1S, JEKO1S patientss cells). This comparison could provide additional mechanistic insight.

: To address this point, we examined the expression of MEF2B, NDUFA4L2, and FTH1 following both genetic and pharmacologic perturbation of BRG1 in MCL models. Notably, baseline level of all three genes was elevated in BTKi-resistant cell line models and IB-resistant primary cultures compared to their sensitive counterparts, supporting activation of this axis in resistant disease (Reviewer Fig. 10, 11a).

Genetic depletion of BRG1 in BTKi-resistant cell lines (CCMCL1^R and JEKO1^R) led to downregulation of all three genes (Reviewer Fig. 11b). Similarly, pharmacologic inhibition with FHD-286 also resulted in marked downregulation of this axis in both BTKi^S and ^{R} cell lines (CCMCL1^S and CCMCL1^R) as well as in IB-resistant patient-derived MCL cultures (Fig. 8B, Reviewer Fig. 11c). Together, these findings support that MEF2B, NDUFA4L2, and FTH1 are coordinately regulated downstream of BRG1 and are suppressed upon BRG1 inhibition.

Due to the technical limitations associated with CRISPR/Cas9-mediated gene depletion in primary MCL cells, we assessed this axis in primary samples using FHD-286 treatment, which yielded results consistent with those observed in cell line models. These findings have now been incorporated into Fig. S7m, 7f, and 7g of the revised manuscript.

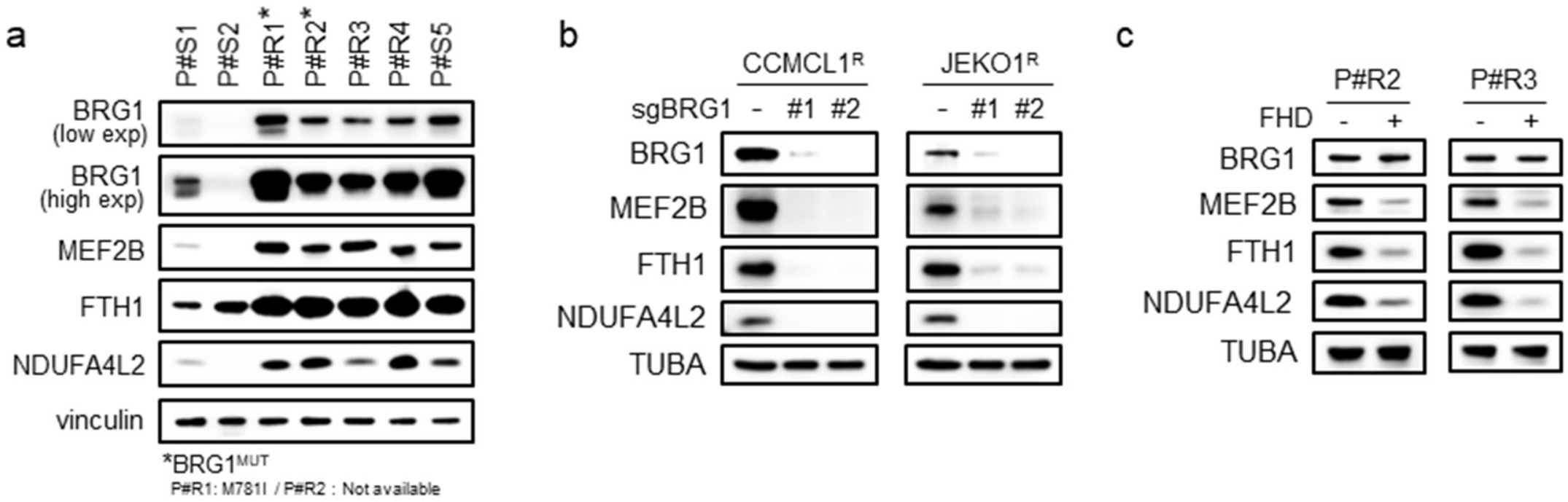

Reviewer Fig. 11. BRG1 regulates the MEF2B–NDUFA4L2/FTH1 transcriptional axis in MCL. (a) Immunoblot of IB-resistant MCL cultures following FHD-286 treatment (24 h). (b) Immunoblot of BRG1-depleted CCMCL1^R and JEKO1^R cells (4-day post-sgRNA expression). (c) Immunoblot of IB-resistant MCL cultures following FHD-286 treatment (24 h).

8. MEF2B, NDUFA4L2 and FTH1 expression is increased in BRG1T910M compared to BRG1WT. MEF2B is directly regulating NDUFA4L2 and FTH1 expression. Is BRG1 also involved as cofactor in the regulation of these two genes? ATAC-seq or Cut&Run at NDUFA4L2 and FTH1 regulatory regions in BRG1T910M compared to BRG1WT could provide additional mechanistic insight.

: Our anti-GFP Cut&Run analysis did not reveal detectable occupancy at the regulatory regions of NDUFA4L2 and FTH1, indicating that BRG1 does not directly bind to these loci (Reviewer Fig. 12a). However, ATAC-seq analyses showed that these regions are more accessible in BRG1^{T910M} cells compared to BRG1^{WT} cells, and become less accessible upon BRG1 depletion, indicating BRG1-dependent regulation of chromatin state at these regions (Reviewer Fig. 12b). Importantly, these regions overlapped with MEF2B bound regions identified by Cut&Run (Reviewer Fig. 12b), supporting an indirect role for BRG1 in the regulation of these MEF2B-bound genes.

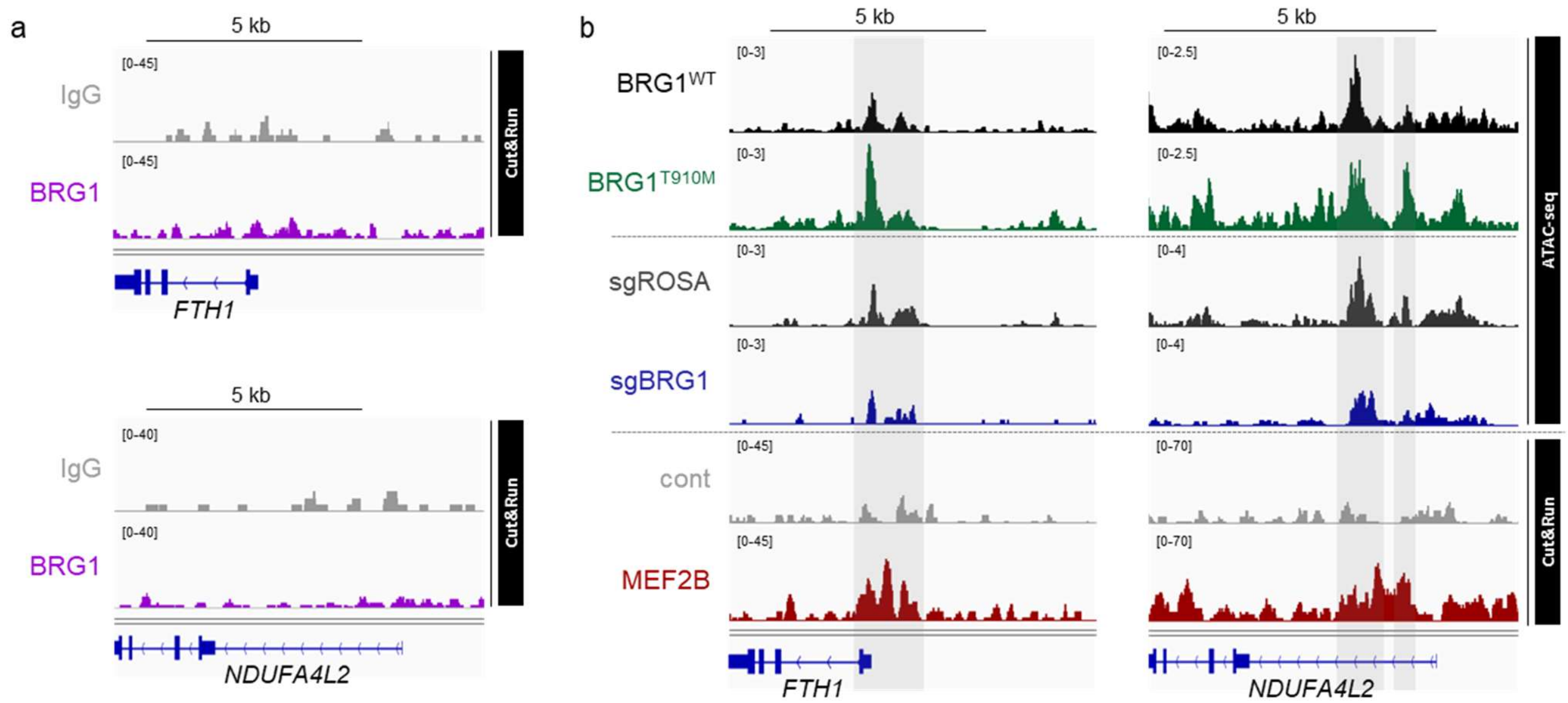

Reviewer Fig. 12. BRG1 indirectly regulates NDUFA4L2 and FTH1. (a) IGV track of anti-IgG and anti-GFP Cut&Run in Venus-tagged MINO^{WT} cells at FTH1, NDUFA4L2 loci. (b) IGV track of ATAC-seq (BRG1^{WT} vs. BRG1^{T910M} MINO cells; sgROSA vs. sgBRG1 CCMCL1Δp53 cells) and HA-MEF2B Cut&Run at FTH1, NDUFA4L2 loci.

9. The degree of BTKi resistance in BRG1T910M compared to BRG1WT cells is not explicitly compared. Demonstrating whether resistance is significantly higher in BRG1T910M compared to BRG1WT cells, potentially due to increased MEF2B, NDUFA4L2, and FTH1 expression, would add clarity to the functional impact of this mutation.

: We agree that an explicit comparison improves clarity. Dose response analyses demonstrate that BRG1^{T910M} cells exhibit markedly greater resistance than BRG1^{WT} cells to both IB and PB, where the IC_{50} values of WT cells are in the low micromolar range (IB: 1.54 and 3.05 μM; PB: 3.85 and 4.29 μM in MINO and JEKO1, respectively),while those of T910M cells exceed 10 μM (Reviewer Fig. 13a, b). This increased resistance correlates with elevated expression of MEF2B, NDUFA4L2, and FTH1 in BRG1^{T910M} cells, consistent with enhanced suppression of ferroptosis. We have now incorporated the data in the manuscript as Fig. S2b.

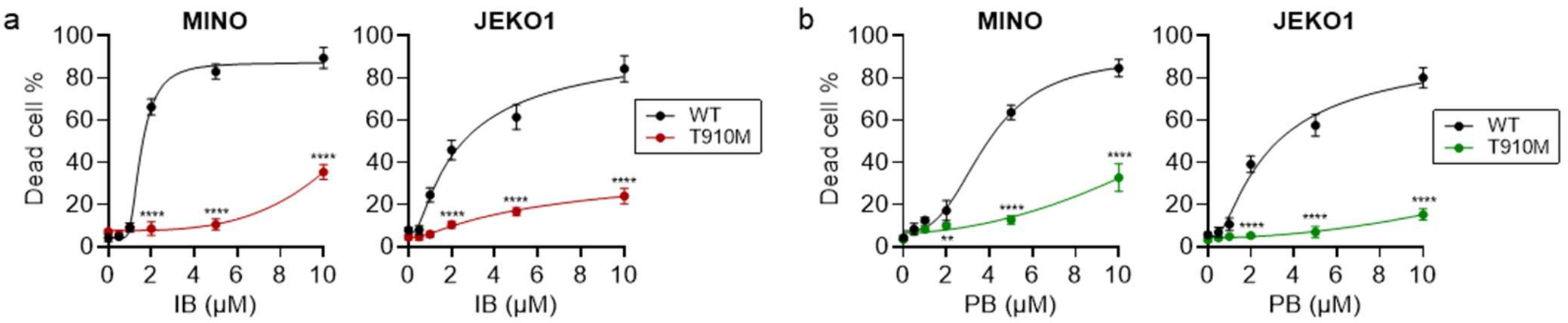

Reviewer Fig. 13. Dose-Response Analysis of BTK Inhibitor Sensitivity in BRG1^{WT} and BRG1^{T910M} Cells. (a-b) Dose-response curves of IB (a) and PB (b) in MINO and JEKO1 cells expressing BRG1^{WT} or BRG1^{T910M} (n=4. 96 h).

10. Recent studies indicate that BRG1 can act as a cofactor for SOX11 in the transcriptional activation of BCR-related and -not related genes in MCL cells (PMID: 40739087). Since the genes regulated by BRG1 overlap with those influenced by SOX11, the authors should discuss whether BRG1T910M might affect SOX11-mediated transcriptional regulation and if SOX11 it is also involved in MEF2B/NDUFA4L2/FTH1 axis inducing ferroptosis suppression.

: We agree that BRG1-SOX11 cooperation is an important consideration. To address this, we examined SOX11 expression in $BRG1^{WT}$ and $BRG1^{T910M}$ cells and assessed the impact of SOX11 depletion on the MEF2B-NDUFA4L2/FTH1 axis (Reviewer Fig. 14). SOX11 expression level did not show noticeable differences between $BRG1^{WT}$ and $BRG1^{T910M}$ cells. Moreover, CRISPR-mediated SOX11 depletion in both cells did not reduce MEF2B, NDUFA4L2, or FTH1 expression, indicating that activation of this axis is SOX11-independent. These data suggest that, while BRG1 and SOX11 cooperate in regulating BCR-associated transcriptional programs, the BRG1-MEF2B-NDUFA4L2/FTH1 pathway mediating ferroptosis suppression operates independently of SOX11.

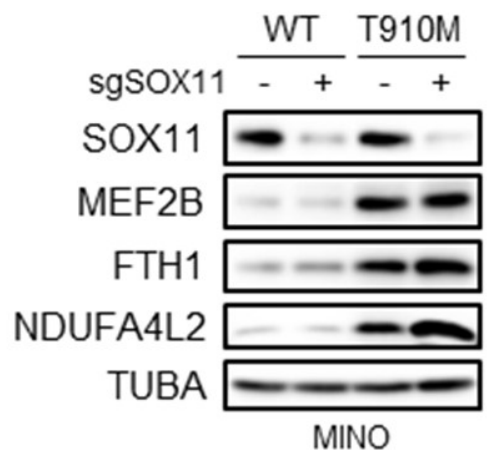

Reviewer Fig. 14. MEF2B-NDUFA4L2/FTH1 Axis Activation Is Independent of SOX11. Immunoblot of SOX11-depleted $MINO^{WT}$ and $MINO^{T910M}$ cells.

11. Figure 7A. The manuscript should clarify whether the observed differences in IC50 values among MCL WT cell lines are associated with varying BRG1 expression levels. This would help contextualize the variability in drug sensitivity.

: As suggested, we measured the expression levels of BRG1 across cell lines with different IC_{50} values for FHD-286. Across MCL $BRG1^{WT}$ cell lines, we did not observe a clear correlation between BRG1 expression levels and differences in IC_{50} values for FHD-286. While relatively higher BRG1 expression was noted in some resistant models compared to more sensitive ones (Reviewer Fig. 15a), this trend did not correspond to a proportional change in drug sensitivity (Reviewer Fig. 15b). These findings suggest that BRG1 expression alone does not account for the variability in FHD-286 response. Results were included in the revised manuscript as Fig. S8c.

a

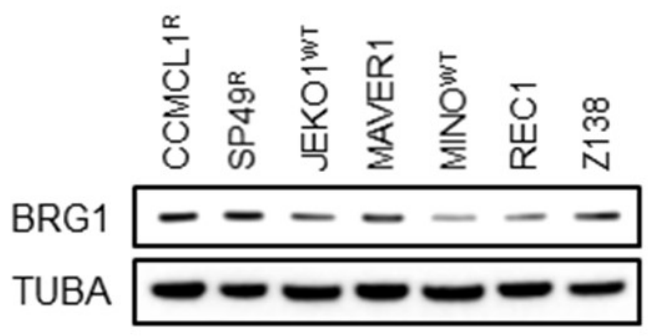

b

Cell line	IC_{50} (nM)
$CCMCL1^{R}$	6.92±0.75
$SP49^{R}$	9.29±0.63
$JEKO1^{WT}$	26.9±6.4
$JEKO1^{T910M}$	2.32±0.5
MAVER1	15.4±3.9
$MINO^{WT}$	19.4±1.8
$MINO^{T910M}$	1.13±0.55
REC1	3.75±0.45
Z138	10.4±1.5

Reviewer Fig. 15. Comparison of BRG1 Expression and FHD-286 Sensitivity Across MCL WT Cell Lines. (a-b) Immunoblot analysis of BRG1 protein expression level in MCL cell lines (a), and their IC_{50} value of FHD-286 (b; 96 h, n=3).

12. The data presented are from cell lines and mouse models without clinical correlation. Some findings should be validated in primary MCL cases, (e.g. the mechanistic link between ferroptosis suppression and BTKi resistance, MEF2B, NDUFA4L2, and FTH1 expression levels comparing BTKi-sensitive vs. -resistant samples and/or BRG1T910M versus BRG1WT genotypes). Such validation would significantly enhance the translational relevance of the study.

: To assess the clinical relevance of the BRG1-driven transcriptional program identified above, we analyzed single-cell RNA-seq data from MCL patients with complete response (CR, 6 specimens) or progressive disease (PD, 4 specimens) following BTKi therapy (Reviewer Fig. 16a). Across malignant MCL cells, BRG1 expression was significantly elevated in PD compared with CR samples (Reviewer Fig. 16b, 16c). Importantly, fraction of cells co-expressing BRG1 with MEF2B, NDUFA4L2, and FTH1 was higher in PD (Reviewer Fig. 16d), consistent with activation of a coordinated transcriptional program in resistant disease. We further validated increased protein expression of pathway effectors in PD versus CR primary samples (Reviewer Fig. 11a). These data support a mechanistic model in which BRG1-MEF2B-NDUFA4L2 signaling suppresses mitochondrial respiration and ferroptosis, providing clinical rationale for targeting BRG1 to restore ferroptotic sensitivity in BTKi-resistant MCL. These new data are now presented under Fig. 7 and Fig. S7m.

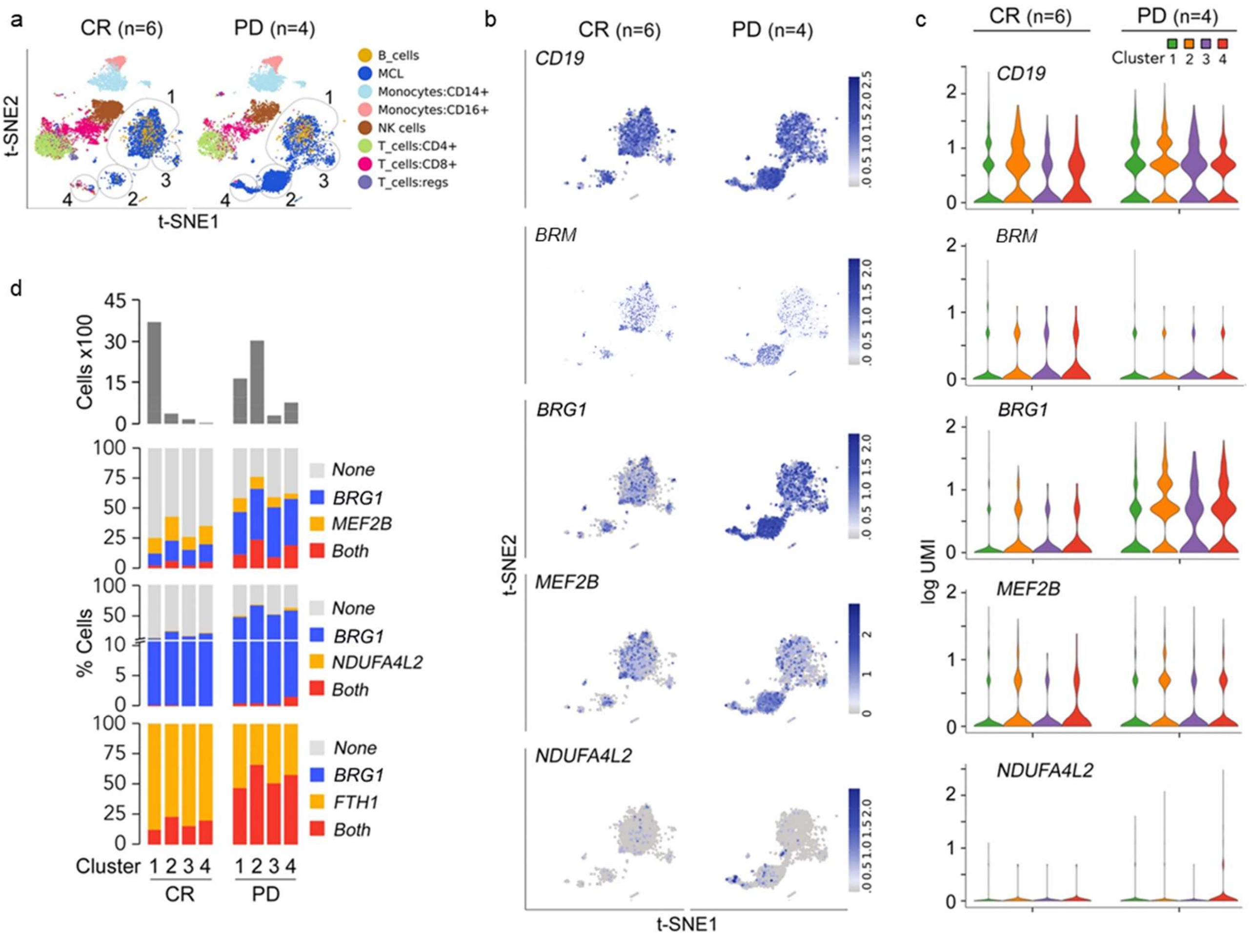

Reviewer Fig. 16. BRG1-MEF2B-NDUFA4L2/FTH1 axis is associated with therapy resistance in MCL. (a) *t*-distributed stochastic neighbor embedding (*t*-SNE) analysis of unsupervised clustering of single cell RNA sequencing (scRNA-seq) of PBMCs from MCL patients with subsequent complete response (CR, 6 specimens) or progressive disease (PD, 4 specimens) in the PALIBR clinical study[4]. B cells (*CD19*$^+$/CCND1$^-$), MCL cells (*CD19*$^+$/*CCND1*$^+$), CD4$^+$ T cells (*CD3D*$^+$/*CD4*$^+$), CD8$^+$T cells (*CD3D*$^+$/*CD8A*$^+$), T-regs (*CD25*$^+$/*FOXP3*$^+$), natural killer (NK) cells (*KLRC1*$^+$/*NCAM1*$^+$/*GNLY*$^+$), CD14$^+$ (*CD14*$^+$/*FCGR1A*$^+$) and CD16$^+$ (*CD16*$^+$/*FCGR3A*$^+$) monocytes are indicated. B cells and MCL cells comprise 4 major transcriptionally distinct clusters (grey circle). (b-c) t-SNE analysis (b) and violin plot (c) of expression of indicated genes in MCL cells from samples as in (a). (d) Total number of MCL cells (top) and percentage of expressing cells (bottom) in each cluster of MCL cells.

13. Treatments with PB and FHD are done in IB resistant and BRG1T910M cell lines but not in control and BRG1WT cell lines or patients cells. Due that these drugs also affects BCR-pathway, effects (ROS,

Lipid peroxidation and Fe2+ levels and cell death) upon treatments should be compared between BRG1T910M and BRG1WT cell lines or patients cells. Due to the high heterogeneity between MCL cells lines, cell death in Figure 7F should be done also in JEKO1R vs JEKO1S and patients R vs patientsS cells.

: To address the reviewer's point, we expanded our analyses to include $BRG1^{WT}$ along with $BRG1^{T910M}$ cells, as well as IB-sensitive and –resistant primary MCL cultures. We confirmed that BTKi treatment (IB or PB) induced lipid peroxidation in $BRG1^{WT}$ cells but not in $BRG1^{T910M}$ cells, consistent with an impaired ferroptotic response in the mutant context (Reviewer Fig. 17a). Co-treatment with FHD-286 induced robust lipid peroxidation across both genotypes, exceeding the levels observed with single-agent treatment (Reviewer Fig. 17a, Fig. 8c). This pattern was consistently recapitulated in IB-sensitive and -resistant primary MCL cultures (Reviewer Fig. 5a).

Correspondingly, combination treatment led to a significant increase in cell death compared to single-agent treatments across all tested in vitro and ex vivo models (Reviewer Fig. 17b, c, Fig. 8d, e, S9e, g). Importantly, in primary cultures, the combination effect was synergistic, with combination index (CI) values ranging from 0.18 to 0.58 (Fig. 8g). These new data are now presented in Fig. 8 and Fig. S9.

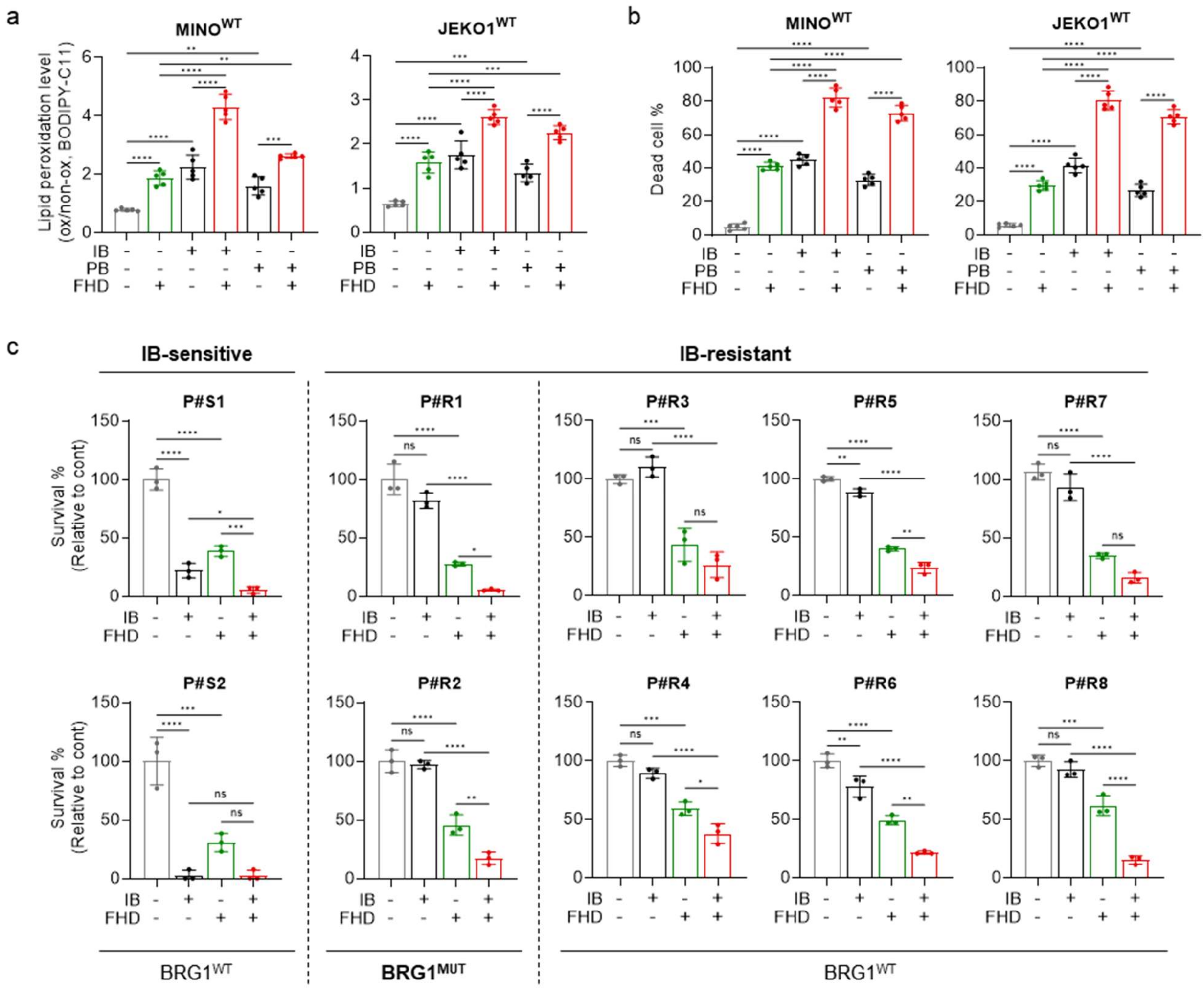

Reviewer Fig. 17. Combination of FHD-286 and BTKi Promotes Ferroptosis and Cell Death in MCL. (a) Changes in lipid peroxidation (48 h, n=5) following IB, PB, FHD-286 treatment or their combination in $MINO^{WT}$ and $JEKO1^{WT}$ cells. (b) Cell death effect (72 h) of FHD-286 and IB or PB combination in $MINO^{WT}$ and $JEKO1^{WT}$ cells. (c) Relative cell survival rate following treatment with IB (5 μM), FHD-286 (30 nM), or their combinations in primary MCL cultures (96 h, n=3).

14. Figure 7I. Proteins involved in BCR and ferroptosis suppression should be analyzed by IHC.

: We appreciate this suggestion and performed immunohistochemical (IHC) analysis on tumor tissues from the indicated treatment groups. We found that SPI1, a master transcription factor regulating BCR signaling[5], is strongly responsive to FHD-286 treatment, supporting its use as a surrogate marker of BCR pathway activity in vivo (Reviewer Fig. 18). Consistent with our in vitro findings, expression of NDUFA4L2, a ferroptosis-suppressive effector induced in the context of BTKi exposure, was markedly reduced in FHD-286 treated tumors, while remaining unchanged in PB-treated tumors. These data further support a distinct mechanism of action for FHD-286 in suppressing the MEF2B-NDUFA4L2 axis in vivo. The new results are presented in Fig. S10.

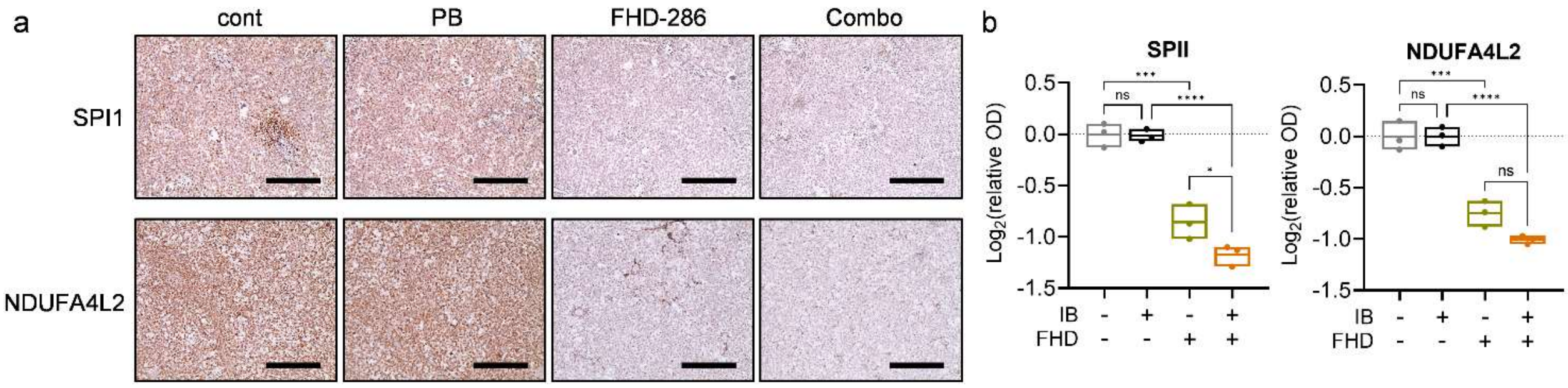

Reviewer Fig. 18. BRG1 inhibition re-sensitizes resistant MCL xenograft to BTKi. (a) Representative IHC images of tumor sections for SPI1, NDUFA4L2, 4-HNE, and cleaved caspase 3 in the indicated treatment groups. Scale bars, 200 μm. (b) Quantification of IHC staining in the tumor sections shown in (a). Data represent mean ± SD.

Minor Comment:

1. Figure 7H. Provide genotype information (BRG1 mutation site in P#2), BRG1 levels, MEF2B/NDUFA4L2/FTH1 expression, and IB resistance status for clarity.

: BRG1 mutation status (including the mutation site in P#2), as well as BRG1, MEF2B, NDUFA4L2, and FTH1 expression levels and IB responsiveness, are provided in Reviewer Fig. 11a, which has now been incorporated into the revised manuscript as Fig. S7m.

Reviewer #3 - Epigenetics (Remarks to the Author):

1. Based on the data presented, one would expect BRG1 mutants are gain-of-function in nature. It would be critical to perform ChIP-seq and/or Cut&Run for wildtype and mutant BRG1 and examine how they may differentially regulate gene expression by cross-referencing with PRO-seq/RNA-seq data.

: As suggested, we performed Cut&Run for GFP-tagged BRG1^{WT} and BRG1^{T910M} and integrated these data with matched RNA-seq from the same model. BRG1^{T910M} exhibited largely overlapping chromatin occupancy with BRG1^{WT}, with a limited subset of ~1,500 differentially occupied sites (Reviewer Fig. 19a, b), arguing against a global gain-of-function mechanism. Instead, assigning these differential peaks to nearby genes and cross-referencing with RNA-seq identified a subset of concordant transcriptional changes (Reviewer Fig. 19c, d), supporting a direct but selective relationship between altered BRG1 occupancy and gene expression.

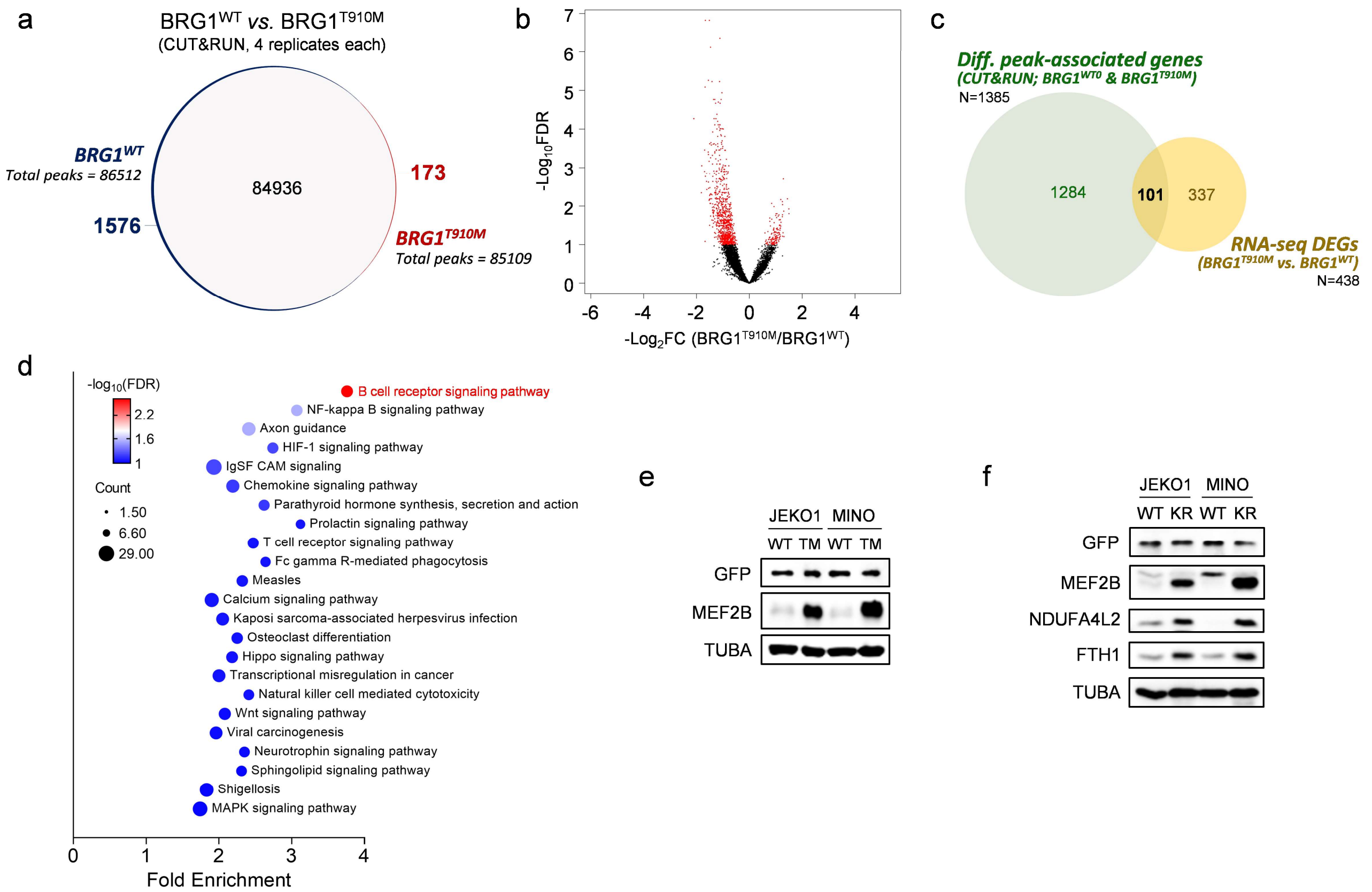

Reviewer Fig. 19. Transcriptional deregulation in BRG1^{mut}-expressing MCL cells. (a) Venn diagram illustrating the overlap of BRG1 binding sites. Anti-GFP Cut&Run sequencing was performed on four biological replicates for venus-tagged BRG1^{WT} and BRG1^{T910M} expressing MINO cells. The Venn diagram displays the high degree of conservation in chromatin occupancy between the two groups with a total of 84,936 common peaks. (b) Volcano plot displaying the peak-normalized differential binding analysis between BRG1^{T910M} and BRG1^{WT} (DESeq2). The x-axis represents Log2 fold change (FC) of BRG1 occupancy, and the y-axis is the significance. The asymmetry of the plot suggests a preferential loss of BRG1 binding intensity in the BRG1^{T910M} relative to BRG1^{WT}. (c) Venn diagram illustrating the overlap between genes associated with differential BRG1 binding sites (Cut&Run; green) and DEGs (RNA-seq; yellow) in BRG1^{T910M} and BRG1^{WT} MINO cells. (d) KEGG analysis of genes associated with differential binding between BRG1^{T910M} and BRG1^{WT}. (e) TF motif enrichment in differential peaks from Cut&Run data comparing MINO BRG1^{T910M} vs. BRG1^{WT}. The 472 known TF binding motifs are ranked by significance (-Log$_{10}$(FDR)).

Motif enrichment analysis of differential sites revealed strong enrichment for ETS family transcription factors (e.g., PU.1/SPI1, SPIB, ETV, ELF) and IRF motifs (Reviewer Fig. 19e), consistent with prior

studies[6,7]. Together, these data support a model in which BRG1^{T910M} drives BTKi resistance not through widespread chromatin reprogramming, but via selective redistribution of BRG1 at specific regulatory elements, resulting in transcriptional reprogramming.

2. In Figure 1G, the authors show that JEKO1 resistant cells have enrichment of ferroptosis suppressor gene sets by GSEA and are unresponsive to BTKi treatment. Do BRG1 T910M cells share the GSEA signature of the resistant cells as the mutant cells also do not respond to BTKi treatment?

: It is an important point. Our GSEA comparing BRG1^{T910M} and BRG1^{WT} cells revealed that BRG1^{T910M} cells exhibit positive enrichment of a ferroptosis suppressor gene set (NES=1.39 q-val=0.049) (Reviewer Fig. 20), consistent with the enrichment observed in BTKi-resistant JEKO1^{R} cells and patient-derived samples (Fig. 1h). In line with this shared gene signature, BRG1^{T910M} cells display resistance to zanubrutinib, ibrutinib, and pirtobrutinib (Fig. 2b).

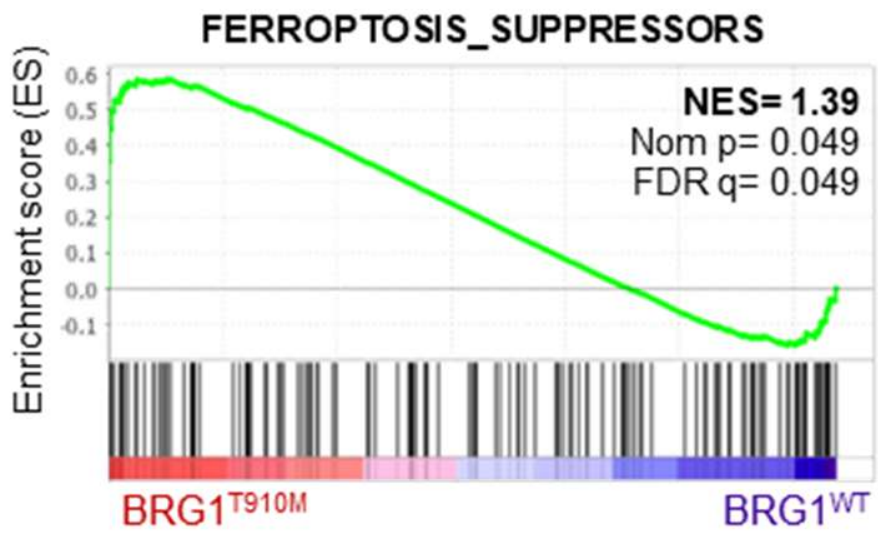

Reviewer Fig. 20. BRG1^{T910M} cells exhibit a suppressed gene signature of ferroptosis drivers. GSEA plots for curated Ferroptosis_Suppressors geneset comparing BRG1^{T910M} vs. BRG1^{WT} MINO cells.

3. The authors cite literature that BRG1 is one of the most frequently genes in MCL and is implicated in BTKi resistance and say that is the premise for testing its potential contribution to BTKi. It would be great, if they could find MCL patient data and check for BRG1 910 mutation and determine if the patient's carrying mutation respond to BTK1i from publicly available database.

: We appreciate the reviewer's point regarding the clinical relevance of our model. Our selection of a BRG1 missense mutation is directly informed by recent clinical evidence (Fig. 2A-B)[8], which identified recurrent BRG1^{T910M} mutations in ibrutinib-resistant MCL patients (Pt5 and Pt28). Notably, our own primary MCL cohort independently identified a BRG1^{M781I} mutation within the same SNF2 ATPase domain in a resistant patient sample (Fig. 8a, S9g). These consistent observations across cohorts provide a strong biological rationale for using this specific functional model to investigate how aberrant BRG1 activity contributes to ferroptosis resistance in the context of acquired BTKi resistance.

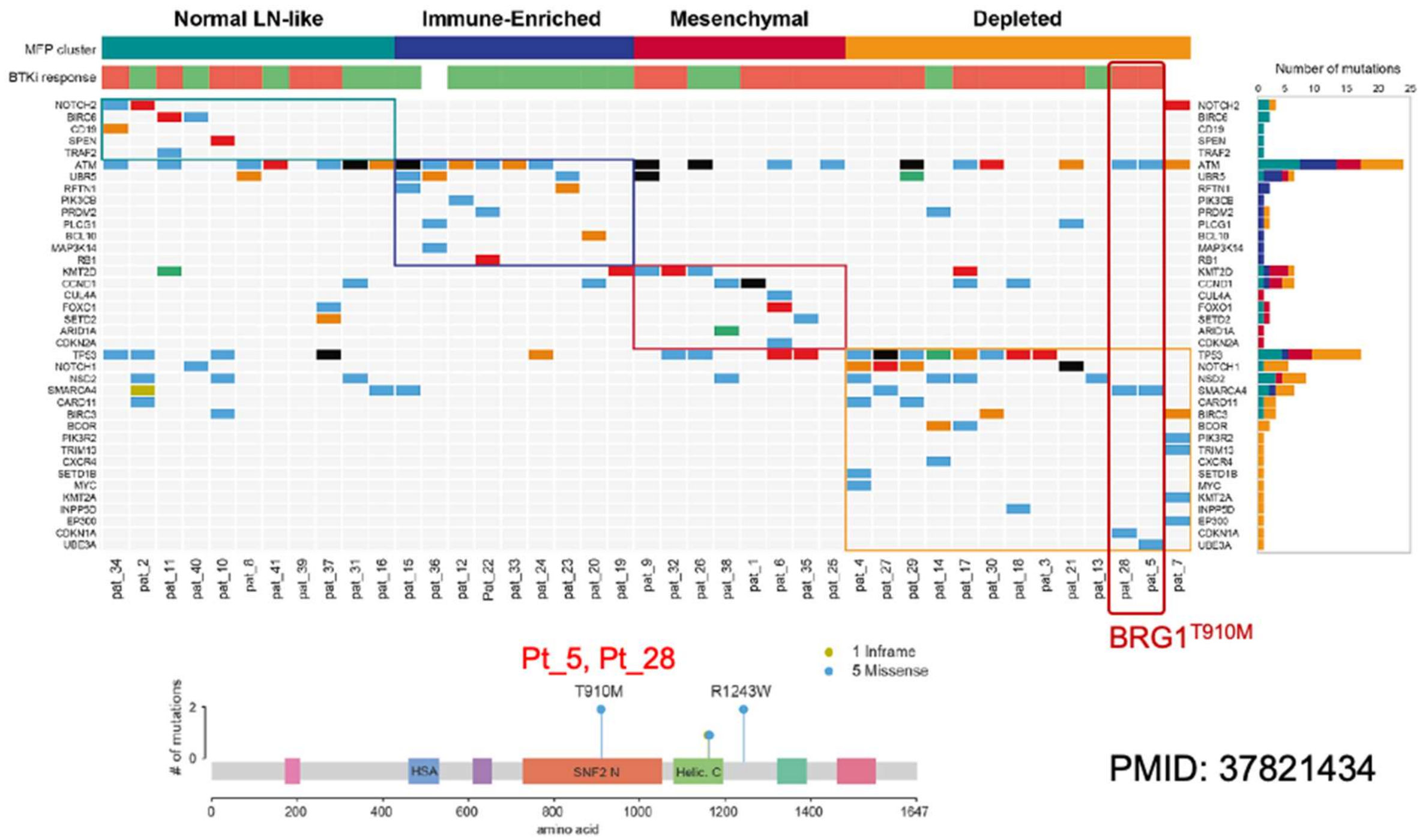

Reviewer Fig. 22. MCL patients harboring BRG1^{T910M} exhibit resistance to BTKi. Oncoplot showing the distribution of somatic mutations, with BRG1^{T910M} among the recurrent mutation in MCL. Data adapted from [PMID:37821434].

4. In figure 5C, the authors did Cut & Run to show that MEF2B directly regulates NDUFA4L2 and FTH1. It would be nice if the authors added MEF2B ChIP-qPCR validation. Likewise, the identification of MEF2B is central to the proposed pathway. However, the evidence that BRG1 may regulate MEF2B transcription is only correlative (expression and chromatin accessibility of MEF2B between BRG1 mutants and wildtype). Please perform ChIP-qPCR to establish a direct mechanistic link.

: As suggested, we performed Cut&Run-qPCR using independently prepared DNA samples and corresponding libraries. Primers were designed to span the MEF2B-bound regions identified at the NDUFA4L2 and FTH1 loci, with negative gene desert regions included as controls (Reviewer Fig. 23). Data has been incorporated into the revised manuscript as Fig. S5a.

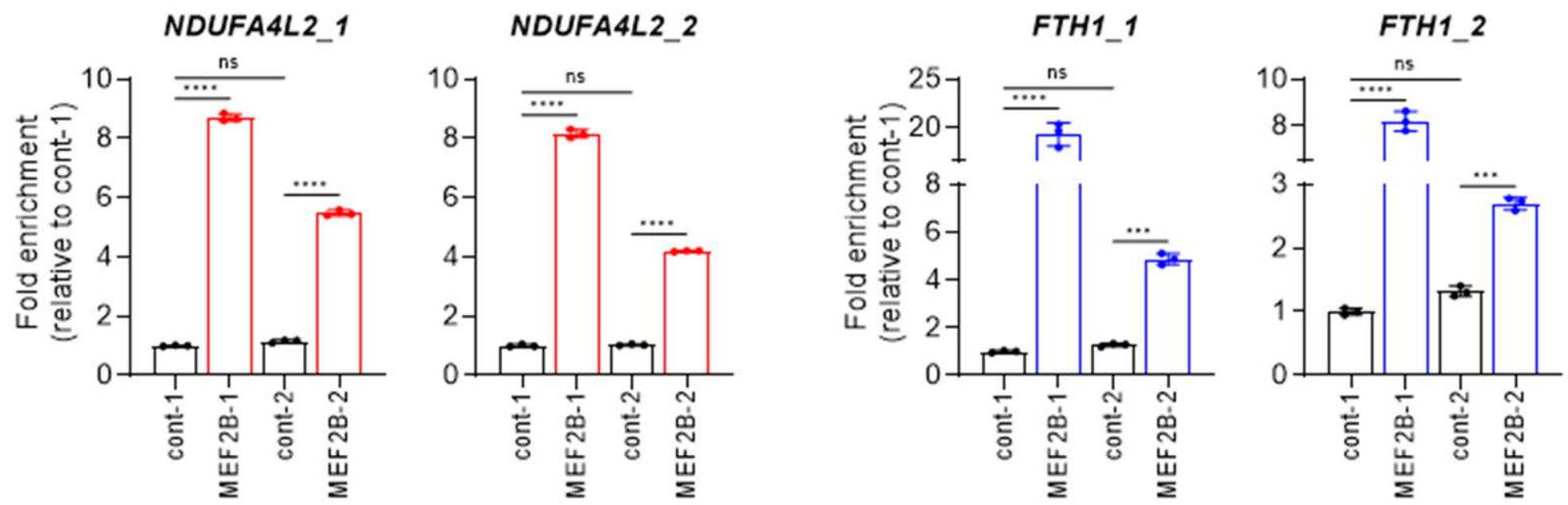

Reviewer Fig. 23. NDUFA4L2 serves as the key downstream effector of MEF2B to mediate BTKi-induced ferroptosis resistance. ChIP-qPCR analysis of control and MEF2B^{WT}-expressing JEKO1 cells at the NDUFA4L2 and FTH1 loci.

Regarding regulation of MEF2B by BRG1 mutant, our data support an indirect mechanism. Please see our response to Reviewer #1, point 2, for details.

5. In figure 7C, the authors show that BRG1 inhibitor in combination with BTKi sensitizes resistant cells to BTKi. The authors can strengthen this by doing IHC/IF of ferroptosis markers in tumor samples. In addition to ferroptosis markers, they can also include caspase- 3 as a negative control to show that indeed the combination induces ferroptosis and not apoptosis in vivo.

: As suggested, we show immunohistochemical analysis of the lipid peroxidation product 4-hydroxynonenal (4-HNE) as a proxy for ferroptosis response. Combined FHD-286 and pirtobrutinib treatment produced the highest 4-HNE levels, consistent with therapeutic synergy, and significantly increased cleaved caspase-3 (Reviewer Fig. 24).

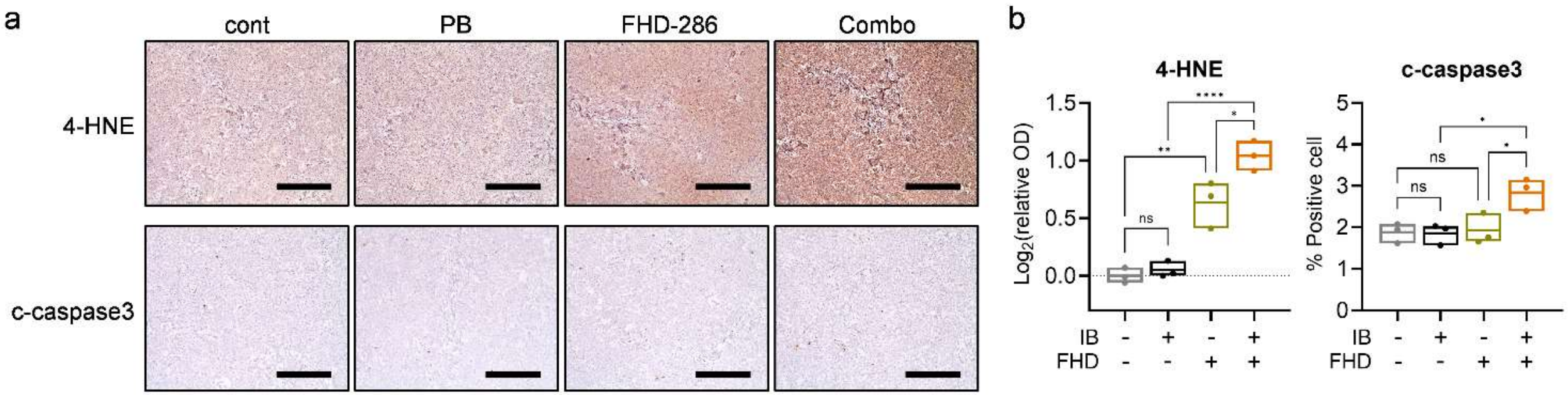

Reviewer Fig. 24. BRG1 inhibition re-sensitizes resistant MCL xenograft to BTKi. (a) Representative IHC images of tumor sections for 4-HNE and cleaved caspase 3 in the indicated treatment groups. Scale bars, 200 μm. (b) Quantification of IHC staining in the tumor sections shown in (a). Data represent mean ± SD.

We interpret increased apoptosis in the combined treatment group in two ways. First, our data showed that FHD-286-induced cell death is not exclusively ferroptosis (Fig. 2I). Additionally, we suspect there is a ferroptosis-driven bystander effect in vivo. Ferroptotic tumor cells release diffusible DAMPs (e.g., HMGB1), ROS, and oxidized lipids that propagate oxidative stress to neighboring cells[9], which may activate intrinsic, caspase-dependent apoptosis. Data is now added as Fig. S10.

6. The study links BRG1 mutations to ferroptosis regulation and BTKi sensitivity, suggesting a role in primary resistance. However, most data are derived from induced resistant (BTKi-R) cell lines, which model secondary/acquired resistance. Please clarify whether the goal is to explain baseline BTKi sensitivity differences or to understand how BRG1 contributes to acquired resistance. And it would be valuable to show how BRG1 expression or activity changes during the development of resistance or between BTKi-sensitive and -resistant lines.

: We thank the reviewer for this important conceptual point. Our goal is to define how dysregulated BRG1 activity contributes to acquired BTKi resistance, rather than to model primary mutation-driven resistance. Accordingly, we employed induced BTKi-R models to interrogate adaptive chromatin and transcriptional changes that arise under sustained BTKi pressure.

In this context, the cancer-associated $BRG1^{T910M}$ or $BRG1^{K785R}$ mutants are used as a functional model of deregulated BRG1 activity, not as a surrogate for primary resistance. It enables us to model aberrant BRG1-dependent chromatin remodeling that reinforces ferroptosis suppression during the acquisition and maintenance of resistance.

Consistent with this framework, BRG1 expression and/or activity is increased in BTKi-R cells relative to isogenic sensitive counterparts, and BRG1 transcript levels are elevated in patient samples at progression versus complete response. Together, these data support a model in which adaptive upregulation or deregulation of BRG1 promotes ferroptosis resistance under chronic BTKi exposure.

7. The manuscript reports "134 overlapping DEGs from PRO-seq (|FC| > 1) of JEKO1 and RNA-seq (|FC| > 1.5) of MINO cells." Combining DEG lists derived from different cell lines and platforms (PRO-seq vs

RNA-seq) without controlling for cell-line- or assay-specific effects is not statistically justified. This method may introduce significant confounding variables and potential bias. Please provide a rationale for this intersection approach. Besides, what are the pathway analysis results if the two datasets are analyzed separately? Will the two datasets indicate consistent pathway findings?

: We thank the reviewer for this important concern. In the revised manuscript, we no longer intersect DEG lists derived from different cell lines and fold-change thresholds. Instead, differential expression for each assay is now computed within the same cell line using biological replicates, and statistical significance is defined using DESeq2 with FDR<0.1. Gene lists derived under this consistent criterion were then compared across platforms.

For pathway analysis, we first analyzed each dataset independently using genes meeting FDR<0.1. Notably, both RNA-seq and PRO-seq datasets independently identified B cell receptor signaling as one of the significantly enriched pathways (Reviewer Fig. 25). We then examined the overlap between assays (Fig. 3a) and found that the commonly regulated genes showed B cell receptor signaling as the top-ranked pathway (Fig. 3b).

PRO-seq (913 genes)

Rank	Term	Count	Fold Enrichment	-log(FDR)
1	Viral protein interaction with cytokine and cytokine receptor	17	4.45	3.58
2	B cell receptor signaling pathway	15	4.31	3.32
3	Natural killer cell mediated cytotoxicity	17	3.80	3.32
4	Cytokine-cytokine receptor interaction	29	2.55	3.32
5	Th1 and Th2 cell differentiation	15	4.22	3.32
6	Pathways in cancer	42	2.06	3.32
7	Malaria	11	5.76	3.32
8	Toll-like receptor signaling pathway	16	3.84	3.32
9	IgSF CAM signaling	27	2.34	2.62
10	Cell adhesion molecules	18	2.94	2.50

RNA-seq (1,432 genes)

Rank	Term	Count	Fold Enrichment	-log(FDR)
1	Epstein-Barr virus infection	47	3.23	9.52
2	Human cytomegalovirus infection	44	2.73	6.58
3	Herpes simplex virus 1 infection	38	2.93	6.41
4	NOD-like receptor signaling pathway	38	2.84	6.14
5	Human T-cell leukemia virus 1 infection	41	2.58	5.65
6	Type I diabetes mellitus	16	5.13	5.12
7	Viral myocarditis	20	4.03	5.07
8	B cell receptor signaling pathway	23	3.57	5.07
9	Allograft rejection	14	5.07	4.34
10	Cell adhesion molecules	30	2.65	4.21

Reviewer Fig. 25. B cell receptor signaling is independently enriched in both RNA-seq and PRO-seq datasets. KEGG pathway enrichment analysis of DEGs identified by PRO-seq and RNA-seq (FHD-286 vs control; FDR<0.1, |FC|>1.5) in MINO cells. The top 10 enriched pathways from each analysis are shown.

Together, these analyses confirm that pathway-level conclusions are robust across platforms and not attributable to cell line or assay-specific effects.

8. According to the GEO dataset (GSE305144), each RNA-seq group includes only one sample. Were any biological or technical replicates performed? If standard methods such as DESeq2/edgeR/limma were used, please describe how variance was estimated. Without replicates and proper statistical tests, the validity of all downstream conclusions that rely on these transcriptomic datasets would be fundamentally compromised. The authors must transparently describe the method used to call DEGs and provide statistical significance measures (e.g., p-values, FDR). If no replicates were performed, this must be explicitly stated and all key findings from these datasets must be independently validated with other methods (e.g., qPCR, WB).

: We generated additional RNA-seq biological replicates and re-derived DEGs, which models gene counts with a Negative Binomial distribution. Variance was estimated using Empirical Bayes shrinkage to regularize gene-wise dispersion toward the global trend. Significant features were identified using a Wald test with a Benjamini-Hochberg adjusted P-value (padj, FDR)<0.1. Detailed data analysis description is provided under Supplementary Methods section. The results were consistent with our initial findings. And also key findings were validated with WB throughout the manuscript.

9. Different thresholds (0.6, 1.0, and 1.5) are used in different analyses. Please justify these choices and demonstrate whether results are robust under consistent criteria or based on FDR-adjusted significance.

: We thank the reviewer for this comment. All analyses were updated using FDR-adjusted significance thresholds. The key findings remain unchanged under this criterion, and all major conclusions are preserved. Where applicable, to narrow the gene set beyond statistical significance alone, we additionally applied a uniform fold-change cutoff of 1.5 to enrich for more biologically meaningful changes. These updates have been incorporated into the revised figures and corresponding legends.

Minor comments

1. To strengthen translational relevance, the authors could explore whether BRG1 mutation or BRG1/MEF2B/NDUFA4C2 axis expression correlates with BTKi response in patient datasets, if available.

: We appreciate this insightful suggestion. In response, we interrogated publicly available single-cell transcriptomic datasets from MCL patient samples to assess the clinical relevance of BRG1-driven transcriptional programs in BTKi response. Specifically, we analyzed samples from patients achieving complete response (CR, 6 specimens) or exhibiting progressive disease (PD, 4 specimens) following BTKi therapy. Notably, BRG1 expression was significantly elevated in PD compared to CR samples (Reviewer Fig. 16), supporting its association with BTKi resistance. Furthermore, fraction of cells co-expressing BRG1 with MEF2B, NDUFA4L2, and FTH1 was higher in PD, consistent with activation of a coordinated transcriptional program. These findings align with our mechanistic data and support a model in which BRG1-MEF2B-NDUFA4L2 signaling promotes a ferroptosis-resistant state, in part through modulation of mitochondrial function.

To further strengthen translational relevance, we additionally assessed protein expression of key effectors in primary BTKi-sensitive versus -resistant MCL samples, confirming concordant upregulation in resistant cases (Reviewer Fig. 11a). Collectively, these data provide clinical support for targeting BRG1-driven programs as a strategy to overcome BTKi resistance. Related data are all included in our revised manuscript as Fig. 7, Fig. S7m.

2. Since ferroptosis is regulated by multiple pathways, please discuss how dominant this axis is compared with known regulators (e.g., GPX4, SLC7A11).

: We appreciate this important point. Our RNA-seq and PRO-seq analyses show that FHD-286 does not transcriptionally suppress canonical ferroptosis regulators, including GPX4 and SLC7A11 with high baseline expression (Fig. 3a), indicating that this axis operates independently of these pathways. Despite intact expression of these core regulators, cells remain susceptible to FHD-induced ferroptosis upon disruption of the MEF2B-NDUFA4L2 axis.

Mechanistically, this axis appears to act upstream of canonical lipid peroxide detoxification by limiting mitochondrial ROS production and labile iron accumulation under conditions of mitochondrial stress. As such, it does not represent a universally dominant ferroptosis pathway but rather defines a context-specific regulator that modulates the cellular threshold for ferroptotic cell death, thereby constraining the protective capacity of GPX4-mediated lipid peroxide scavenging.

3. Previous studies (Cell Reports, 2024) have shown that BRG1-deficient cancers are hypersensitive to ferroptosis induction. Please clarify whether BRG1 inhibition alone would be therapeutically sufficient, or whether ferroptosis inducers should be combined for greater efficacy.

: In our updated manuscript, we present both FHD-286 treated and sgBRG1-based genetic depletion inducing lipid peroxidation (Fig. S2j), showing that BRG1 inhibition alone is sufficient to increase ferroptosis. Our results suggest that BRG1 inhibition can be active as a single agent but that combination therapy is more effective in our BTKi-resistant MCL setting (Fig. 8). In principle, combining BRG1 inhibition with a direct ferroptosis inducer (e.g., targeting GPX4) could further enhance efficacy, consistent with the broader literature showing that SWI/SNF perturbation can increase ferroptosis sensitivity. However, in this study we focused on the clinically relevant and mechanistically aligned combination of FHD-286 with BTKi, which produced clear synergistic in vivo effects.

4. FHD-286 is a BRG1/BRM inhibitor. Please discuss how to rule out the effect of BRM in this study.

: We thank the reviewer for raising this important point. To address the potential contribution of BRM inhibition, we performed sgBRM-mediated genetic dropout experiments and incorporated these data into the revised manuscript (Fig. S2h-i). Notably, BRM depletion alone had minimal impact on MCL cell viability, in contrast to the pronounced growth suppression observed upon BRG1 loss. These findings indicate that BRM is not independently required for MCL cell survival. Consistent with this, our CRISPR/Cas9 screen of epigenetic regulator dependencies identified BRG1, but not BRM, as a critical vulnerability in MCL (Fig. S2f). Together, these orthogonal genetic approaches support a model in which the dominant biological effects of FHD-286 in this context are primarily mediated through BRG1 inhibition, with minimal contribution from BRM.

5. The overall methodology description for sequencing and bioinformatic analysis is insufficient.

: We appreciate this comment and have expanded the Supplementary Methods section to provide a comprehensive description of the sequencing and bioinformatic analyses, including preprocessing, alignment, quality control, normalization, and statistical procedures. These revisions enhance the clarity and reproducibility of the study.

6. In Fig 3C, the peak for CD79B is not convincing. IgG is blank in almost all of their figures, which doesn't appear to make sense.

: We agree with the reviewer and have replaced the track with a more representative example (CD79A) that clearly demonstrates peak enrichment. The previously blank appearance of the IgG control tracks was due to high signal-to-noise and group-linked y-axis scaling. We have now adjusted the scaling across all tracks to ensure that IgG background signal is visible and comparable across panels.

References

1 Lee, H. *et al.* Energy-stress-mediated AMPK activation inhibits ferroptosis. *Nature cell biology* **22**, 225-234, doi:10.1038/s41556-020-0461-8 (2020).

2 West, A. G., Shore, P. & Sharrocks, A. D. DNA binding by MADS-box transcription factors: a molecular mechanism for differential DNA bending. *Molecular and cellular biology* **17**, 2876-2887, doi:10.1128/mcb.17.5.2876 (1997).

3 Di Liberto, M. *et al.* Disrupting IL10 Signaling Overcomes Compound Copy Number Variation-Driven Clinical Resistance to CDK4/6 and BTK Inhibition in Mantle Cell Lymphoma. *Blood* **144**, 831-831, doi:10.1182/blood-2024-209415 %J Blood (2024).

4 Martin, P. *et al.* A phase 1 trial of ibrutinib plus palbociclib in previously treated mantle cell lymphoma. *Blood* **133**, 1201-1204, doi:10.1182/blood-2018-11-886457 (2019).

5 Garrett-Sinha, L. A. *et al.* PU.1 and Spi-B are required for normal B cell receptor-mediated signal transduction. *Immunity* **10**, 399-408, doi:10.1016/s1074-7613(00)80040-0 (1999).

6 Deng, Q. *et al.* SMARCA4 is a haploinsufficient B cell lymphoma tumor suppressor that fine-tunes centrocyte cell fate decisions. *Cancer cell* **42**, 605-622.e611, doi:10.1016/j.ccell.2024.02.011 (2024).

7 Barisic, D. *et al.* ARID1A orchestrates SWI/SNF-mediated sequential binding of transcription factors with ARID1A loss driving pre-memory B cell fate and lymphomagenesis. *Cancer cell* **42**, 583-604.e511, doi:10.1016/j.ccell.2024.02.010 (2024).

8 Jain, P. *et al.* Immune-depleted tumor microenvironment is associated with poor outcomes and BTK inhibitor resistance in mantle cell lymphoma. *Blood cancer journal* **13**, 156, doi:10.1038/s41408-023-00927-2 (2023).

9 Roeck, B. F. *et al.* Ferroptosis spreads to neighboring cells via plasma membrane contacts. *Nat Commun* **16**, 2951, doi:10.1038/s41467-025-58175-w (2025).

NCOMMS-25-77445A

Point by point response

We thank the reviewers for the careful and constructive re-evaluation of our manuscript.

Reviewer #2 (Remarks to the Author):

Minor comments:

1. SOX11 has been reported to limit lethal ROS production in MCL by inducing oxidative stress modulation (PMID: 38570586). This effect is mediated through the SOX11:BRG1 complex, which regulates PRDX2 expression (PMID: 40739087), thereby inhibiting ferroptosis-mediated cell death. Considering these findings, additional analyses are required to demonstrate that resistance to BTK inhibitors (ibrutinib, zanubrutinib, pirtobrutinib) mediated by ferroptosis suppression is independent of the SOX11:BRG1 complex.

Moreover, as direct regulation of MEF2B by BRG1 has not been demonstrated in MCL, the absence of changes in Fe^{2+} levels, ROS, lipid peroxidation, ATF4, MEF2B, and related markers upon BRG1 depletion or BRG1T910M ectopic expression in a SOX11-negative MCL cell line would further support a SOX11-independent, BRG1-mediated mechanism of BTKi resistance. Such data would strengthen the proposed ATF4–MEF2B–NDUFA4L2/FTH1 axis as a direct inhibitor of ferroptosis-driven cell death.

Although we recognize the emerging role of the SOX11:BRG1 axis in oxidative stress, our data indicate that the aberrant BRG1-MEF2B-NDUFA4L2/FTH1 axis confers ferroptosis resistance through a mechanism distinct from and independent of SOX11 expression in our experimental system. To directly address this reviewer's point, we performed a new experiment comparing isogenic SOX11-proficient and SOX11-depleted MINO MCL cells expressing either $BRG1^{WT}$ or $BRG1^{T910M}$ for their ferroptosis sensitivity (i.e., lipid peroxidation and cell death) in response to all three BTK inhibitors. Our results show that SOX11 depletion did not induce notable changes in the ferroptosis response (Reviewer Fig. 1a, b), nor did it alter the expression levels of MEF2B, NDUFA4L2, or FTH1 (Reviewer Fig. 1c).

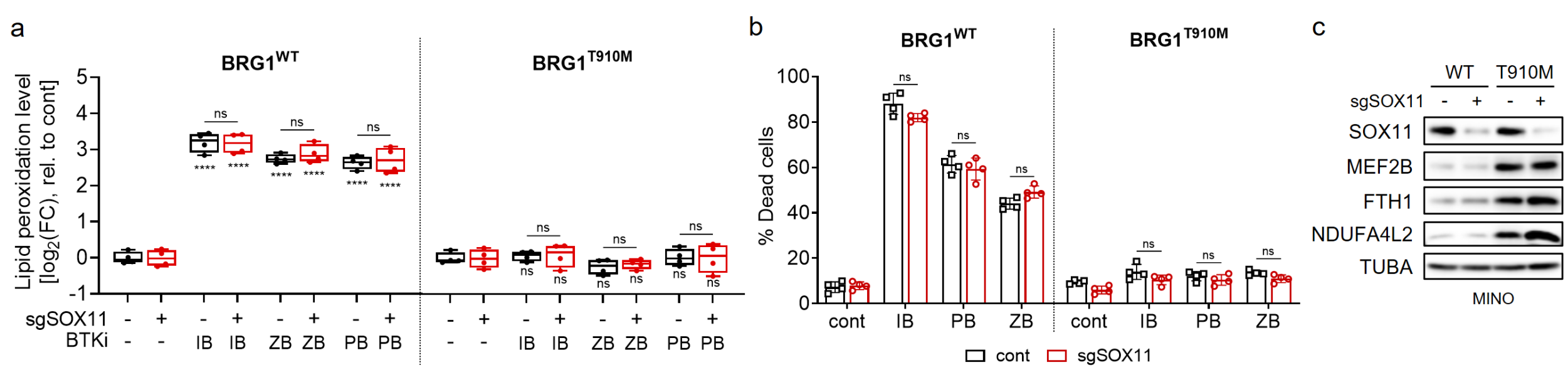

Reviewer Fig. 1. MEF2B-NDUFA4L2/FTH1 Axis suppresses ferroptosis in a SOX11-independent manner. (a-b) Changes in lipid peroxidation (a; 72 h) and cell death (b; 96 h) upon treatment of BTKi inhibitors (5 μM) in SOX11-positive and -depleted $MINO^{WT}$ and $MINO^{T910M}$ cells. N=4, mean ± SD. Statistical significance was determined by one-way ANOVA with Tukey's test; ****$p<0.0001$. (c) Immunoblot of SOX11-depleted $MINO^{WT}$ and $MINO^{T910M}$ cells.

Moreover, our SOX11 protein expression data as well as transcriptomic data comparing $BRG1^{WT}$ and $BRG1^{T910M}$ cells indicate that SOX11 levels remain unchanged, suggesting that the observed resistance pathway is likely SOX11-independent.

2. It should be evaluated whether pharmacological inhibition of BRG1 using alternative BRG1 inhibitors produces effects comparable to those observed with FHD-286 in MCL cell models. This would help establish whether the observed phenotypes are specifically attributable to BRG1 inhibition rather than compound-specific effects.

We agree that independent pharmacologic validation can strengthen target specificity. However, currently available BRG1 inhibitors, including FHD-286, are dual BRG1/BRM ATPase inhibitors, and no selective BRG1-specific compounds are available.

To address target specificity, we therefore employed orthogonal genetic approaches. BRG1 depletion phenocopied the effects of FHD-286, including suppression of the MEF2B-NDUFA4L2/FTH1 axis and induction of lipid peroxidation (Fig. 7e, Supplementary Fig. 2j). In contrast, BRM depletion had minimal impact on cell viability and did not recapitulate these effects (Supplementary Fig. 2h, 2i). Together, these data support that the observed phenotypes reflect on-target inhibition of BRG1-dependent transcriptional programs rather than compound-specific effects, despite the dual activity of currently available inhibitors.

3. Given that BRG1 is ubiquitously expressed and essential for cell viability, that the BRG1T910M mutation is rare in MCL, and that FHD-286–mediated inhibition is independent of BRG1 expression levels, the authors should discuss how representative and clinically relevant the proposed BRG1–MEF2B–NDUFA4L2/FTH1 axis is in contributing to ferroptosis-mediated BTKi resistance in MCL.

We thank the reviewer for this important point. We agree that BRG1 is ubiquitously expressed and contextually essential, that $BRG1^{T910M}$ is rare in MCL, and that FHD-286 sensitivity does not correlate with BRG1 expression levels. Accordingly, we clarify that we do not propose this axis as mutation-driven or universal. Rather, our data supports a model in which functional dysregulation of a BRG1-dependent transcriptional program contributes to ferroptosis suppression in BTKi-resistant MCL.

Importantly, this pathway is not restricted to engineered BRG1 mutants. BTKi-resistant cell lines and primary progressive disease samples show a trend of coordinated upregulation of MEF2B, NDUFA4L2. In addition, both genetic BRG1 depletion and pharmacologic inhibition suppress this axis, indicating pathway-level dependency independent of BRG1 mutation or expression level.

We therefore suggest that the activity of the BRG1-MEF2B-NDUFA4L2 axis, rather than BRG1 genotype or abundance alone, defines a clinically relevant ferroptosis-resistant state in a subset of BTKi-refractory MCL.

4. The authors should discuss why they have chosen this specific mutation and if other missense mutations affecting the SNF2 or other domains that may affect its catalititc activity may have the same functional effect as the BRG1T910M in MCL.

Our selection of a BRG1 missense mutation is directly informed by recent clinical evidence[1] (Reviewer Fig. 2), which identified recurrent $BRG1^{T910M}$ mutations in ibrutinib-resistant MCL patients (Pt5 and Pt28). Notably, our own primary MCL cohort independently identified a $BRG1^{M781I}$ mutation within the SNF2 ATPase domain in a resistant patient sample (Fig. 8a, Supplementary Fig. 9g). Importantly, similar ferroptosis-resistant phenotypes were observed with an independent MCL-associated mutant (K785R, Fig. 2a, Supplementary Fig. 2d), indicating that this effect is not specific to T910M. These consistent observations provide a strong biological rationale for using this specific functional model to investigate how aberrant BRG1 activity contributes to ferroptosis resistance in the context of acquired BTKi resistance.

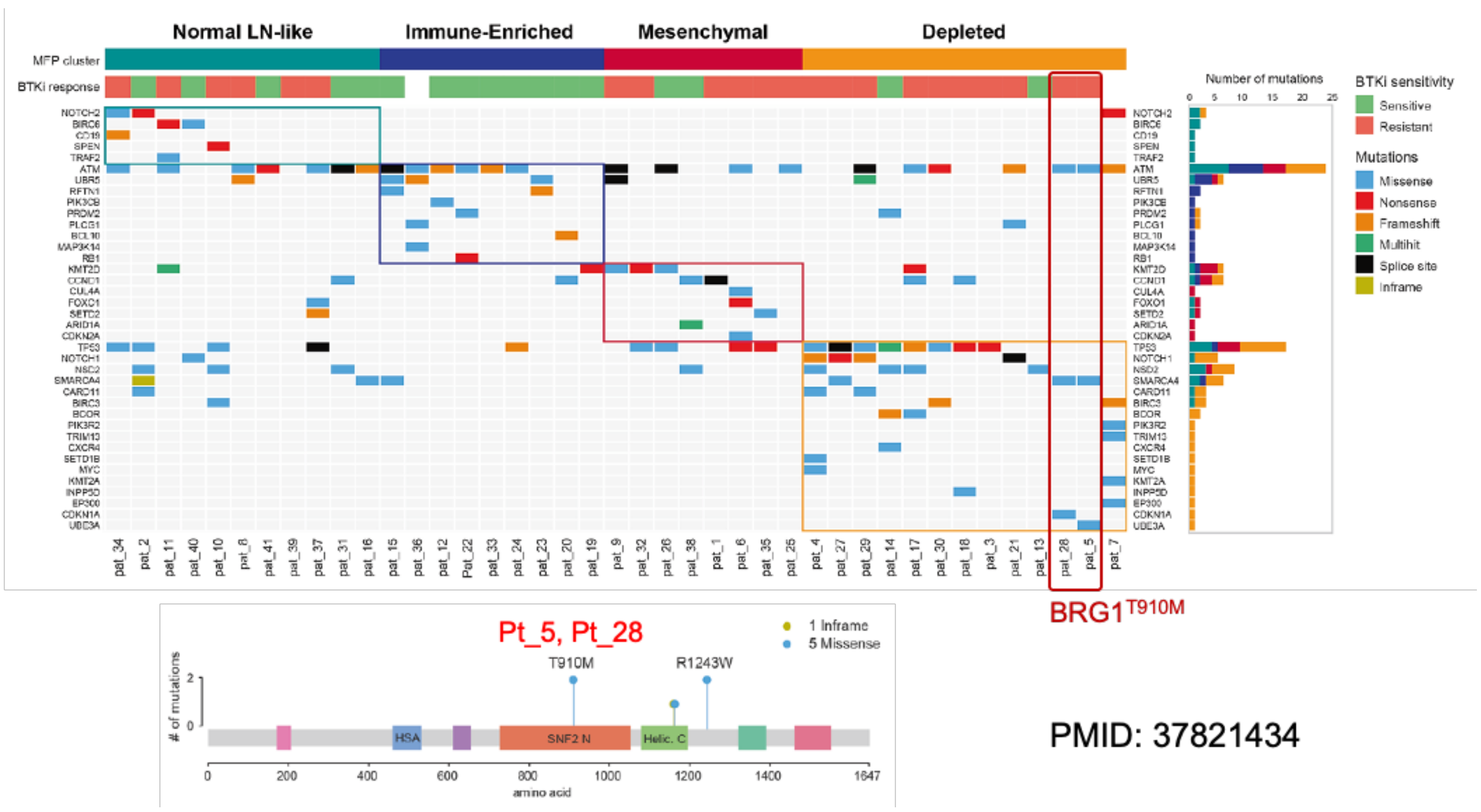

Reviewer Fig. 2. MCL patients harboring $BRG1^{T910M}$ exhibit resistance to BTKi. Oncoplot showing the distribution of somatic mutations, with $BRG1^{T910M}$ among the recurrent mutation in MCL. Data adapted from [PMID:37821434].

5. The Supplementary Tables contain multiple errors and inconsistencies and must be revised to provide accurate and experiment-specific information.

o Supplementary Table S2 is inadequately described, and essential methodological and experimental details are missing.

o The same table (Supplementary Table S3) has been incorrectly provided for the PRO-seq, RNA-seq, and CUT&RUN experiments. A similar issue affects the MEF2B DEG and MEF2B CUT&RUN datasets.

o Distinct and appropriately labeled tables should be supplied for each dataset as follows:
♣ Supplementary Table S3: BRG1 WT/MUT PRO-seq
♣ Supplementary Table S4: BRG1 WT/MUT RNA-seq
♣ Supplementary Table S5: BRG1 WT/MUT CUT&RUN-seq
♣ Supplementary Table S6: MEF2B DEG RNA-seq
♣ Supplementary Table S7: MEF2B CUT&RUN-seq

o Please ensure that each table is complete, accurately labeled, and corresponds unambiguously to the associated experiment.

As suggested by the reviewer, we have separated the original table into eight Supplementary tables (Supplementary table 3-10) and included them in the revised submission. For Supplementary table 2, we now cite our previously published study describing the sgRNA library generation and CRISPR screening [2, 3].

References

1. Jain P*, et al.* Immune-depleted tumor microenvironment is associated with poor outcomes and BTK inhibitor resistance in mantle cell lymphoma. *Blood Cancer J* **13**, 156 (2023).

2. Li F*, et al.* Histone demethylase KDM2A is a selective vulnerability of cancers relying on alternative telomere maintenance. *Nature communications* **14**, 1756 (2023).

3. Jang JY*, et al.* A FOXO1-dependent transcription network is a targetable vulnerability of mantle cell lymphomas. *J Clin Invest* **132**, (2022).

Reviewer #3 (Remarks to the Author):

The authors have addressed most of the concerns and the extensive revision has significantly elevated the mechanistic depth and translational relevance of this study. I only have a few minor clarifications for the authors to consider.

We are grateful for the reviewer's constructive feedback throughout this process. We have provided the requested clarifications below and have updated the manuscript accordingly.

1. The methodology section currently lacks sufficient detail regarding the single-cell analysis. The authors are recommended to explicitly describe the methods and criteria used to distinguish malignant MCL cells from normal B cells. Furthermore, please include essential methodological details, such as quality control parameters, data integration, and batch-effect correction methods, in the Supplementary Methods.

We have now expanded the Supplementary Methods to provide a comprehensive description of data processing, quality control, batch correction, and the criteria used to distinguish malignant MCL cells from normal B cells.

2. The scRNA-seq analysis identified 4 clusters within the MCL cell population. However, there is currently no functional description of these sub-populations. It would be valuable if the authors could perform differential gene and pathway analysis on these subclusters, and investigate whether the relative proportions differ significantly between CR and PD patients. If a compositional shift exists between the two clinical states, exploring the evolutionary trajectory and differential transcriptional regulation among these clusters would be highly meaningful and could provide deeper insights into how the acquired resistance-associated transcriptional program evolves under BTKi pressure.

We agree that differential gene and pathway analysis across the identified MCL subclusters, as well as assessment of compositional shifts between CR and PD states, would provide important insight into transcriptional evolution under BTKi pressure.

In this study, our use of scRNA-seq is focused on defining reproducible MCL cell states to interpret the significance of BRG1 elevation in the context of therapy response. A comprehensive functional dissection of these subclusters including differential expression, pathway enrichment, and trajectory analyses across clinical states, is being completed as part of a separate scRNA-seq-centered manuscript led by co-authors, which is in advanced stages of preparation.

To maintain clarity and avoid redundancy, we have limited the analysis here to features directly supporting the conclusions of this manuscript and added text to direct readers to the forthcoming study for a detailed characterization of subcluster-specific transcriptional programs and their evolution during treatment. Notably, preliminary analyses from this companion work support compositional shifts across these clusters between clinical response states, consistent with the reviewer's suggestion.

To provide additional context, we now include a brief description of the identified clusters. Cluster 1 exhibits a resting-like transcriptional state resembling quiescent B cells. Cluster 2 reflects an activated state enriched for BCR signaling, cytokine response, and inflammatory pathways.

Cluster 3 represents a non-cycling, persistent population with features of stress adaptation that accumulates with disease progression. Cluster 4 comprises a highly proliferative population with strong cell cycle and biosynthetic signatures, enriched in progressive disease.

3. When comparing the expression levels of the target genes (BRG1, MEF2B, NDUFA4L2, and FTH1) between the PD and CR groups, the statistical analyses should be performed at the sample level. Please provide statistical comparisons between the CR and PD cohorts using sample-level metrics, this can be done either by comparing the average expression level per patient sample or by comparing the proportion of cells expressing the genes of interest across the individual samples.

We appreciate this suggestion and added statistical analyses at the sample levels (Supplementary Fig. 7m).

4. In figure 8a, please state statistical significance.

Thanks for pointing it out. We added p-values for Figure 8a.
