# Supplementary material for: BRG1-mediated suppression of ferroptosis underlies BTK inhibitor resistance
Source: Nat Commun. 2026 Jul 2;17:8220. doi: 10.1038/s41467-026-75123-4 (PMC13462549; doi:10.1038/s41467-026-75123-4)
Supplement: Supplementary file 3 [file 41467_2026_75123_MOESM1_ESM.pdf]

Supplementary Information

BRG1-Mediated Suppression of Ferroptosis Underlies BTK Inhibitor Resistance

Soo-Yeon Hwang[1], Xiangao Huang[1,2], Helgi Nikolli[1,3], Belem Yoval-Sánchez[3], Sieun Yang[4], Jeongbin Heo[5], Peter Martin[6], Caitlin Gribbin[7], Preetesh Jain[8], Michael Wang[9], Lalit Sehgal[10], Robert A. Baiocchi[10], Inah Hwang[4], Maurizio DiLiberto[1,2], Alexander Galkin[3], Hojoong Kwak[5], Lapo Alinari[10], Selina Chen-Kiang[1,2], Hongwu Zheng[1]*, Jihye Paik[1,2]*

[1]*Department of Pathology and Laboratory Medicine, Weil Cornell Medicine, New York, NY*

[2]*Meyer Cancer Center, Weill Cornell Medicine and New York Presbyterian Hospital, New York, NY*

[3]*Feil Family Brain and Mind Research Institute, Weill Cornell Medicine, New York, NY, 10065*

[4]*Graduate School of Pharmaceutical Sciences and College of Pharmacy, Ewha Womans University, Seoul, Republic of Korea; Graduate Program in Innovative Biomaterials Convergence, Ewha Womans University, Seoul, Republic of Korea.*[5]*Department of Cell Biology and Anatomy, New York Medical College, Valhalla, NY.*

[6]*Division of Hematology and Medical Oncology, Perlmutter Cancer Center, NYU Langone, New York, NY.*

[7]*Division of Hematology and Medical Oncology, Weill Cornell Medicine, New York, NY.*

[8]*Mays Cancer Center, University of Texas Health San Antonio, San Antonio, TX*

[9]*Department of Lymphoma and Myeloma from The University of Texas MD Anderson Cancer Center, Houston, TX*

[10]*Division of Hematology, Department of Internal Medicine, College of Medicine, The Ohio State University, Columbus, OH, USA*

*Correspondence:

Jihye Paik, Ph.D
Associate Professor of Pathology and Laboratory Medicine
Email: jep2025@med.cornell.edu

Hongwu Zheng, Ph.D
Assistant Professor of Pathology and Laboratory Medicine
Email: hoz4001@med.cornell.edu

Contents

Supplementary Figures...p. 2-14
Supplementary Table 1...p. 15-17

Supplementary Figures

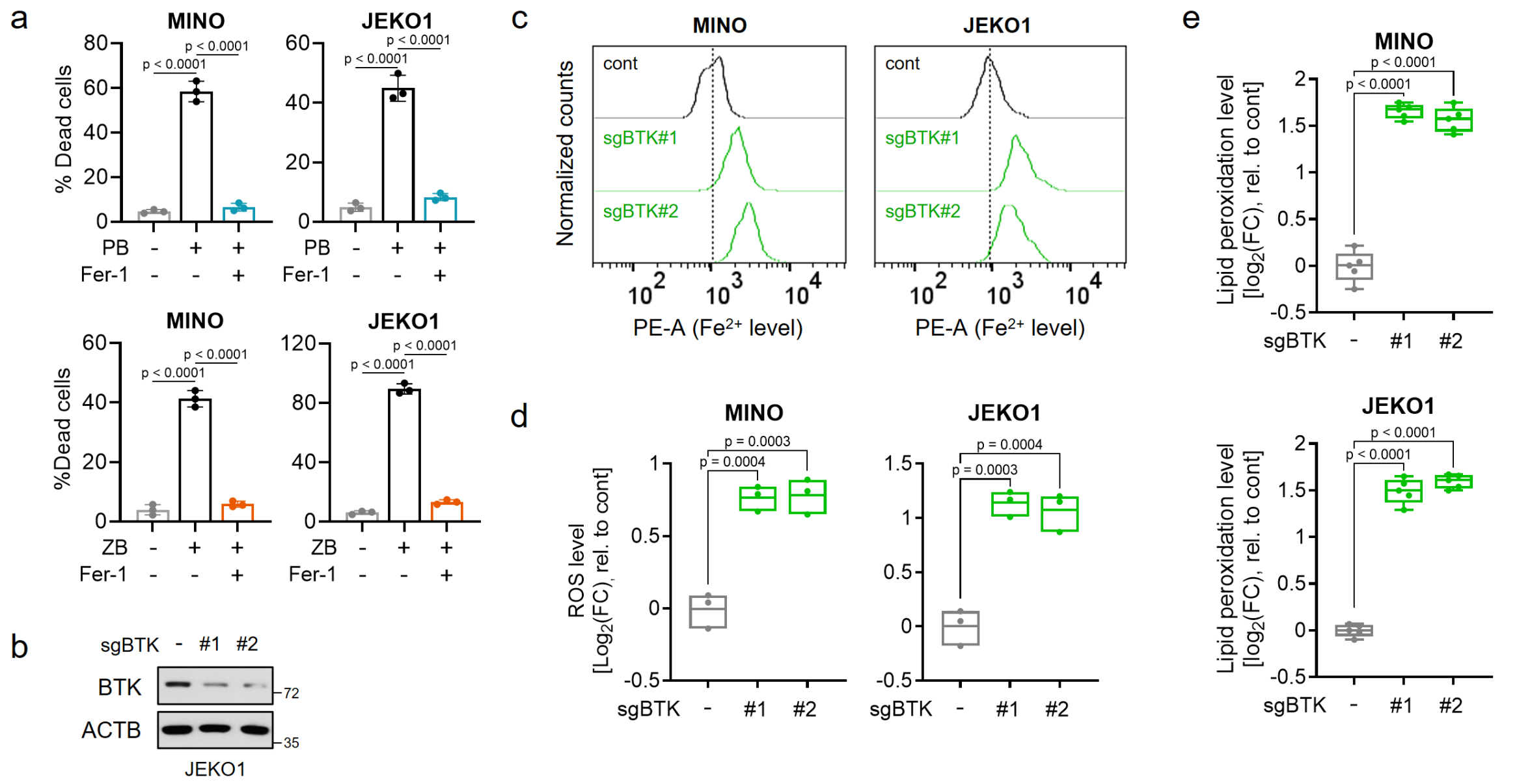

Supplementary Fig. 1. Ferroptosis suppression underlies BTK inhibitor resistance. (a) Effect of Fer-1 (2 µM) on PB or ZB-induced (96 h) cell death in MCL cells. Data represent mean ± s.d. from n = 3 biologically independent experiments. (b) Immunoblot of BTK-depleted JEKO1 cells 5-days post-sgRNA expression, representative of three independent experiments with similar results. (c-e) Changes in labile Fe^{2+} (c), ROS (d) and lipid peroxidation (e) following BTK depletion in MCL cells (6-day post-sgRNA expression). Representative histograms in (c) are from three independent experiments with similar results. Floating bars in (d) show minimum to maximum values with centre lines indicating means; n = 3 biologically independent experiments. Box plots in (e) show medians, 25th-75th percentiles, and minimum to maximum whiskers; n = 5 biologically independent experiments. All BTKi treatments were performed at 5 µM. Statistical significance was determined using one-way ANOVA with Tukey's test.

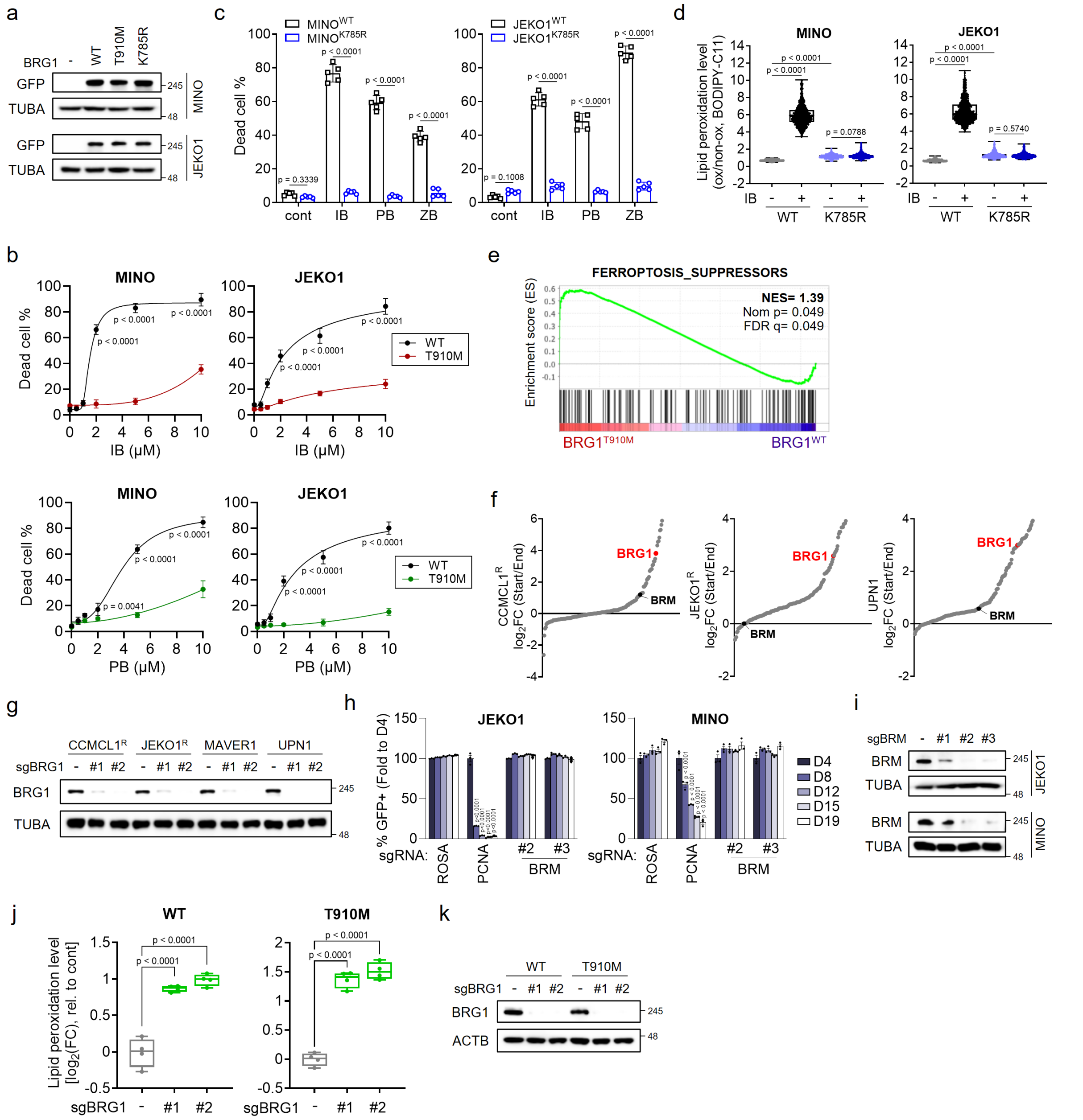

Supplementary Fig. 2. BRG1 regulates ferroptosis response in MCL cells. (a) Immunoblot verification of BRG1^{WT}, BRG1^{T910M}, and BRG1^{K785R} expression in MINO and JEKO1 cells. (b) Dose-response curves of IB and PB in MINO and JEKO1 cells expressing BRG1^{WT} or BRG1^{T910M} (96 h). (c) Cell death response to various BTKi in BRG1^{WT} and BRG1^{K785R} MCL cell lines (96 h). (d) IB-induced changes in lipid peroxidation (72 h) in BRG1^{WT} *vs.* BRG1^{K785R} MCL cells. (e) GSEA plots for curated Ferroptosis_Suppressors geneset comparing BRG1^{T910M} vs. BRG1^{WT} MINO cells. (f) Ranked CRISPR/Cas9-based viability screening in CCMCL1^{R}, JEKO1^{R}, and UPN1. (g) Immunoblot of BRG1-depleted CCMCL1^{R}, JEKO1^{R}, MAVER1 and UPN1 cells (4-day post-sgRNA expression). (h) Competition-based proliferation assays with BRM-targeting sgRNAs in Cas9-transduced JEKO1 and MINO cells (n=3). sgROSA and sgPCNA were used as negative and positive controls, respectively. (i) Immunoblot verification of BRM depletion in JEKO1 and MINO cells (4-day post-sgRNA expression). (j) Changes in lipid peroxidation following BRG1 depletion in MINO^{WT} and MINO^{T910M} cells (4-day post-sgRNA expression). (k) Immunoblot confirming BRG1 depletion in MINO^{WT} and MINO^{T910M} cells (4-day post-sgRNA expression). All IB treatments were performed at 5 μM. For non-box-plot quantitative panels, data represent mean ± s.d. from n = 5 biologically independent experiments, except n = 4 and n = 3 biologically independent experiments in (b) and (h), respectively. Box plots in (d,j) indicate median as centre lines, 25th and 75th percentiles as box bounds, and minimum and maximum values as whiskers. Points in (d) represent single-cell flow cytometry events from a representative experiment repeated independently three times with similar results; data in (j) are from n = 4 biologically independent experiments. Immunoblots in (a,g,i,k) are representative of experiments repeated independently three times with similar results. Statistical significance was determined using one-way ANOVA followed by Tukey's test.

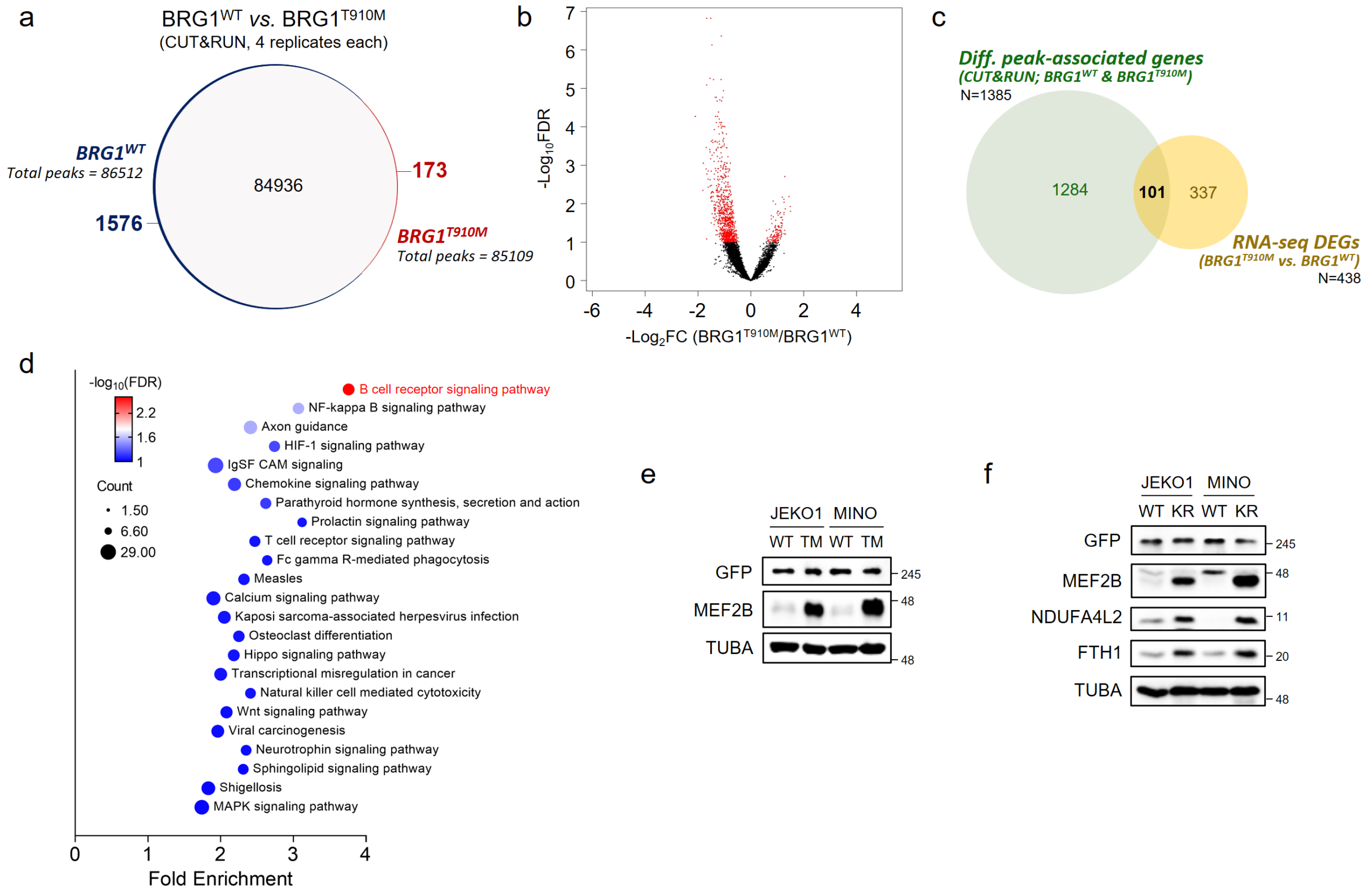

Supplementary Fig. 3. Transcriptional deregulation in BRG1^{mut}-expressing MCL cells. (a) Venn diagram illustrating the overlap of BRG1 binding sites. Anti-GFP Cut&Run sequencing was performed on four biological replicates for venus-tagged BRG1^{WT} and BRG1^{T910M} expressing MINO cells. The Venn diagram displays the high degree of conservation in chromatin occupancy between the two groups with a total of 84,936 common peaks. (b) Volcano plot displaying the peak-normalized differential binding analysis between BRG1^{T910M} and BRG1^{WT} (DESeq2). The x-axis represents Log2 fold change (FC) of BRG1 occupancy, and the y-axis is the significance. The asymmetry of the plot suggests a preferential loss of BRG1 binding intensity in the BRG1^{T910M} relative to BRG1^{WT}. (c) Venn diagram illustrating the overlap between genes associated with differential BRG1 binding sites (Cut&Run; green) and DEGs (RNA-seq; yellow) in BRG1^{T910M} and BRG1^{WT} MINO cells. (d) KEGG analysis of genes associated with differential binding between BRG1^{T910M} and BRG1^{WT}. (e-f) Immunoblot analysis of BRG1^{WT} *vs.* BRG1^{T910M} (e) or BRG1^{K785R} (f) MCL cells. Immunoblots in (e,f) are representative of experiments repeated independently three times with similar results.

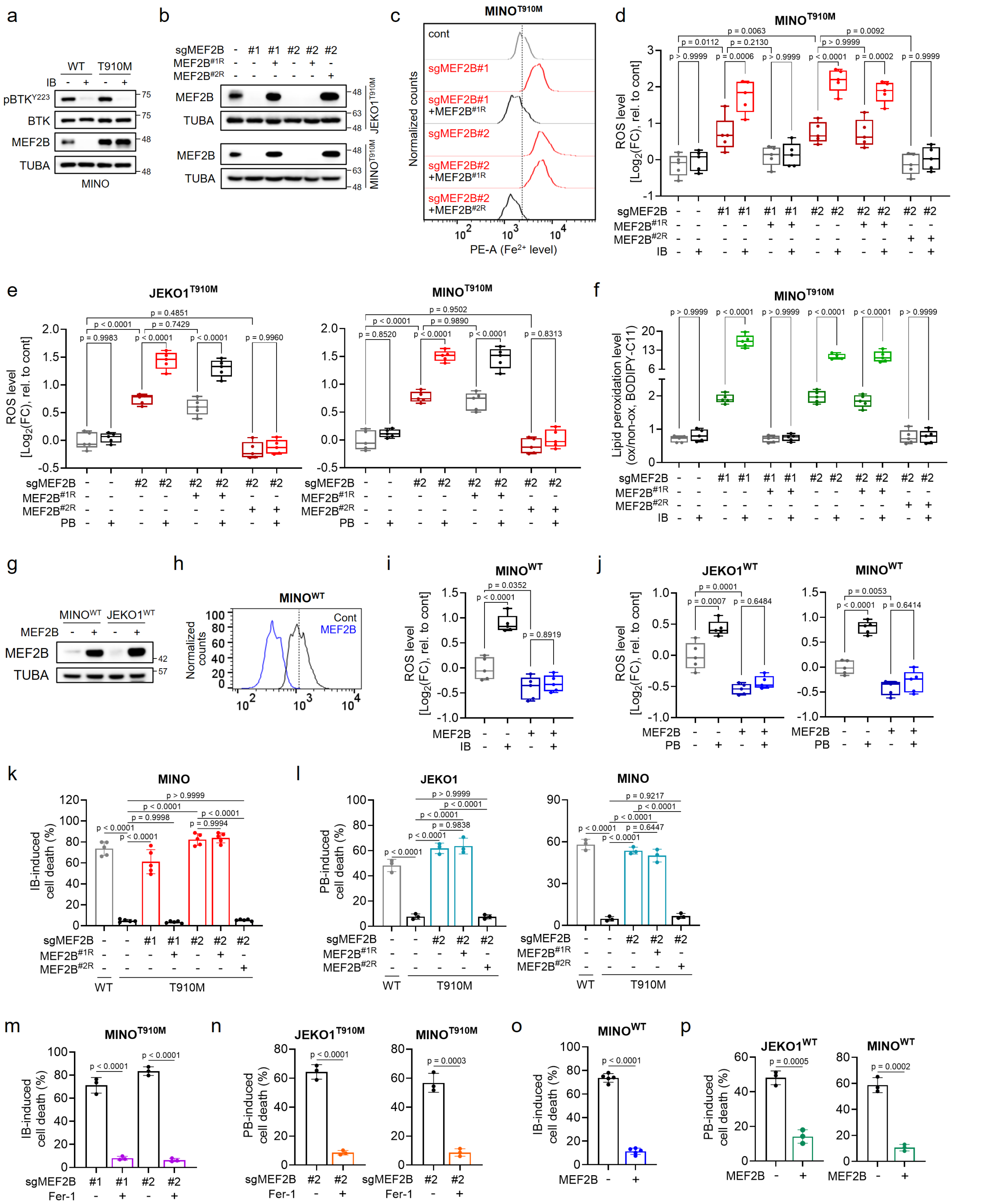

Supplementary Fig. 4. MEF2B mediates ferroptosis response downstream of BRG1. (a) Immunoblot of MINO^{WT} and MINO^{T910M} cells following IB treatment (24 h). (b) Immunoblot of MEF2B-depleted/rescued JEKO1^{T910M} and MINO^{T910M} cells. (c-d) MEF2B-depleted and rescued MINO^{T910M} cells showing basal labile Fe^{2+} (c; 48 h) and IB-induced ROS (d; 48 h) level changes. (e) PB-induced ROS (48 h) level changes in MEF2B-depleted/rescued JEKO1^{T910M} and MINO^{T910M} cells. (f) IB-induced lipid peroxidation (72 h) level changes in MEF2B-depleted/rescued MINO^{T910M} cells. (g) Immunoblot of MEF2B-overexpressed JEKO1^{WT} and MINO^{WT} cells. (h-i) MEF2B-overexpressing MINO^{WT} cells showing basal labile Fe^{2+} (h; 48 h) and IB-induced ROS (i; 48 h) level changes. (j) PB-induced ROS (48 h) level changes in MEF2B-overexpressing JEKO1^{WT} and MINO^{WT} cells. (k) IB-induced cell death (96 h) in MEF2B-depleted/rescued MINO^{T910M} cells (l) PB-induced cell death (96 h) in MEF2B-depleted/rescued JEKO1^{T910M} and MINO^{T910M} cells. (m-n) Effect of Fer-1 (2 μM) on IB- (m) and PB-induced (n) cell death in MEF2B-depleted MINO^{T910M} and/or JEKO1^{T910M} cells (96 h). (o-p) IB- (o) and PB-induced (p) cell death (96 h) in MEF2B-overexpressing MINO^{WT} and/or JEKO1^{WT} cells. All IB/PB treatments were performed at 5 μM. Quantitative data are from n = 5 biologically independent experiments, except

n = 3 biologically independent experiments in (l–n,p). Bar plots represent mean ± s.d.; box plots indicate medians as centre lines, 25th and 75th percentiles as box bounds, and minimum and maximum values as whiskers. Representative histograms in (c, h) and immunoblots in (a,b,g) are from experiments repeated independently three times with similar results. Statistical significance in (n-p) was determined using unpaired two-sided Student's t-tests; all other comparisons were analyzed using one-way ANOVA followed by Tukey's test.

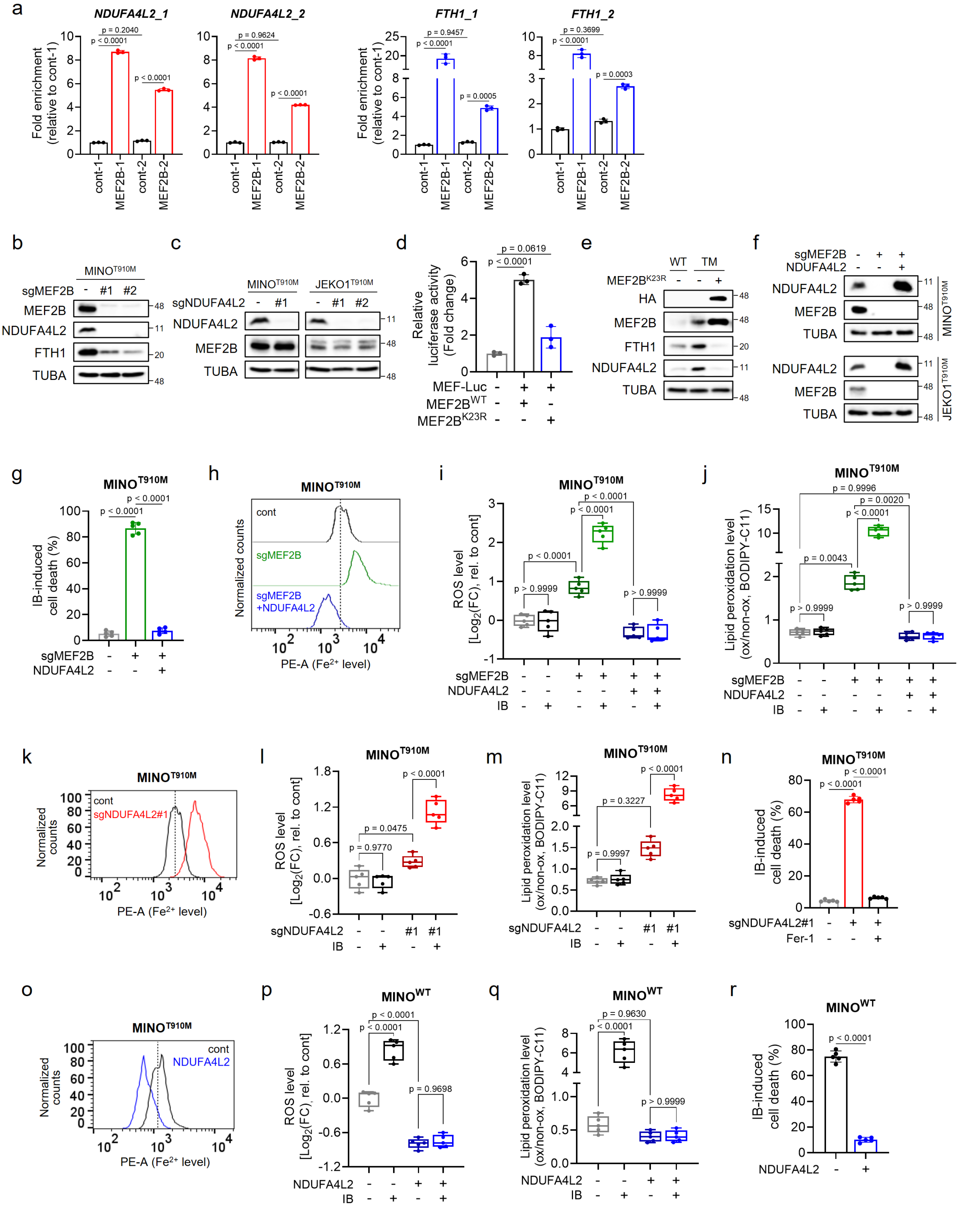

Supplementary Fig. 5. NDUFA4L2 serves as the key downstream effector of MEF2B to mediate BTKi-induced ferroptosis resistance. (a) ChIP-qPCR analysis of control and MEF2B^{WT}-expressing JEKO1 cells at the NDUFA4L2 and FTH1 loci. Enrichment was normalized to gene desert region (n=3). (b-c) Immunoblot of MEF2B-depleted MINO^{T910M} cells (b), NDUFA4L2-depleted MINO^{T910M} and JEKO1^{T910M} cells (c). (d) Relative luciferase activity of a MEF2 reporter in 293T cells transiently expressing MEF2B^{WT} or MEF2B^{K23R} (24 h post-transfection) (e) Immunoblot analysis comparing JEKO1^{WT}, JEKO1^{T910M}, and MEF2B^{K23R}-expressing JEKO1^{T910M} cells. (f) Immunoblot of MEF2B-depleted MINO^{T910M} and JEKO1^{T910M} cells reconstituted with NDUFA4L2. (g) IB-induced cell death (96 h) in MEF2B-depleted MINO^{T910M} cells with or without NDUFA4L2 rescue. (h-j) Basal Fe^{2+} (h), IB-induced ROS (i; 48 h), and IB-induced lipid peroxidation (j; 72 h) in MEF2B-depleted MINO^{T910M} cells with or without NDUFA4L2-reconstitution. (k-m) Basal Fe^{2+} (k), IB-induced ROS (l; 48 h), and IB-induced lipid peroxidation (m; 72 h) in NDUFA4L2-depleted MINO^{T910M} cells. (n) Effect of Fer-1 (2 μM) on IB-induced cell death in NDUFA4L2-depleted MINO^{T910M} cells (96 h). (o-q) Basal Fe^{2+} (o), IB-induced ROS (p; 48 h), and IB-induced lipid peroxidation (q; 72 h) in NDUFA4L2-

overexpressing $MINO^{WT}$ cells. (r) IB-induced cell death in NDUFA4L2-overexpressed $MINO^{WT}$ cells (96 h). All IB treatments were performed at 5 μM. Quantitative data are from n = 5 biologically independent experiments, except n = 3 biologically independent experiments in (a,d). Bar plots represent mean ± s.d.; box plots indicate medians as centre lines, 25th and 75th percentiles as box bounds, and minimum and maximum values as whiskers. Immunoblots in (b,c,e,f) and representative histograms in (h,k,o) are from experiments repeated independently three times with similar results. Statistical significance in (n,r) was determined using unpaired two-sided Student's t-tests; all other comparisons were analyzed using one-way ANOVA followed by Tukey's test.

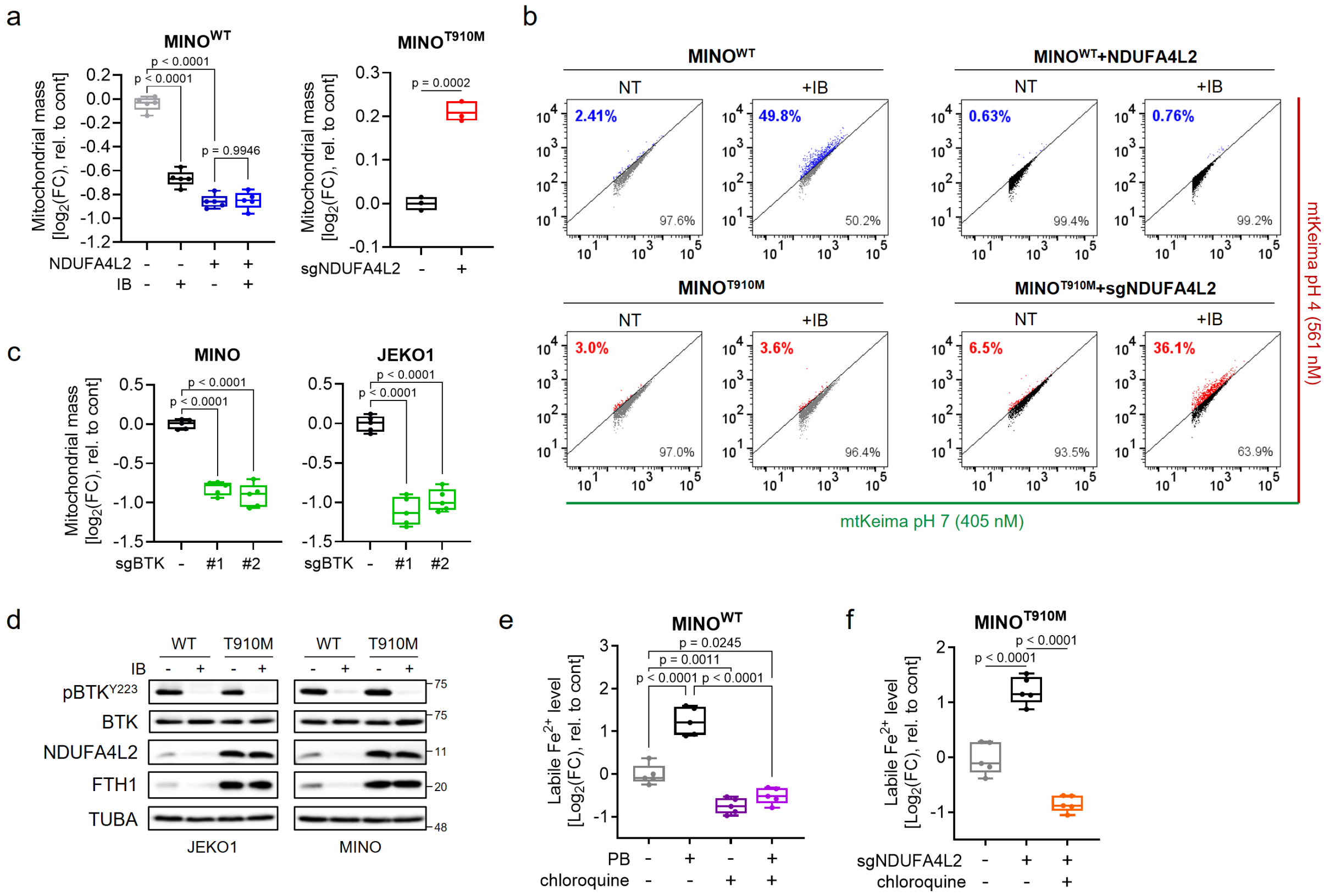

Supplementary Fig. 6. NDUFA4L2 regulates mitochondrial activity to promote ferroptosis resistance. (a-b) Relative mitochondrial mass (a) and mitophagy levels (b) in MINO^{WT} (control *vs.* NDUFA4L2-overexpressed; n=5) and MINO^{T910M} (control vs. NDUFA4L2-depleted; n=3) cells with or without IB treatment (24 h). (c) Relative mitochondrial mass in BTK-depleted MCL cells (6-day post sgRNA expression). (d) Immunoblot of BRG1^{WT} or BRG1^{T910M}-expressing JEKO1 and MINO cells with or without IB treatment (24 h). (e-f) Labile Fe^{2+} levels in: (e) MINO^{WT} cells treated with PB and/or chloroquine (50 μM), and (f) NDUFA4L2-depleted MINO^{T910M} cells treated with chloroquine (50 μM) (24 h; n=5). All IB/PB treatment was performed at 5 μM. All n values denote biologically independent experiments. Bar plots represent mean ± s.d.; box plots indicate medians as centre lines, 25th and 75th percentiles as box bounds, and minimum and maximum values as whiskers. Immunoblot in (d) is representative of experiments repeated independently three times with similar results. Statistical significance was assessed by unpaired two-sided Student's t-test (two groups) or one-way ANOVA followed by Tukey's test (multiple groups).

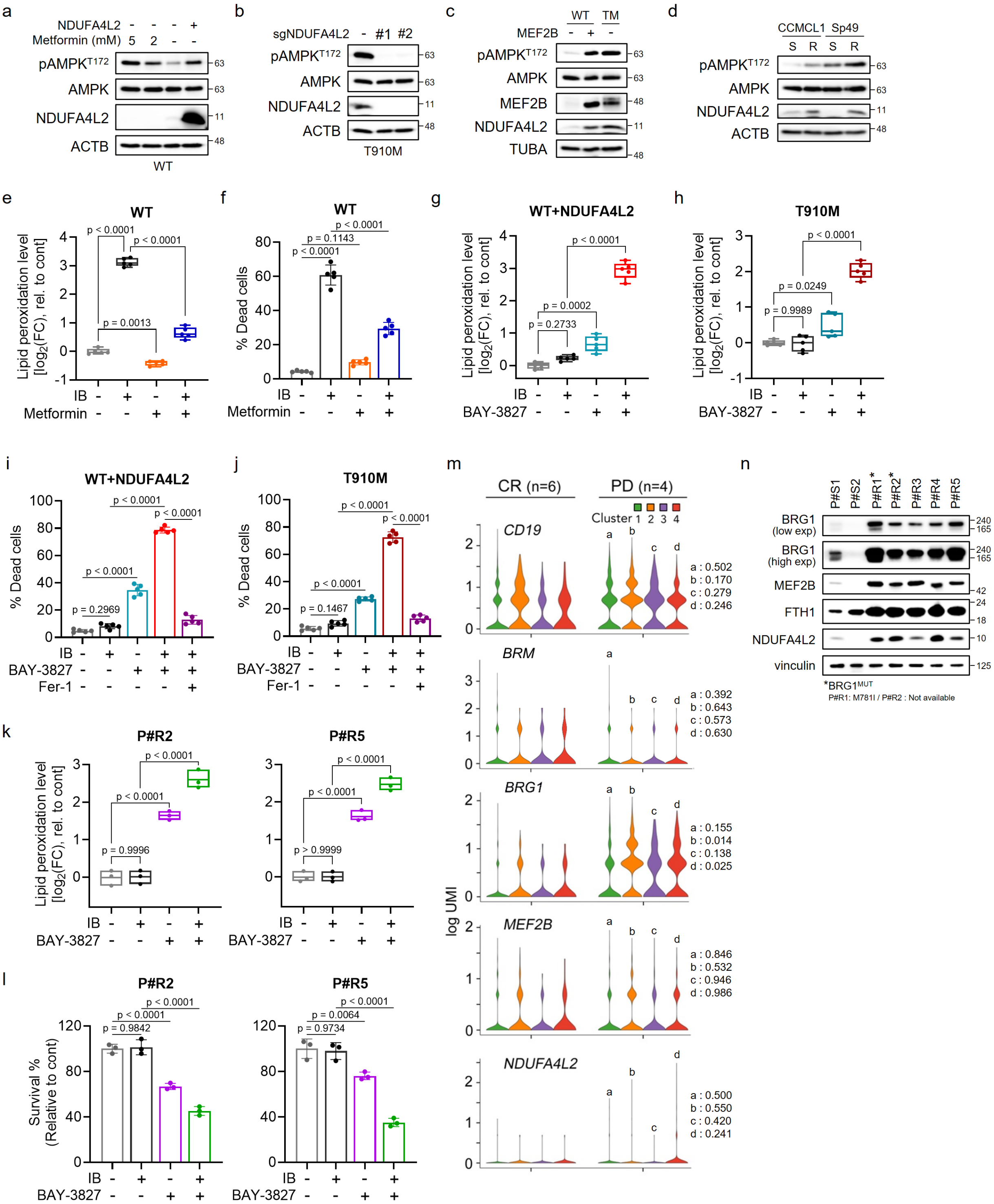

Supplementary Fig. 7. NDUFA4L2 induces AMPK activation to confer BTKi-induced ferroptosis resistance. (a) Immunoblot analysis comparing control and NDUFA4L2-overexpressing JEKO1^{WT} cells. Metformin (2 or 5 mM, 24 h) was used as a positive control for AMPK activation. (b) Immunoblot analysis of NDUFA4L2-depleted JEKO1^{T910M} cells. (c) Immunoblot analysis comparing control and MEF2B-overexpressing JEKO1^{WT} and JEKO1^{T910M} cells. (d) Immunoblot analysis of isogenic BTKi^{S} and BTKi^{R} CCMCL1 and Sp49 cells. (e, f) Changes in lipid peroxidation (e; 72 h) and cell death (f; 96 h) following treatment with IB (5 µM), metformin (5 mM), or their combinations in JEKO1^{WT} cells. (g-j) Changes in lipid peroxidation (72 h) and cell death (96 h) following IB (5 µM), BAY-3827 (5 µM) or their combinations in NDUFA4L2-overexpressing JEKO1^{WT} (g, i) and JEKO1^{T910M} (h, j) cells. Fer-1 co-treatment (2 µM) was used to assess ferroptotic cell death. (k, l) Changes in lipid peroxidation (48 h) and cytotoxicity (96 h) following treatment with IB (5 µM), BAY-3827 (5 µM), or their combinations in BTKi^{R} primary MCL cultures. (m) Violin plots display the expression levels of indicated genes in MCL cells (samples as described in **Fig. 7b**). To the right, p-values indicate the results of a two-tailed Student's t-test comparing the percentage of cells expressing each gene between patients with CR

(n=4 independent patient specimens) and PD (n=6 independent patient specimens). (n) Immunoblot of IB-sensitive or -resistant primary MCL cultures. Quantitative data are from n = 5 biologically independent experiments in (e–j). In (k,l), two independent patient-derived primary MCL samples were analyzed, each in technical triplicate. Bar plots represent mean ± s.d.; box plots, where shown, indicate medians as centre lines, 25th and 75th percentiles as box bounds, and minimum and maximum values as whiskers. Immunoblots in (a–d,n) are representative of experiments repeated independently three times with similar results. Unless otherwise indicated, statistical significance was determined using one-way ANOVA followed by Tukey's test.

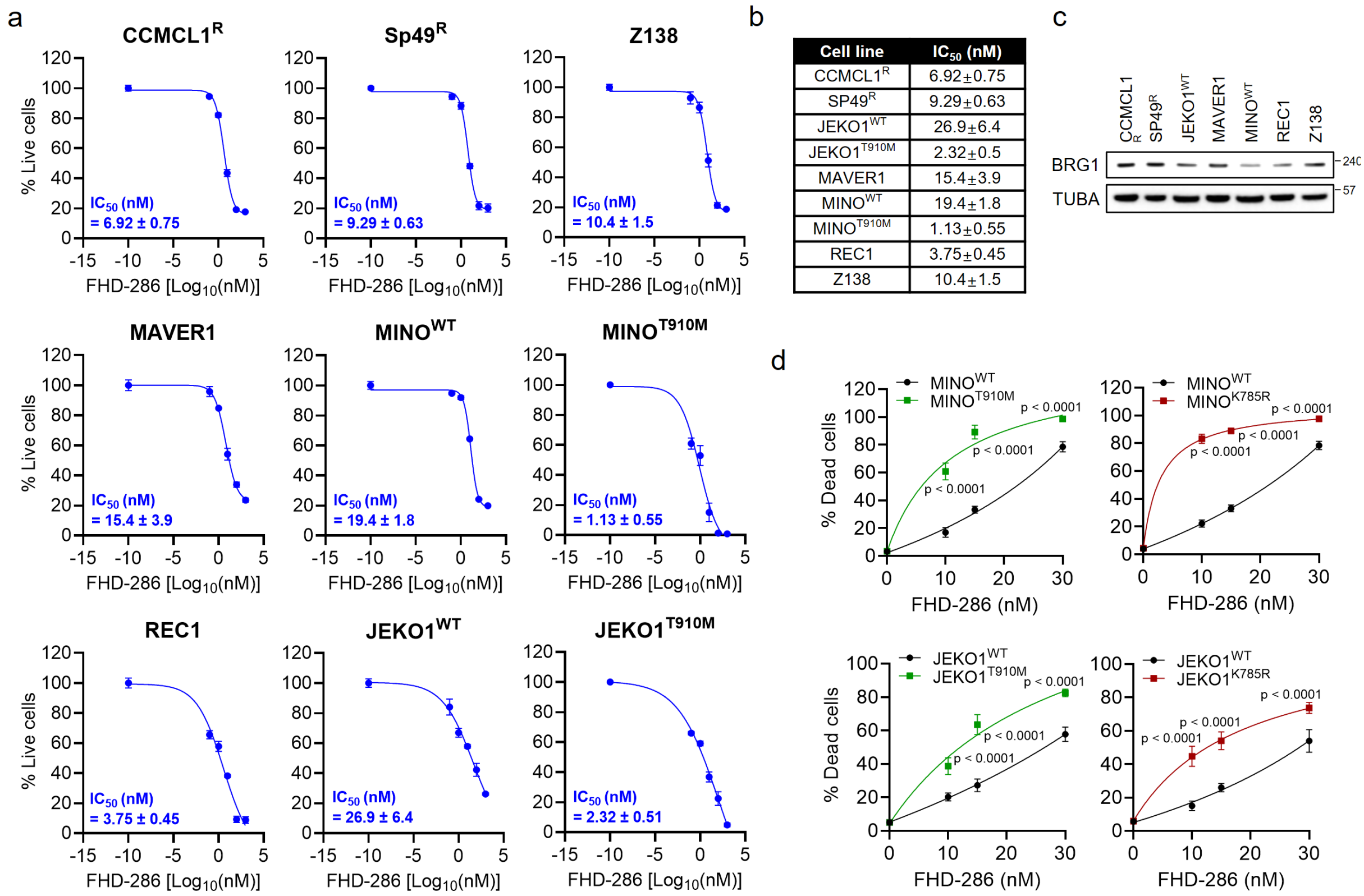

Supplementary Fig. 8. BRG1 inhibition induces cell death in MCL cells. (a) Dose-response curve of FHD-286 in multiple MCL cell lines (96 h, n=3). (b) IC_{50} value of FHD-286 against different MCL cell lines (96 h, n=3). (c) Immunoblot analysis of BRG1 protein expression level in MCL cell lines. (d) Dose-response curve comparing FHD-286-induced cell death in $BRG1^{WT}$ vs. $BRG1^{T910M}$ or $BRG1^{K785}$ MCL cell lines (96 h, n=5). All n values denote biologically independent experiments. Immunoblot in (c) is representative of experiments repeated independently three times with similar results. Significance was determined using two-way ANOVA with Tukey's test.

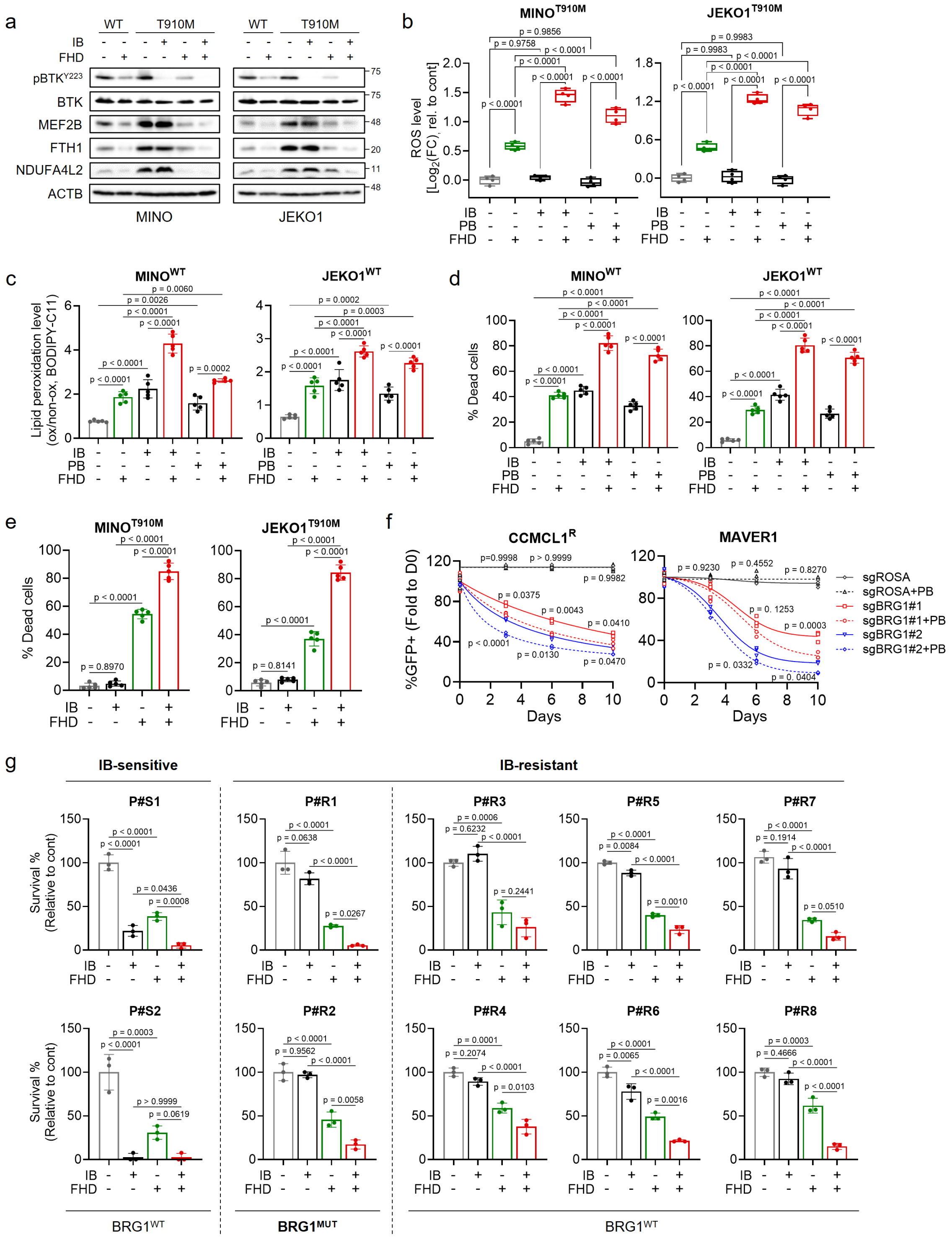

Supplementary Fig. 9. BRG1 inhibition re-sensitizes resistant MCL cells to BTKi. (a) Immunoblot of BRG1^{WT} and BRG1^{T910M} cells treated with either IB, FHD-286, or their combinations for 24 h. (b) Changes in ROS (48 h) following treatment with IB, PB, FHD-286 or their combinations in MINO^{T910M} and JEKO1^{T910M} cells. (c-d) Lipid peroxidation (48 h) (c) and cell death (72 h) (d) following treatment with IB, PB, FHD-286, or their combinations in MINO^{WT} and JEKO1^{WT} cells. (e) Cell death effect of IB, FHD-286 or their combination in MINO^{T910M} and JEKO1^{T910M} cells (72 h) MCL cells. (f) Competition-based proliferation assays of sgBRG1 in Cas9-transduced CCMCL1^{R}, MAVER1 cells with or without PB (2 μM). sgROSA was used as a negative control. (g) Relative cell survival rate following treatment with IB (5 μM), FHD-286 (30 nM), or their combinations in primary MCL cultures (96 h). Quantitative data are from n = 5 biologically independent experiments in (b–e) and n = 3 biologically independent experiments in (f). In (g), ten independent primary MCL culture were analyzed, each in technical triplicate. Bar plots represent mean ± s.d.; box plots, where shown, indicate medians as centre lines, 25th and 75th percentiles as box bounds, and minimum and maximum values as whiskers. Immunoblot in (a) is representative of experiments repeated independently three times with similar results. Statistical significance was determined using one-way or two-way ANOVA followed by Tukey's test.

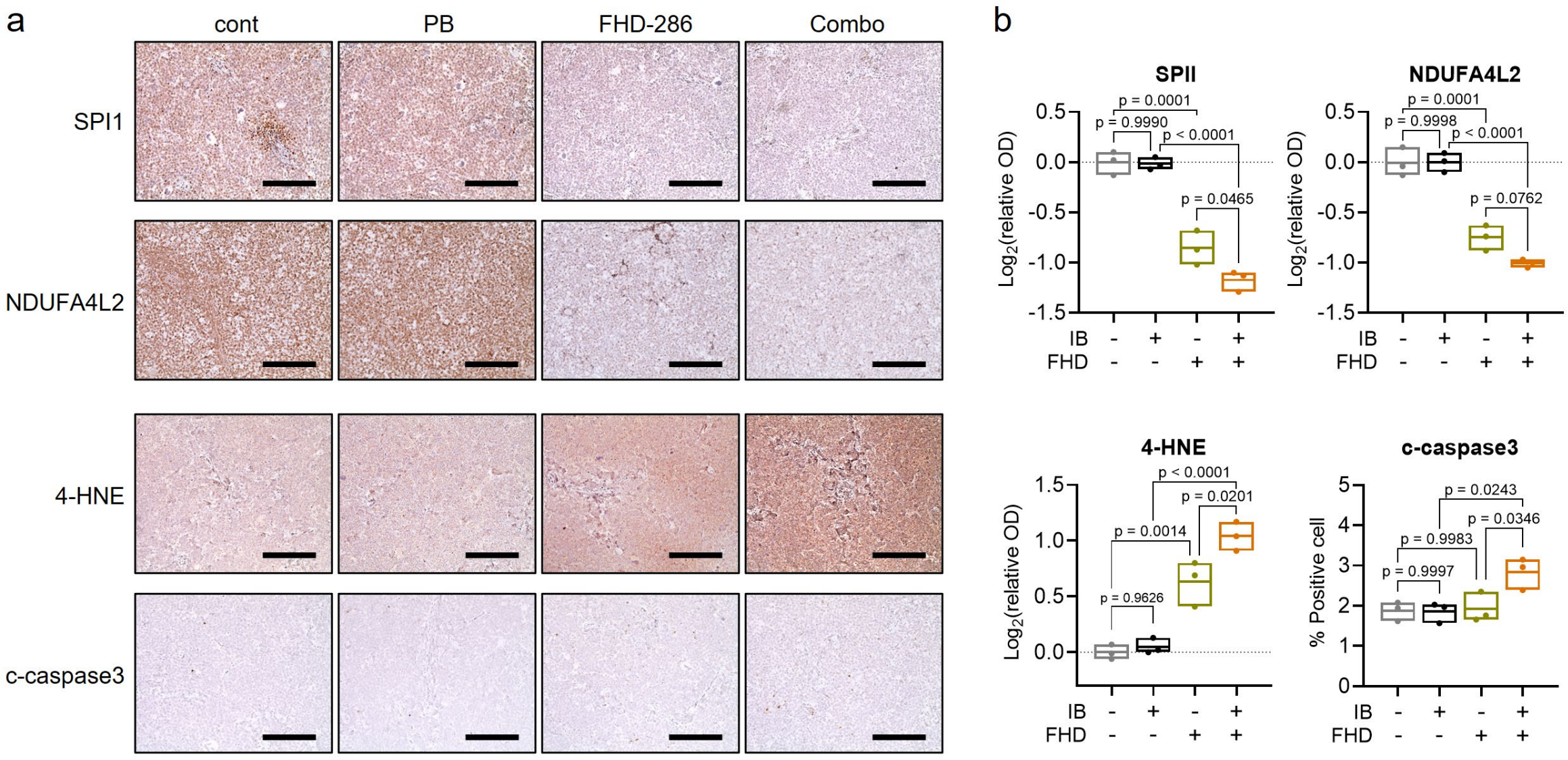

Supplementary Fig. 10. BRG1 inhibition re-sensitizes resistant MCL xenograft to BTKi. (a) Representative IHC images of tumor sections for SPI1, NDUFA4L2, 4-HNE, and cleaved caspase 3 in the indicated treatment groups. Scale bars, 200 µm. (b) Quantification of IHC staining in the tumor sections shown in (a). Data represent mean ± s.d. from n=3 tumors collected from independent mice per group; multiple fields per tumor were averaged before statistical analysis. Significance was determined using one-way ANOVA followed by Tukey's test.

Supplementary Table 1. All reagents, cell lines, oligonucleotides, recombinant DNA, software, algorithms and instruments used in the study

REAGENT	SOURCE	IDENTIFIER
**Antibodies**		
HA-tag C29F4 (Cut&Run/WB)	Cell Signaling Technologies	Cat# 3724
H3K27Ac (Cut&Run)	Abcam	Cat# ab4729
BRG1 (Cut&Run)	EpiCypher	Cat# 13-2002
Normal IgG (Cut&Run)	EpiCypher	Cat# 12709
Goat anti-rabbit IgG (Cut&Run)	ThermoFisher Scientific	Cat# ABE1294
GFP (Venus) (Cut&Run/WB)	Abcam	Cat# ab290
BRG1	Cell Signaling Technology	Cat# 49360
NDUFA4L2	ThermoFisher Scientific	Cat# 16480-1-AP
FTH1	Cell Signaling Technology	Cat# 4393
BTK	Cell Signaling Technology	Cat# 8547
pBTK (Y223)	Cell Signaling Technology	Cat# 87457
MEF2B	Santa Cruz Biotechnology	Cat# sc-517433
Alpha Tubulin (TUBA)	Cell Signaling Technology	Cat# 2144
PLCγ2	Cell signaling Technology	Cat# 3872
pPLCγ2 (Y1217)	Cell signaling Technology	Cat# 3871
SPI1	Cell signaling Technology	Cat# 2258
ACTB	Santa Cruz Biotechnology	Cat# sc-47778
Vinculin	Cell signaling Technology	Cat# 13901
4-Hydroxynonenal (4-HNE)	Novus Biologicals	Cat# MAB3249-SP
Cleaved Caspase 3 (Asp175)	Cell Signaling Technology	Cat# 9661
Goat anti-mouse IgG HRP	ThermoFisher Scientific	Cat# 31430
Goat anti-rabbit IgG HRP	ThermoFisher Scientific	Cat# 31463
**Bacterial Strain**		
Stable Competent E. coli	New England BioLabs	Cat# C3040H
**Chemicals and biologicals**		
Dulbecco's Modification of Eagle's Medium (DMEM)	Corning	Cat# 10-013-CV
RPMI 1640	ThermoFisher scientific	Cat# 11875-093
GlutaMAX	ThermoFisher scientific	Cat# 35050061
Penicillin/Streptomycin	ThermoFisher scientific	Cat# 10378016
USDA approved fetal bovine serum (FBS)	Avantor Seradigm	Cat# 89510-186
Trypsin EDTA	Corning	Cat# 25-053-CI
Puromycin	InvivoGen	Cat# anti-pr-1
Blasticidin	InvivoGen	Cat# anti-bl-1
Geneticin® Selective Antibiotic (G418 Sulfate)	Thermo Fisher Scientific	Cat# 11811031
Polyethyleneimine (PEI)	Polysciences Inc	Cat# 23966-1
Opti-MEM™ I Reduced Serum Medium	Thermo Fisher Scientific	Cat# 31985070
SPRIselect Reagent	Beckman Coulter	Cat# B23317
CloneAmp™ HiFi PCR Premix	Clontech	Cat# 639298
Gibson Assembly Mix	New England BioLabs Inc.	Cat# E2611L
Q5®High-Fidelity 2X mix	New England BioLabs Inc.	Cat# M0492L
Ferrostatin-1	Cayman Chemical	Cat# HY-100579
Ibrutinib	MedChemExpress	Cat# HY-10997

Pirtobrutinib	MedChemExpress	Cat# HY-131328
Zanubrutinib	MedChemExpress	Cat# HY-101474A
FHD-286	FoghornTherapeutics	NA
Q-VD-OPH	Cayman Chemical	Cat# 15260
Z-VAD-FMK	MedChemExpress	Cat# HY-16658B
Necrostatin-2	MedChemExpress	Cat# HY-14622
Erastin	Cayman Chemical	Cat# 17754
BAY-3	MedChemExpress	Cat# HY-112083
Metformin	MedChemExpress	Cat# HY-B0627
CellROX™ Deep Red	ThermoFisher Scientific	Cat# C10422
MitoTracker™ deep red FM	ThermoFisher Scientific	Cat# 46753
FerroOrange	Cell Signaling Technology	Cat# 36104
BODIPY-C11	Cayman Chemical	Cat# 27086
BODIPY 665/676	ThermoFisher Scientific	Cat# B3932
N-acetyl-L-cysteine (NAC)	Millipore Sigma	Cat# 106425
To-Pro-3	ThermoFisher Scientific	Cat# T3605
XTT sodium	Gold Bio	Cat# X-200-100
D-Luciferin, Potassium Salt	Gold Bio	Cat# LUCK
NucleoBond Xtra Midi kit	Macherey-Nagel (MN)	Cat# 740410.50
NucleoSpin Plasmid EasyPure	Macherey-Nagel (MN)	Cat# 74027.25
NucleoSpin RNA kit	Macherey-Nagel (MN)	Cat# 740955.25
PowerUP SYBR Green Mix	ThermoFisher Scientific	Cat# A25778
RevertAid RT Reverse Transcription kit	ThermoFisher Scientific	Cat# K1691
NucleoSpin Gel and PCR clean-up DNA extraction kit	Macherey-Nagel (MN)	Cat# 740609.25
16% paraformaldehyde	ThermoFisher Scientific	Cat# 28908
Dynabeads® Protein G	ThermoFisher Scientific	Cat# 1009D
2-Mercaptoethanol	Sigma-Aldrich	Cat# M6250
Bovine Serum Albumin	Sigma-Aldrich	Cat# A3059
Pierce BCA protein assay kit	ThermoFisher Scientific	Cat# 23225
PVDF transfer membrane	ThermoFisher Scientific	Cat# 88518
SuperSignal West Femto Chemiluminescent substrate	ThermoFisher Scientific	Cat# 34096
CUTANA™ ChIC/CUT&RUN Kit	EpiCypher	Cat# 14-1048
CUTANA™ CUT&RUN library Kit	EpiCypher	Cat# 14-1002
**Experimental Models: Cell Lines**		
CCMCL1	Chen-Kiang Laboratory	CVCL_RU10
JEKO1	ATCC	CRL-3006
UPN1	Parekh Lab	CVCL_A795
MAVER1	ATCC	CRL-3008
Z138	ATCC	CRL-3001
MINO	ATCC	CRL-3000
REC1	ATCC	CRL-3004
HEK293T	ATCC	CRL-11268
Sp49	Sehgal Laboratory	CVCL_D022
**Oligonucleotides (synthesized by Integrated DNA Technologies)**		
sgMEF2B#1 (target sequence)	GAGTCACTTACATACAGCCG	
sgMEF2B#2 (target sequence)	GGAGATGGGCGGCTCTGGGC	
sgNDUFA4L2#1 (target sequence)	GCAGATCAAAAGACATCCGG	
sgNDUFA4L2#2 (target sequence)	CTGGGACAGAAAGAACAACC	
sgBRG1#1 (target sequence)	CGTCCTTCTCTAGGTCGTTG	
sgBRG1#2 (target sequence)	GGATGCCGTGATCAAGTACA	

sgBRG1#3 (target sequence)	CGTTGAGGCTGCGGTACTTG	
sgROSA (target sequence)	GAAGATGGGCGGGAGTCTTC	
sgPCNA (target sequence)	GGACTCGTCCCACGTCTCTT	
sgBTK#1 (target sequence)	AGCGGTGGCGCATCTCCCTC	
sgBTK#2 (target sequence)	TCTTGATGGCCACGTCGTAC	
sgSOX11#1(target sequence)	CGATGAACGCGTTCATGGTA	
sgSOX11#2 (target sequence)	CGAGAAGATCCCGTTCATC	
qPCR-hDesert-F	TGG CCT GTT TCT AGG AGT GA	
qPCR-hDesert-R	AGG ACA CAG AGA GCA GAG GA	
qPCR-FTH1#1-F	GTT TAG TGG AGT TGG AGG GAA G	
qPCR-FTH1#1-R	AAA TGG GAG CCG AAT CAG G	
qPCR-FTH1#2-F	CTC TGA GTC CTG GTG GTA GTT	
qPCR-FTH1#2-R	CGC CGC CTC TCC TTA GT	
qPCR-NDUFA4L2#1-F	TGT CCA TCT CTC TCT TCC CTA TT	
qPCR-NDUFA4L2#1-R	CGA GAC ACA AAG AGA CAC AGA A	
qPCR-NDUFA4L2#2-F	GGG CTG CAG ATT CGA GAT T	
qPCR-NDUFA4L2#2-R	GAG ACA GGA AGC AGA CAG AAG	
**Recombinant DNA**		
pMD2.G	Trono Lab	Addgene #12259
psPAX2	Trono Lab	Addgene #12260
LentiCrisprV2		Addgene #52961
LRG2.1 (U6-sgRNA-GFP)		Addgene #108098
pLU-HA-MEF2B		Homemade
pLU-NDUFA4L2		Homemade
pPB CAG SMARCA4 T910M-Venus	Luca Tiberi lab	Addgene #15395; http://n2t.net/addgene:153951; RRID:Addgene_153951
pPB CAG SMARCA4-Venus	Luca Tiberi lab	Addgene #153950; http://n2t.net/addgene:153950; RRID:Addgene_153950
PiggyBac Transposase	System Biosciences	Cat.# PB220PA-1
pHAGE-mt-mKeima	Richard Youle lab	Addgene #131626; http://n2t.net/addgene:131626; RRID:Addgene_131626
**Software, Algorithms and Instruments**		
GraphPad Prism		Version 10
ImageJ		https://imagej.nih.gov/ij/
7500 fast software	ThermoFisher Scientific	Version2.3
7500 Fast Real-time PCR system		Cat# 4351106
EVOS FL Cell Auto Imaging System	ThermoFisher Scientific	Cat# AMAFD1000
LSR II Flow Cytometer	BD Biosciences	Cat# 642221
M220 Focused-ultrasonicator	Covaris	
IVIS Spectrum Image Analysis	PerkinElmer Inc	Cat# 124262
Living Image®	PerkinElmer Inc	Version 4.7.3
Azure 400	Azure Biosystems	
