# Supplementary material for: BRG1-mediated suppression of ferroptosis underlies BTK inhibitor resistance
Source: Nat Commun. 2026 Jul 2;17:8220. doi: 10.1038/s41467-026-75123-4 (PMC13462549; doi:10.1038/s41467-026-75123-4)
Supplement: Supplementary file 4 [file 41467_2026_75123_MOESM2_ESM.pdf]

Title: Supplementary Data 1.

Description: List of ferroptosis suppressor genes used for GSEA (curated form FerrDb v2; 177 genes).

Title: Supplementary Data 2.

Description: CRISPR/Cas9-based vulnerability screen targeting 182 epigenetic regulators (~1,461 sgRNAs). Gene-level fitness scores were determined by calculating the mean Log2 fold change of gRNA abundance between the initial population (Start) and the final harvested population (End).

Title: Supplementary Data 3.

Description: Differentially expressed genes from PRO-seq in FHD-286-treated versus control cells.

Title: Supplementary Data 4.

Description: Differentially expressed genes from RNA-seq in FHD-286-treated versus control cells.

Title: Supplementary Data 5.

Description: BRG1 CUT&RUN peaks identified relative to IgG control in BRG1WT cells.

Title: Supplementary Data 6.

Description: Differentially expressed genes from RNA-seq in BRG1T910M versus BRG1WT cells.

Title: Supplementary Data 7.

Description: Differential anti-GFP CUT&RUN peaks in BRG1T910M versus BRG1WT cells (UP).

Title: Supplementary Data 8.

Description: Differential anti-GFP CUT&RUN peaks in BRG1T910M versus BRG1WT cells (DN).

Title: Supplementary Data 9.

Description: Differentially expressed genes from RNA-seq in control versus MEF2B-depleted cells.

Title: Supplementary Data 10.

Description: HA-MEF2B CUT&RUN peaks identified relative to non-expressing control cells.
